# Catalyst-free photoinduced dehalogenation and functionalization of alkyl halides using Lewis bases

**DOI:** 10.1039/d5ra07627g

**Published:** 2025-11-05

**Authors:** Krishnakumar Sachidanandan, Anulika Umunnah, Alexis T. Hahn, Sébastien Laulhé

**Affiliations:** a Department of Chemistry & Chemical Biology, Indiana University Indianapolis Indianapolis Indiana 46202 USA slaulhe@iu.edu

## Abstract

Alkyl halides are foundational building blocks in organic synthesis and valuable commodity chemicals. While numerous pathways have been developed to functionalize these groups into higher value compounds, most of the methods require the use of transition metals and associated ligands, which increase the footprint of these transformations. Simultaneously, dehalogenation of these materials to their corresponding C(sp^3^)–H forms has been limited despite the regulatory incentives to phase-out the use of halogenated compounds due to their innate toxicities and environmental impact. As such, the upcycling of alkyl halides and forever chemicals, *via* functionalization or dehalogenation strategies, needs the development of novel methods that are sustainable and cost-effective. Herein, we present a photoinduced functionalization and defunctionalization of alkyl halides using Hünig's base (diisopropylethylamine). This protocol can successfully reduce, chalcogenate, and borylate a broad range of aliphatic halides. Emphasizing the low footprint of this reaction, the transformation only requires a commodity Lewis base, a green solvent, and light, thereby offering a more sustainable alternative to conventional pathways.

## Abstract 2

A photoinduced functionalization and defunctionalization of alkyl halides using Hünig's base (diisopropylethylamine) is presented. This protocol successfully reduces, chalcogenates, and borylates a broad range of aliphatic halides.

## Graphical abstract

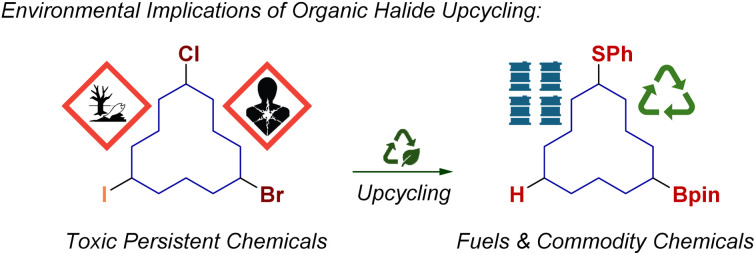

## 1. Introduction

**Scheme 1:**
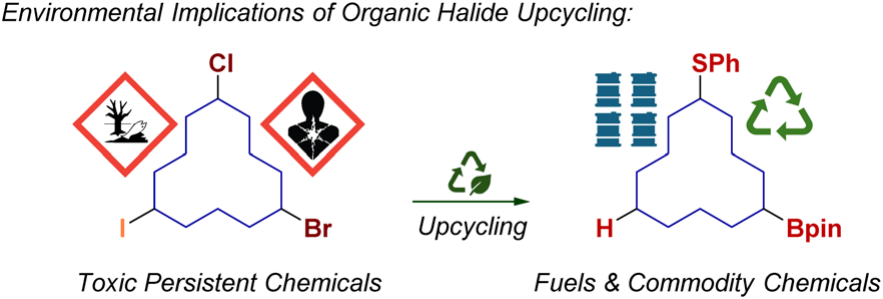
Conceptual blueprint on organic halide upcycling.

In spite their incredible utility in the chemical sciences, multiple regulations have been advocating for the phasing-out of alkyl halides due to their multifaceted hazardous nature;^1^ from the ozone depleting activities of chlorofluorocarbons (CFCs) and the soil contamination of dichlorodiphenyltrichloroethane (DDT), to the human toxicity of forever chemicals and PFOAs.^2^ Therefore, transforming these materials into higher value-added compounds through reductive dehalogenation or functional group interconversion serve as methods to repurpose these persistent and toxic molecules (Scheme 1).

Of particular interest, reductive dehalogenation is attractive as it offers a pathway to defunctionalize a wide range of compounds containing alcohols, carbonyls, and carboxylic groups, which are found in biomass waste. Indeed, these functional groups can be readily transformed into their corresponding alkyl halides in no more than two synthetic steps.^3^ As such, sustainable transformations of alkyl halides enable the upcycling of highly functionalized biomass waste^4^ and the detoxification of persistent halogenated chemicals,^5^ offering pathways to fuels or valuable feedstock compounds. Traditional strategies for replacing a C(sp^3^)–X bond with a hydrogen atom typically depend on reductive elimination processes that require high temperatures and pressures, expensive transition metals, and stoichiometric quantities of hydride or hydrogen atom donors.^6^ Since the advent of photochemical methodologies, various approaches have been developed to achieve such defunctionalizations.^7^ These protocols often depend on the use of organic or organometallic photocatalysts, additives, and sacrificial reductants, oxidants or otherwise limited to activated alkyl halides.

Such complex systems contribute to the high cost and low sustainability of these transformations, which limits their widespread implementation.

**Scheme 2:**
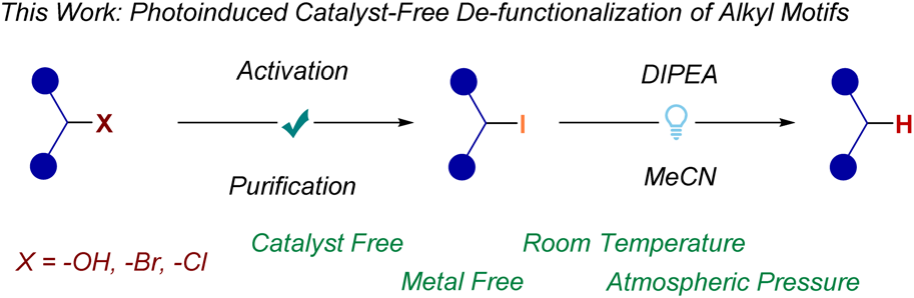
This work.

Recently, halogen-atom transfer (XAT) pathways have been widely applied for the functionalization of organic halides.^8^ Notably, catalyst-free XAT activation has enabled a broad range of transformations *via* radical-chain propagation strategies.^9^ As such, self-sustaining XAT technologies are promising photochemical methods for the upcycling of alkyl halides. Herein, we report a Lewis base-promoted dehalogenation and functionalization of organic halides under visible light irradiation (Scheme 2). The method exploits the XAT activity of α-amino radicals to abstract C(sp^3^)–X bonds and generate the corresponding carbon radical, which is then either (i) reduced to the C(sp^3^)–H bond, or (ii) functionalized *via* chalcogenation or borylation. This strategy is also applied sequentially to alcohols, chlorides, and bromides after they have been iodinated *via* well-established Appel or Finkelstein reactions.^3*b*,10^

## 2. Results and discussion

Optimization of the protocol commenced with *tert*-butyl 3-iodoazetidine-1-carboxylate (1a) as the model alkyl iodide, affording *tert*-butyl azetidine-1-carboxylate (1) as the corresponding dehalogenated product. Optimal results were obtained by employing 2 equivalents of diisopropylethylamine (DIPEA) in acetonitrile under a 440 nm light source in an argon atmosphere for 24 hours, affording product 1 in 99% yield (Table 1, entry 1). In contrast to metal hydride reductions, which use peroxide-forming ethereal solvents, acetonitrile represents a safer and more environmentally benign alternative. The presence of air (entry 2) led to a small reduction in yield to 90%, demonstrating the robustness of this transformation. However, substituting blue light with higher energy purple light had a detrimental effect on yield (73%) (entry 3). Similarly, replacing DIPEA with 1,4-diazabicyclo2.2.2octane (DABCO) completely suppressed the reaction (entry 4), and inorganic bases such as K_2_CO_3_ (entry 5) did not generate the desired product either. Finally, control reactions conducted in the absence of DIPEA (entry 6) or light (entry 7) yielded only trace amounts of the desired product (see SI-7 for additional optimization tables and related details).

**Table 1:** Optimization of standard conditions^a^

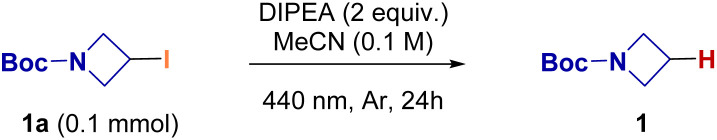		
Entry	Variation from standard condition	Yield of 1 (%)
1	—	99
2	Air instead of argon	90
3	390 instead of 440 nm	73
4	DABCO instead of DIPEA	Trace
5	K_2_CO_3_ instead of DIPEA	Trace
6	Without DIPEA	Trace
7	Without light	Trace

a ^1^H NMR yields using 1,2-dibromoethane as internal standard.

**Scheme 3:**
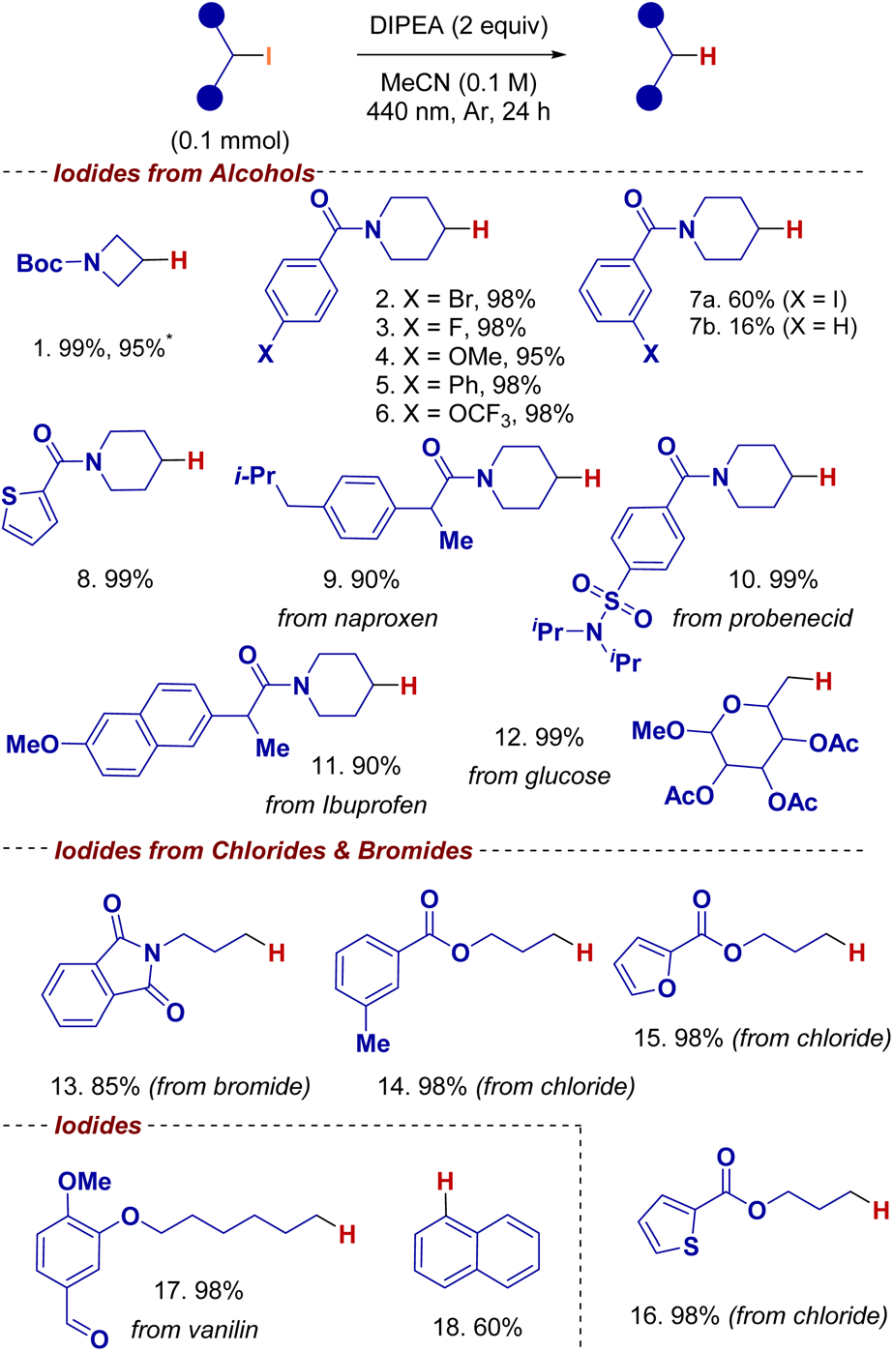
Dehalogenation substrate scope. Conditions: alkyl iodide (0.1 mmol), DIPEA (0.2 mmol, 2 equiv.), MeCN (0.1 M), 440 nm LED, 24 h. * 1.00 mmol scale.

With the optimized conditions in hand, we explored the substrate scope, beginning with the defunctionalization of alkyl iodides obtained from their corresponding alcohols *via* the Appel reaction (Scheme 3). Substrates bearing *N*-boc protecting groups, as well as aryl bromide and aryl fluoride substituents, were well tolerated under the reaction conditions, affording the defunctionalized products 1, 2, and 3 in excellent yields (99%, 98%, and 99%, respectively). Furthermore, substrates featuring aryl ethers, biphenyl motifs, and trifluoromethoxy groups delivered the desired products 4, 5, and 6 in similarly high yields (95%, 98%, and 98%) underscoring the broad functional group compatibility of this method relative to conventional photocatalytic approaches. Substrates bearing aryl iodide substituents afforded a mixture of aliphatic and aromatic defunctionalized products (7a and 7b), with the transformation favoring aliphatic iodide activation. Sensitive to addition reactions, the thiophene moiety was well-tolerated and afforded product 8 in 99% yield.

We then turned our attention to compounds containing bioactive motifs (compounds 9–12) to demonstrate the applicability of this method to modify potential drug targets during structure activity relation (SAR) studies or to detoxify persistent potential environmental polluters found in water ways or soil.

Aliphatic iodide derivatives of naproxen, probenecid, and ibuprofen were successfully defunctionalized affording products 9, 10, and 11 in 90%, 99%, and 90% yields, respectively. A hydroxy group of glucose, one of the most abundant biomass-derived molecules,^11^ was successfully defunctionalized to generate the C(sp^3^)–H bond (12), offering a promising upcycling strategy for glucose-based derivatives.

Alkyl iodides generated from chloride and bromides were also defunctionalized, providing substrates 13 to 17 in yields ranging from 85% to 98%. These results highlight the selectivity of this reaction even in the presence of reduction-prone functional groups, such as phthalimides, esters, and furans. Notably, substrate 17, a vanillin-derived molecule was well-tolerated under the optimized conditions. Lastly, the reaction tolerated electronically deactivated aromatic C(sp^2^)–X bond, as demonstrated by the conversion of iodonaphthalene to naphthalene (18) in 60% yield.

**Scheme 4:**
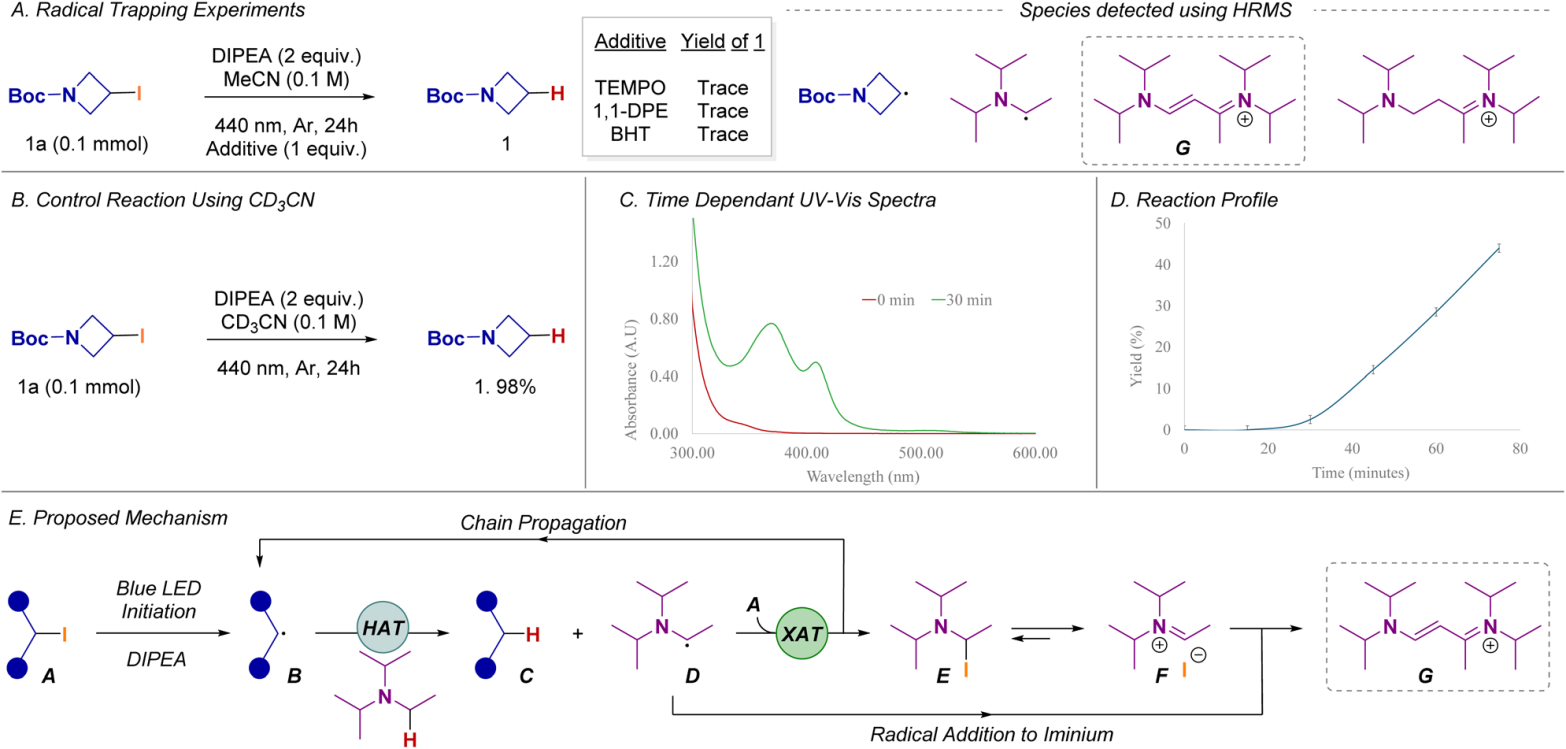
Mechanistic probes.

The mechanistic aspects of this transformation were explored starting with the radical trapping experiments (Scheme 4A). Signals corresponding to both alkyl radicals and α-amino alkyl radicals, originating from DIPEA were detected. Additionally, the formation of a streptocyanine dye (G) derived from DIPEA was observed, which may act as a potential photocatalyst, as reported by Weaver.^7*b*^ To investigate the source of the hydrogen atom, the reaction was performed in acetonitrile-d_3_ (CD_3_CN). The resulting defunctionalized product did not incorporate deuterium atoms, suggesting that the hydrogen-atom transfer (HAT) does not involve the solvent, but instead likely occurs with DIPEA (Scheme 4B).

UV-vis studies were conducted to challenge our initial hypothesis that the reaction proceeds *via* an electron-donor-acceptor (EDA) complex between alkyl iodide 1a and DIPEA (SI-47). The absence of a charge-transfer band upon mixing suggests a low likelihood of photoactive aggregate formation.^12^ Notably, comparison of the UV-vis spectra recorded at 0 and 30 min revealed a pronounced increase in absorbance, accompanied by two distinct peaks at 370 nm and 410 nm. These features suggest the *in situ* generation of a photoactive species during the reaction (Scheme 4C); presumably a streptocyanine dye (G). Finally, we explored the transformation kinetics to obtain a reaction profile (Scheme 4D), which shows that the reaction has an initiation period (∼30 min). However, at 45 min, once product is formed, the reaction accelerates and generates 45% product within the next 30 min.

Given the results above we surmised that the reaction could proceed *via* a radical chain propagation that involves a self-sustaining XAT process. To further support this hypothesis, we performed a quantum yield calculation. If the quantum yield is above 1, this would suggest that a radical chain propagation is at play.^13^ Our experimental results show that this transformation has a quantum yield of ∼20. This result, along-side the initiation period observed, strongly supports a self-sustaining dehalogenation pathway that simultaneously generates a photoactive species. Building on these probes and previous reports,^9^ we propose the following reaction pathway (Scheme 4E). In the presence of DIPEA and blue LED irradiation, the alkyl iodide A generates trace amounts of alkyl radicals B. These radicals perform a hydrogen-atom abstraction in the α-position of DIPEA, yielding the desired dehalogenated product C alongside an α-amino alkyl radical D, which is a well-studied XAT agent. Two plausible scenarios may arise from this intermediate: (i) the α-amino alkyl radical engages in a chain propagation step by reacting with an additional equivalent of alkyl iodide, and/or (ii) it facilitates the formation of a streptocyanine dye (G), which subsequently acts as a photocatalyst in the activation of alkyl iodides.^7*b*^

**Scheme 5:**
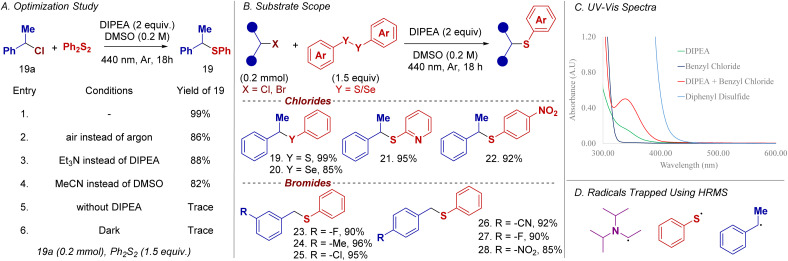
Chalcogenation.

The applicability of tertiary amines in other transformations that require more sustainable methods was further explored. Of particular interest, we developed a cross-electrophile coupling between benzyl halides and disulfides (Scheme 5).^14^ An extensive optimization study identified the optimal conditions (Scheme 5A). Control experiments revealed that both light and amine are essential for the reaction to proceed (See SI-8 for detailed optimization study).

A broad range of benzyl halides bearing both electron-deficient and electron-rich aromatic substituents were well-tolerated, affording the corresponding products (19 to 28) in excellent yields ranging from 80% to 99%. In contrast, dialkyl disulfides failed to produce the expected chalcogenated products under the optimized conditions (see SI-9 for unsuccessful substrates).

UV-vis spectroscopy investigations of this cross-electrophile coupling reaction revealed the formation of a charge-transfer band upon mixing DIPEA and benzyl halide, indicating the possible generation of an EDA complex (Scheme 5C), analogous to reported EDA complexes of anilines and aryl iodides.^15^ This result suggests a different mechanistic pathway when compared to the dehalogenation reaction. Complementary radical-trapping experiments analyzed by high-resolution mass-spectrometry (HRMS) confirmed the presence of an α-amino alkyl radical, a sulfur-centered radical, and a benzyl radical.

**Scheme 6:**
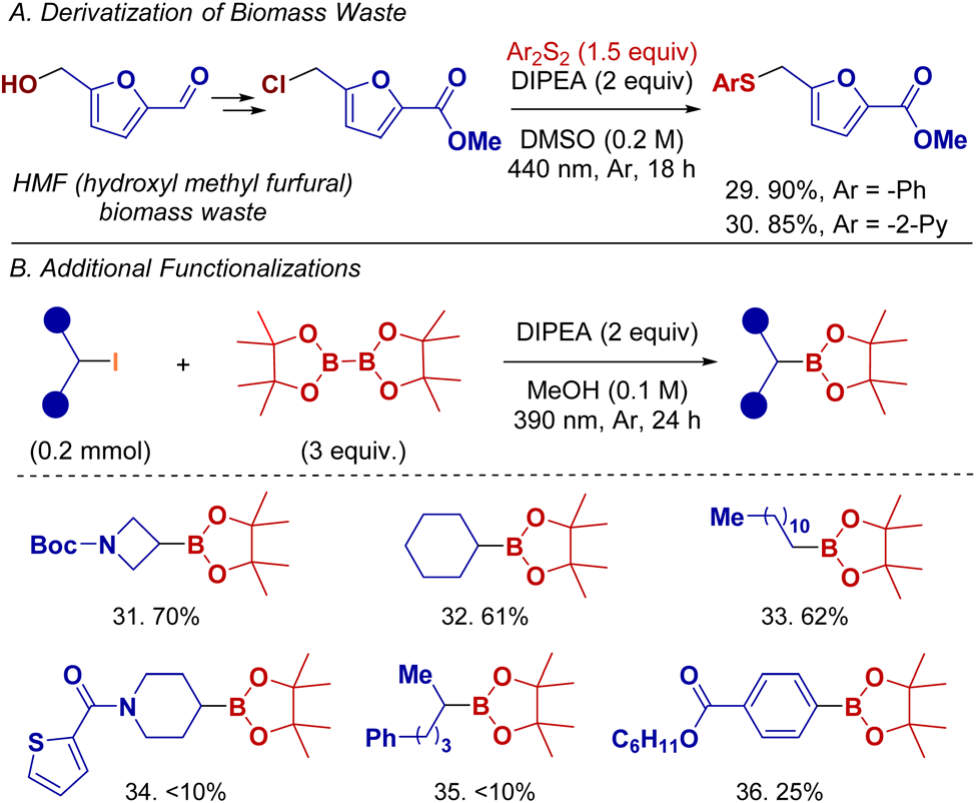
Derivatization and additional functionalization.

A possible application of this cross-electrophile coupling reaction manifests in the possible upcycling of hydroxymethylfurfural, a common biomass-derived waste for which efficient transformations into commodity chemicals remains limited (Scheme 6A).^16^ Chlorinated furfural was successfully chalcogenated into products 29 and 30 in excellent yields (90% and 85% respectively).

Lastly, the versatility of this Lewis base-promoted strategy was further expanded to borylation^17^ of a limited number of alkyl iodides (see SI-6 for detailed reaction conditions). Indeed, starting material 1a was successfully borylated into product 31 in 70% yield. While simple alkyl motifs such as iodocyclohexane and iodododecane afforded products 32 and 33 in moderate yields (61%, 62%, respectively), any other functionalized alkyl iodides did not provide the borylated products in synthetically useful yields (34 and 35), generating instead the dehalohydrogenated compounds. Similarly, aryl iodides did not borylated in good yields, but the remaining mass balance was unreacted starting materials.

## 3. Conclusion

We report a greener and versatile approach for the activation of structurally diverse organic halides. This protocol complements established photocatalytic methodologies by enabling selective dehalogenation or functionalization of structurally rich scaffolds. The method is scalable and uses readily available commodity chemicals. Continued investigations in our laboratory aim to broaden the substrate scope and elucidate key mechanistic features underlying this reactivity.

## Conflicts of interest

There are no conflicts to declare.

## Supplementary Material

RA-015-D5RA07627G-s001

## Data Availability

All data presented and generated in this manuscript is accessible in the supplementary information (SI). Supplementary information is available. See DOI: https://doi.org/10.1039/d5ra07627g.
