# Supplementary material for: Catalyst-free photoinduced dehalogenation and functionalization of alkyl halides using Lewis bases
Source: RSC Adv. 2025 Nov 5;15(50):43155–60. doi: 10.1039/d5ra07627g (PMC12588303; doi:10.1039/d5ra07627g)
Supplement: RA-015-D5RA07627G-s001 [file RA-015-D5RA07627G-s001.pdf]

Supplementary Information (SI) for RSC Advances.
This journal is © The Royal Society of Chemistry 2025

Supporting Information

Catalyst-Free Photoinduced Dehalogenation and Functionalization of Alkyl Halides Using Lewis Bases

Krishnakumar Sachidanandan, Anulika Umunnah, Alexis Terese Hahn and Sébastien Laulhé*

Indiana University Indianapolis,

Indianapolis, Indiana 46202, United States.

Corresponding Author Email: slaulhe@iu.edu

Table of Contents

1. General Information SI-3
2. General Procedures SI-4
3. Optimization Study SI-7
4. Unsuccessful Substrates SI-9
5. Experimental Details and Characterization Data for Products SI-10
6. Radical Trapping Study SI-45
7. Dehalogenation Reaction Using Acetonitrile-d_3 .... SI-46
8. UV-Vis Study SI-47
9. Reaction Profile SI-51
10. Photon-Flux Calculation SI-52
11. Quantum Yield Calculation SI-54
12. ^{1}H NMR, ^{19}F NMR, ^{13}C NMR Spectra SI-55
13. References SI-94

1. General Information

All reagents and solvents were purchased and used without further purification unless otherwise noted. All reactions were performed under an inert atmosphere unless otherwise stated. Room temperature refers to 26 °C, unless otherwise noted. Moisture-sensitive reactions were performed using flame-dried glassware under an atmosphere of dry argon (Ar). Air- and water sensitive reactions were setup in a Vacuum Atmosphere GENESIS glovebox held under an atmosphere of argon gas (working pressure 2−6 mbar). Flame-dried equipment was stored in a 130 °C oven before use and either allowed to cool in a cabinet desiccator or assembled hot and allowed to cool under an inert atmosphere. Chromatographic purification of products was accomplished using flash column chromatography Silicycle Silica flash F60 (particle size 40−63 μm, 230−400 mesh). Thin-layer chromatography was performed on EMD Millipore silica gel 60 F254 glass-backed plates (layer thickness 250 μm, particle size 10−12 μm, impregnated with a fluorescent indicator). Visualization of the developed chromatogram was accomplished by fluorescence quenching under shortwave UV light and/or by staining with phosphomolybdic acid, p-anisaldehyde, or $KMnO_4$ stains.

LED Lamps. The following Kessil LED lamps were used in this work:

- 390 nm lamp: PR160L-390, 40W (purple visible light)
- 427 nm lamp: PR160L-427, 40W (blue visible light)
- 440 nm lamp: PR160L-440, 40W (blue visible light)
- 525 nm lamp: PR160L-525, 40W (green visible light)

Reaction Vials. We used ChemGlass microwave reaction vials with heavy walls made of borosilicate glass (product # CG-4920-01). The vial was placed approximately 3 cm away from the LED lamps, with the LEDs shining directly at the side of the vial as shown in following picture. Three reactions per lamp could be set up at the same time. And a fan above the reaction vials can keep the temperature around 35 °C. 10 mL microwave reaction vial secured by 20 mm aluminum seals with 0.125-inch thick, blue PTFE / white silicone septa was used for the reaction.

Instrumentation. For NMR spectrometry, NMR spectra were obtained on Bruker spectrometers operating at 400 or 500 MHz for ^{1}H NMR and 101 or 126 MHz for ^{13}C{1 H} NMR. The data were reported in the following order: chemical shifts (δ ppm), multiplicity (s = singlet, d = doublet, dd = doublet of doublets, t = triplet, q = quartet, m = multiplet), coupling constant, (Hz), relative integral made in reference to NMR solvent signals. For mass spectrometry, gas chromatograph−mass spectrometry was obtained using a Agilent GC System Intuvo 9000 Series coupled with a Agilent 5977B GC/MSD Mass Selective Detector. High resolution mass spectra were obtained using a Thermo Orbitrap Fusion Tribid Mass Spectrometer with electrospray ionization (ESI).

2. General Procedures

2.1 Synthesis of alkyl halides were performed according to previous reports.[1]

Appel Reaction

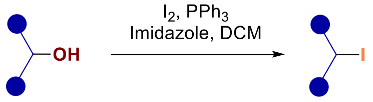

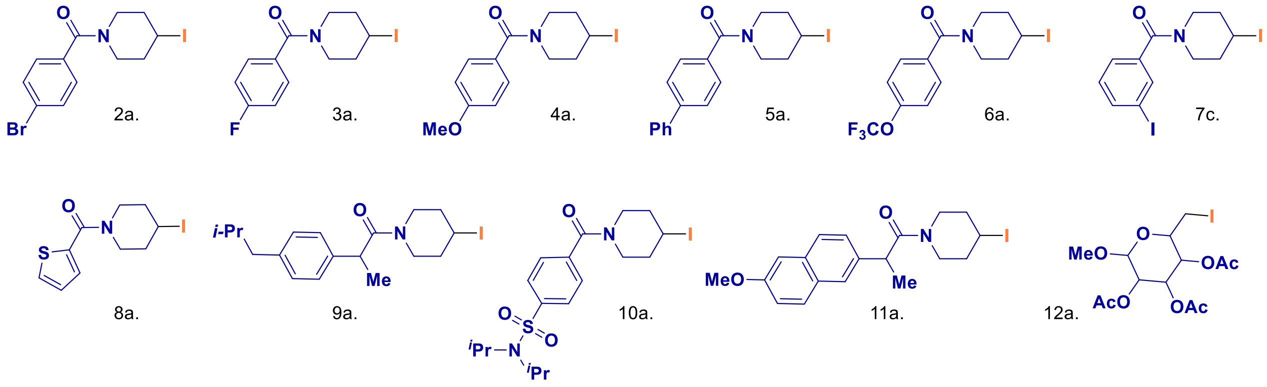

Finkelstein Reaction

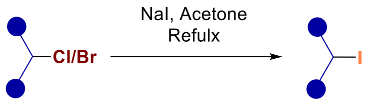

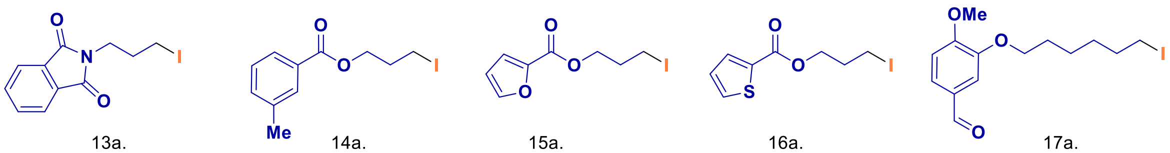

2.2 General procedure for the defunctionalization of alkyl iodides.

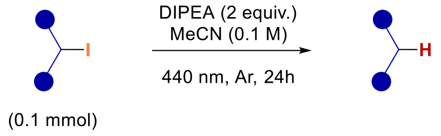

Alkyl iodide (0.1 mmol, 1 equiv.), DIPEA (35 μL, 0.2 mmol, 2 equiv.), and acetonitrile (1 mL) were mixed in a 10 mL microwave vial equipped with a stir bar under argon atmosphere. The vial was sealed with a septum-cap and placed 2 cm away from two 440 nm blue LED (40W). The temperature was kept at approximately 35 °C through cooling with a fan (heating caused by LED lamp). After being stirred for 24 hours, the reaction mixture was directly transferred into a scintillation vial, and concentrated under vacuum. The residue was further purified by flash column chromatography on silica gel.

2.3 General procedure for the chalcogenation of alkyl chlorides and bromides.

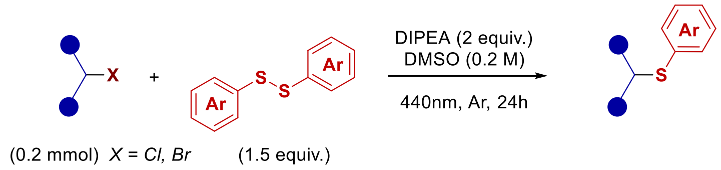

Alkyl halide (0.2 mmol, 1 equiv.), diaryl disulfides (0.3 mmol, 1.5 equiv.), DIPEA (70 μL, 0.4 mmol, 2 equiv.), and DMSO (1 mL) were mixed in a 10 mL microwave vial equipped with a stir bar under argon atmosphere. The vial was sealed with a septum-cap and placed 2 cm away from two 440 nm blue LED (40W). The temperature was kept at approximately 35 °C through cooling with a fan (heating caused by LED lamp). After being stirred for 24 hours, the reaction mixture was the reaction mixture was poured into 20 mL of water, and the resulting mixture was extracted with ethyl acetate (3 × 20 mL). The combined organic phase was dried over $NaSO_4$, filtered, and concentrated under vacuum. The residue was further purified by flash column chromatography on silica gel.

2.4 General procedure for the borylation of alkyl iodides.

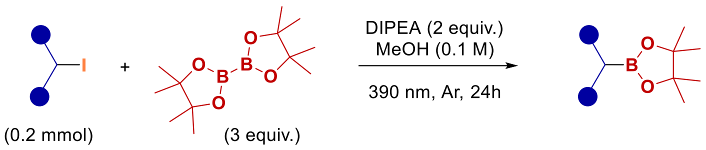

Alkyl iodide (0.2 mmol, 1 equiv.), bis(pinacolato)diboron (152.4 mg, 0.6 mmol, 3 equiv.), DIPEA (70 µL, 0.4 mmol, 2 equiv.), and methanol (2 mL) were mixed in a 10 mL microwave vial equipped with a stir bar under argon atmosphere. The vial was sealed with a septum-cap and placed 2 cm away from two 390 nm purple LED (40W). The temperature was kept at approximately 35 °C through cooling with a fan (heating caused by LED lamp). After being stirred for 24 hours, the reaction mixture was the reaction mixture was poured into 20 mL of water, and the resulting mixture was extracted with dichloromethane (3 × 20 mL). The combined organic phase was dried over Na_2SO_4, filtered, and concentrated under vacuum. The residue was further purified by flash column chromatography on silica gel.

3. Optimization Study:

3.1 Optimization for the defunctionalization of alkyl iodides.

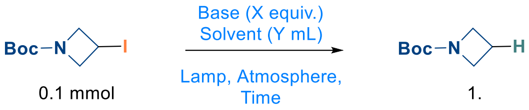

Table S1. ^{1}HNMR yields using 1,2-dibromoethane as internal standard.

Entry	Base (X equiv.)	Solvent (Y mL)	Lamp	Atmosphere	Time	Yield of 1
1.	DIPEA (1 equiv.)	MeCN (1 mL)	440 nm	Argon	17 h	78%
2.	Bu_3N (1 equiv.)	MeCN (1 mL)	440 nm	Argon	17 h	76%
3.	Et_3N (1 equiv.)	MeCN (1 mL)	440 nm	Argon	17 h	75%
4.	DABCO (1 equiv.)	MeCN (1 mL)	440 nm	Argon	17 h	Trace
5.	DBU (1 equiv.)	MeCN (1 mL)	440 nm	Argon	17 h	Trace
6.	Ph_3N (1 equiv.)	MeCN (1 mL)	440 nm	Argon	17 h	< 10%
7.	K_2CO_3 (1 equiv.)	MeCN (1 mL)	440 nm	Argon	17 h	Trace
8.	No Base	MeCN (1 mL)	440 nm	Argon	17 h	Trace
9.	DIPEA (1 equiv.)	MeCN (1 mL)	427 nm	Argon	17 h	70%
10.	DIPEA (1 equiv.)	MeCN (1 mL)	390 nm	Argon	17 h	73%
11.	DIPEA (1 equiv.)	MeCN (1 mL)	Dark	Argon	17 h	N/R
12.	DIPEA (1 equiv.)	DMSO (1 mL)	440 nm	Argon	17 h	67%
13.	DIPEA (1 equiv.)	MeOH (1 mL)	440 nm	Argon	17 h	75%
14.	DIPEA (1 equiv.)	H_2O (1 mL)	440 nm	Argon	17 h	32%
15.	DIPEA (1 equiv.)	MeCN (1 mL)	440 nm	Argon	24 h	86%
16.	DIPEA (2 equiv.)	MeCN (1 mL)	440 nm	Argon	24 h	99%
17.	DIPEA (2 equiv.)	MeCN (2 mL)	440 nm	Argon	24 h	91%
18.	DIPEA (2 equiv.)	MeCN (1 mL)	440 nm	Air	24 h	90%

3.2 Optimization for the chalcogenation of benzyl halides.

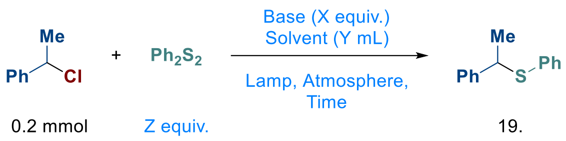

Table S2. ^{1}HNMR yields using 1,2-dibromoethane as internal standard.

Entry	Base (X equiv.)	Solvent (Y mL)	Lamp	Ph_2S_2 (equiv.)	Atmosphere	Time	Yield of 19
1.	Et_3N (2 equiv)	DMSO (1 mL)	440 nm	1.5 equiv.	Argon	18 h	88%
2.	DIPEA (2 equiv)	DMSO (1 mL)	440 nm	1.5 equiv.	Argon	18 h	99%
3.	Ph_3N (2 equiv)	DMSO (1 mL)	440 nm	1.5 equiv.	Argon	18 h	< 10%
4.	DBU (2 equiv)	DMSO (1 mL)	440 nm	1.5 equiv.	Argon	18 h	80%
5.	DABCO (2 equiv)	DMSO (1 mL)	440 nm	1.5 equiv.	Argon	18 h	< 10%
6.	KO^{t}Bu (2 equiv)	DMSO (1 mL)	440 nm	1.5 equiv.	Argon	18 h	30%
7.	K_2CO_3 (2 equiv)	DMSO (1 mL)	440 nm	1.5 equiv.	Argon	18 h	60%
8.	No Base	DMSO (1 mL)	440 nm	1.5 equiv.	Argon	18 h	Trace
9.	DIPEA (2 equiv)	MeCN (1 mL)	440 nm	1.5 equiv.	Argon	18 h	82%
10.	DIPEA (2 equiv)	THF (1 mL)	440 nm	1.5 equiv.	Argon	18 h	34%
11.	DIPEA (2 equiv)	DMF (1 mL)	440 nm	1.5 equiv.	Argon	18 h	8%
12.	DIPEA (2 equiv)	MeOH (1 mL)	440 nm	1.5 equiv.	Argon	18 h	80%
13.	DIPEA (2 equiv)	Acetone (1 mL)	440 nm	1.5 equiv.	Argon	18 h	33%
15.	DIPEA (2 equiv)	DMSO (1 mL)	525 nm	1.5 equiv.	Argon	18 h	17%
17.	DIPEA (2 equiv)	DMSO (1 mL)	427 nm	1.5 equiv.	Argon	18 h	90%
18.	DIPEA (2 equiv)	DMSO (1 mL)	390 nm	1.5 equiv.	Argon	18 h	58%
19.	DIPEA (2 equiv)	DMSO (1 mL)	CFL	1.5 equiv.	Argon	18 h	49%
20.	DIPEA (2 equiv)	DMSO (1 mL)	Dark	1.5 equiv.	Argon	18 h	N/R
21.	DIPEA (2 equiv)	DMSO (1 mL)	440 nm	1.5 equiv.	Air	18 h	86%
22.	DIPEA (2 equiv)	DMSO (1 mL)	440 nm	1.5 equiv.	Nitrogen	18 h	84%
23.	DIPEA (2 equiv)	DMSO (1 mL)	440 nm	1.1 equiv.	Argon	18 h	92%

24.	DIPEA (2 equiv)	DMSO (1 mL)	440 nm	1.3 equiv.	Argon	18 h	97%
25.	DIPEA (1 equiv)	DMSO (1 mL)	440 nm	1.5 equiv.	Argon	18 h	67%
26.	DIPEA (1.5 equiv)	DMSO (1 mL)	440 nm	1.5 equiv.	Argon	18 h	81%

4. Unsuccessful Substrates:

4.1. De-functionalization:

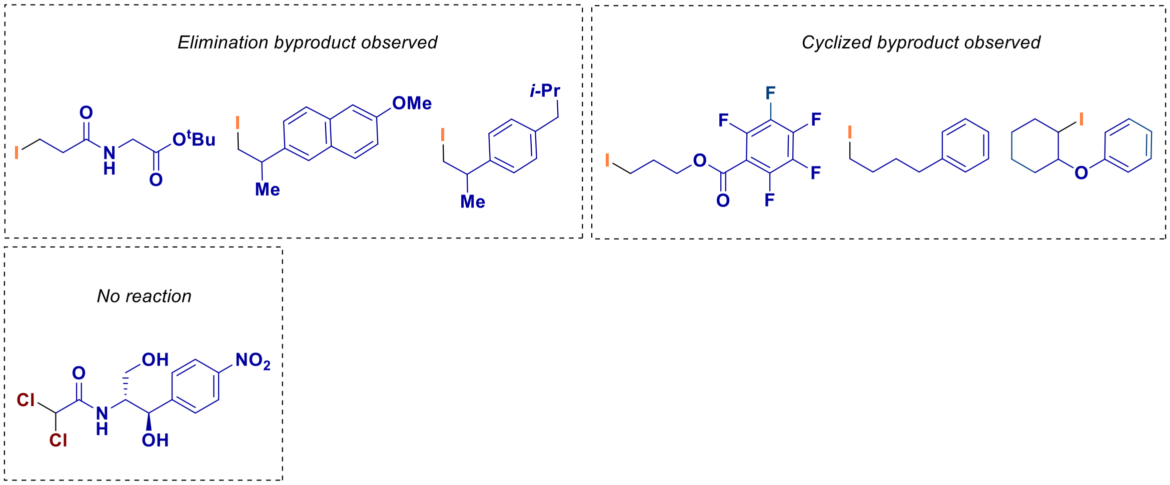

4.2. Chalcogenation:

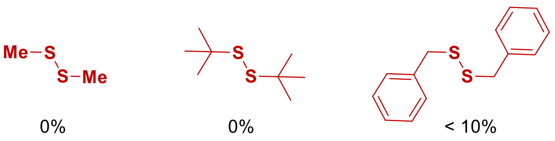

4.3. Borylation:

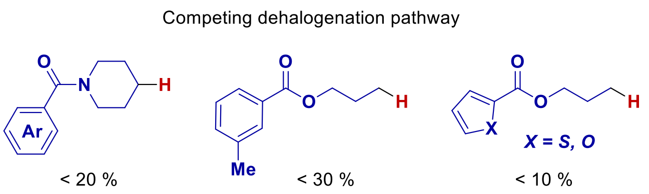

5. Experimental Details and Characterization Data for Products:

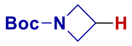

1.

Known compound reported by reference.[2]

***tert*-Butyl azetidine-1-carboxylate (1):** Synthesized from tert-butyl 3-iodoazetidine-1-carboxylate (**1a**) (17.5 µL, 0.1 mmol, 1 equiv.) according to **General Procedure 2.2**. The residue was purified by flash column, eluting with ethyl acetate/hexane (1:5) to afford the desired compound as a colorless oil (15.5 mg, 99% yield).

^{1}H NMR (400 MHz, $CDCl_3$) δ 4.04 – 3.83 (m, 4H), 2.26 – 2.07 (m, 2H), 1.42 (s, 9H).
^{13}C NMR (101 MHz, $CDCl_3$) δ 156.4, 79.1, 49.2, 28.4, 15.3.

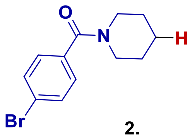

2.

Known compound reported by reference.[3]

(4-bromophenyl)(piperidin-1-yl)methanone (2): Synthesized from (4-bromophenyl)(4-iodopiperidin-1-yl)methanone (**2a**) (39.4 mg, 0.1 mmol, 1 equiv.) according to **General Procedure 2.2**. The residue was purified by flash column, eluting with ethyl acetate/hexane (1:2) to afford the desired compound as a white solid (26.3 mg, 98% yield).

^{1}H NMR (400 MHz, $CDCl_3$) δ 7.41 (d, J = 8.4 Hz, 2H), 7.14 (d, J = 8.4 Hz, 2H), 3.56 (s, 2H), 3.20 (s, 2H), 1.69 – 1.31 (m, 6H).

^{13}C NMR (101 MHz, $CDCl_3$) δ 169.2, 135.3, 131.6, 128.6, 123.6, 48.8, 43.2, 26.5, 25.6, 24.5.

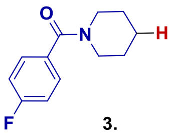

3.

Known compound reported by reference.[3]

(4-fluorophenyl)(piperidin-1-yl)methanone (3): Synthesized from tert-butyl (4-fluorophenyl)(4-iodopiperidin-1-yl)methanone (**3a**) (33.3 mg, 0.1 mmol, 1 equiv.) according to **General Procedure 2.2**. The residue was purified by flash column, eluting with ethyl acetate/hexane (1:2) to afford the desired compound as a colorless oil (20.5 mg, 98% yield).

^{1}H NMR (400 MHz, $CDCl_3$) δ 7.45 – 7.34 (m, 2H), 7.13 – 7.01 (m, 2H), 3.67 (s, 2H), 3.34 (s, 2H), 1.78 – 1.40 (m, 6H).

^{13}C NMR (101 MHz, $CDCl_3$) δ 169.4, 163.22 (d, J = 249.4 Hz), 132.50 (d, J = 3.7 Hz), 129.09 (d, J = 8.4 Hz), 115.44 (d, J = 21.6 Hz), 48.9, 43.3, 26.4, 25.6, 24.6.

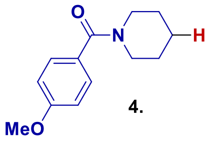

4.

Known compound reported by reference.[3]

(4-methoxyphenyl)(piperidin-1-yl)methanone (4): Synthesized from (4-iodopiperidin-1-yl)(4-methoxyphenyl)methanone (**4a**) (34.5 mg, 0.1 mmol, 1 equiv.) according to **General Procedure 2.2**. The residue was purified by flash column, eluting with ethyl acetate/hexane (1:2) to afford the desired compound as a white solid (20.8 mg, 95% yield).

^{1}H NMR (400 MHz, $CDCl_3$) δ 7.35 (d, J = 8.8 Hz, 2H), 6.88 (d, J = 8.7 Hz, 2H), 3.81 (s, 3H), 3.73 – 3.26 (m, 4H), 1.73 – 1.43 (m, 6H).

^{13}C NMR (101 MHz, $CDCl_3$) δ 170.3, 160.5, 128.8, 128.6, 113.6, 55.3, 48.8, 43.3, 26.1, 24.7.

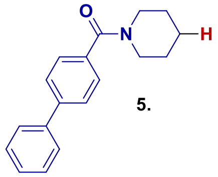

5.

Known compound reported by reference.[3]

[1,1'-biphenyl]-4-yl(piperidin-1-yl)methanone (5): Synthesized from [1,1'-biphenyl]-4-yl(4-iodopiperidin-1-yl)methanone (**5a**) (39.1 mg, 0.1 mmol, 1 equiv.) according to **General Procedure 2.2**. The residue was purified by flash column, eluting with ethyl acetate/hexane (1:5) to afford the desired compound as a light brown solid (25.9 mg, 98% yield).

^{1}H NMR (400 MHz, $CDCl_3$) δ 7.67 – 7.54 (m, 4H), 7.52 – 7.40 (m, 4H), 7.40 – 7.31 (m, 1H), 3.73 (s, 2H), 3.41 (s, 2H), 1.79 – 1.43 (m, 6H).

^{13}C NMR (101 MHz, $CDCl_3$) δ 170.1, 142.3, 140.4, 135.3, 128.9, 127.7, 127.4, 127.1, 127.1, 48.9, 43.3, 26.6, 25.7, 24.7.

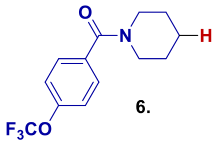

6.

Known compound reported by reference.[4]

piperidin-1-yl(4-(trifluoromethoxy)phenyl)methanone (6): Synthesized from tert-butyl 3-iodoazetidine-1-carboxylate (**1A**) (39.9 mg, 0.1 mmol, 1 equiv.) according to **General Procedure 2.2**. The residue was purified by flash column, eluting with ethyl acetate/hexane (1:5) to afford the desired compound as a colorless oil (26.7 mg, 99% yield).

^{1}H NMR (400 MHz, $CDCl_3$) δ 7.43 (d, 2H), 7.23 (d, J = 8.4 Hz, 2H), 3.69 (s, 2H), 3.33 (s, 2H), 1.77 – 1.44 (m, 6H).

^{13}C NMR (101 MHz, $CDCl_3$) δ 168.9, 149.81 (q, J = 2.0 Hz), 135.1, 128.6, 120.9, 120.4 (q, J = 258.2 Hz), 48.8, 43.3, 26.5, 25.6, 24.5.

^{19}F NMR (376 MHz, $CDCl_3$) δ -57.80.

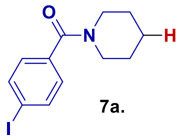

7a.

Known compound reported by reference.[5]

(4-iodophenyl)(piperidin-1-yl)methanone (7a): Synthesized from (4-iodophenyl)(4-iodopiperidin-1-yl)methanone (**7c**) (44.1 mg, 0.1 mmol, 1 equiv.) according to **General Procedure 2.2**. The residue was purified by flash column, eluting with ethyl acetate/hexane (1:5) to afford the desired compound as a white solid (18.9 mg, 60% yield).

^{1}H NMR (400 MHz, $CDCl_3$) δ 7.39 (s, 5H), 3.71 (s, 2H), 3.34 (s, 2H), 1.76 – 1.45 (m, 8H).

^{13}C NMR (101 MHz, $CDCl_3$) δ 170.3, 136.6, 129.3, 128.4, 126.8, 48.8, 43.1, 29.7, 26.5, 25.7, 24.6.

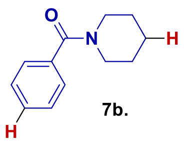

7b.

Known compound reported by reference.[5]

Phenyl(piperidin-1-yl)methanone (**7b**): Brown powder (3.0 mg, 16% yield).

^{1}H NMR (400 MHz, $CDCl_3$) δ 7.78 – 7.68 (m, 2H), 7.37 – 7.31 (m, 1H), 7.17 – 7.07 (m, 1H), 3.68 (s, 2H), 3.31 (s, 2H), 1.77 – 1.44 (m, 6H).

^{13}C NMR (101 MHz, $CDCl_3$) δ 168.4, 138.5, 138.3, 135.6, 130.1, 125.9, 94.9, 48.8, 43.2, 29.7, 26.5, 25.6, 24.5.

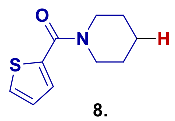

8.

Known compound reported by reference.[6]

Piperidin-1-yl(thiophen-2-yl)methanone (8): Synthesized from (4-iodopiperidin-1-yl)(thiophen-2-yl)methanone (**8a**) (32.1 mg, 0.1 mmol, 1 equiv.) according to **General Procedure 2.2**. The residue was purified by flash column, eluting with ethyl acetate/hexane (1:10) to afford the desired compound as a white solid (19.3 mg, 99% yield).

^{1}H NMR (400 MHz, $CDCl_3$) δ 7.48 – 7.36 (m, 1H), 7.30 – 7.17 (m, 1H), 7.10 – 6.96 (m, 1H), 3.73 – 3.59 (m, 4H), 1.76 – 1.57 (m, 6H).

^{13}C NMR (101 MHz, $CDCl_3$) δ 163.5, 137.7, 128.3, 128.1, 126.5, 26.1, 24.6.

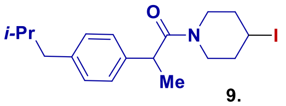

9.

Known compound reported by reference.[7]

2-(4-isobutylphenyl)-1-(piperidin-1-yl)propan-1-one (9): Synthesized from 1-(4-iodopiperidin-1-yl)-2-(4-isobutylphenyl)propan-1-one (**9a**) (39.9 mg, 0.1 mmol, 1 equiv.) according to **General Procedure 2.2**. The residue was purified by flash column, eluting with ethyl acetate/hexane (1:5) to afford the desired compound as a white solid (24.6 mg, 90% yield).

^{1}H NMR (400 MHz, $CDCl_3$) δ 7.16 – 7.10 (m, 2H), 7.09 – 7.03 (m, 2H), 3.88 – 3.80 (m, 1H), 3.79 – 3.70 (m, 1H), 3.43 – 3.18 (m, 3H), 2.42 (d, J = 7.2 Hz, 2H), 1.90 – 1.75 (m, 1H), 1.55 – 1.46 (m, 3H), 1.41 (d, J = 6.8 Hz, 4H), 1.36 – 1.26 (m, 1H), 1.01 – 0.91 (m, 1H), 0.87 (d, J = 6.6 Hz, 6H).

^{13}C NMR (101 MHz, $CDCl_3$) δ 171.9, 139.9, 139.6, 129.5, 126.9, 46.5, 45.0, 43.1, 42.8, 30.2, 25.8, 25.5, 24.5, 22.3, 20.8.

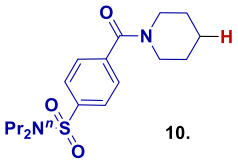

10.

Known compound reported by reference.[8]

4-(piperidine-1-carbonyl)-*N*,*N*-dipropylbenzenesulfonamide (10): Synthesized from 4-(4-iodopiperidine-1-carbonyl)-*N*,*N*-dipropylbenzenesulfonamide (**10a**) (47.8 mg, 0.1 mmol, 1 equiv.) according to **General Procedure 2.2**. The residue was purified by flash column, eluting with ethyl acetate/hexane (1:10) to afford the desired compound as a white solid (34.8 mg, 99% yield).

^{1}H NMR (400 MHz, $CDCl_3$) δ 7.90 – 7.78 (m, 1H), 7.57 – 7.44 (m, 1H), 3.71 (s, 1H), 3.27 (s, 1H), 3.14 – 3.02 (m, 2H), 1.76 – 1.63 (m, 2H), 1.61 – 1.45 (m, 3H), 0.87 (t, J = 7.4 Hz, 3H).

^{13}C NMR (101 MHz, $CDCl_3$) δ 168.6, 140.9, 140.3, 127.3, 127.3, 50.2, 48.7, 43.2, 26.5, 25.6, 24.4, 22.1, 11.2.

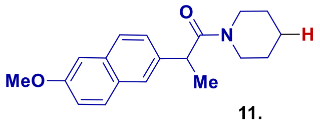

11.

Known compound reported by reference.[7]

2-(6-methoxynaphthalen-2-yl)-1-(piperidin-1-yl)propan-1-one (11): Synthesized from 1-(4-iodopiperidin-1-yl)-2-(6-methoxynaphthalen-2-yl)propan-1-one (**11a**) (42.3 mg, 0.1 mmol, 1 equiv.) according to **General Procedure 2.2**. The residue was purified by flash column, eluting with ethyl acetate/hexane (1:5) to afford the desired compound as a white solid (26.7 mg, 90% yield).

^{1}H NMR (400 MHz, $CDCl_3$) δ 7.69 (dd, J = 8.6, 4.8 Hz, 2H), 7.62 – 7.58 (m, 1H), 7.36 (dd, J = 8.4, 2.0 Hz, 1H), 7.16 – 7.09 (m, 2H), 4.01 (q, J = 6.8 Hz, 1H), 3.91 (s, 3H), 3.78 – 3.66 (m, 1H), 3.51 – 3.41 (m, 1H), 3.37 – 3.27 (m, 2H), 1.58 – 1.37 (m, 7H), 1.35 – 1.23 (m, 1H), 1.01 – 0.91 (m, 1H).

^{13}C NMR (101 MHz, $CDCl_3$) δ 171.9, 157.5, 137.6, 133.4, 129.2, 129.1, 127.4, 126.2, 125.5, 118.9, 105.6, 55.3, 46.6, 43.1, 26.0, 25.6, 24.5, 20.9.

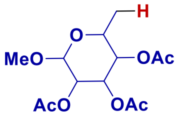

12.

Known compound reported by reference.[9]

2-methoxy-6-methyltetrahydro-2H-pyran-3,4,5-triyl triacetate (12): Synthesized from 2-(iodomethyl)-6-methoxytetrahydro-2H-pyran-3,4,5-triyl triacetate (**12a**) (43.0 mg, 0.1 mmol, 1 equiv.) according to **General Procedure 2.2**. The residue was purified by flash column, eluting with ethyl acetate/hexane (1:5) to afford the desired compound as a white solid (30.1 mg, 99% yield).

^{1}H NMR (400 MHz, $CDCl_3$) δ 5.47 – 5.35 (m, 1H), 4.88 – 4.81 (m, 2H), 4.77 (t, J = 9.7 Hz, 1H), 3.93 – 3.79 (m, 1H), 3.37 (s, 3H), 2.05 (s, 3H), 2.02 (s, 3H), 1.98 (s, 3H), 1.17 (d, J = 6.2 Hz, 3H).

^{13}C NMR (101 MHz, $CDCl_3$) δ 170.2, 170.1, 169.8, 96.6, 73.8, 71.2, 70.1, 64.9, 55.2, 20.7, 20.7, 17.2.

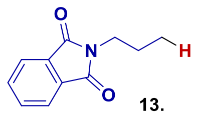

13.

Known compound reported by reference.[10]

2-propylisoindoline-1,3-dione (13): Synthesized from 2-(3-iodopropyl)isoindoline-1,3-dione (**13a**) (31.5, 0.1 mmol, 1 equiv.) according to **General Procedure 2.2**. The residue was purified by flash column, eluting with ethyl acetate/hexane (1:10) to afford the desired compound as a white solid (16.1 mg, 85% yield).

^{1}H NMR (400 MHz, $CDCl_3$) δ 7.87 – 7.76 (m, 2H), 7.70 (dd, J = 5.5, 3.1 Hz, 2H), 3.69 – 3.58 (m, 2H), 1.76 – 1.62 (m, 2H), 0.94 (t, J = 7.4 Hz, 3H).

^{13}C NMR (101 MHz, $CDCl_3$) δ 168.5, 133.8, 132.2, 123.1, 39.6, 21.9, 11.3.

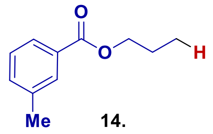

14.

Known compound reported by reference.[11]

propyl 3-methylbenzoate (14): Synthesized from 3-iodopropyl 3-methylbenzoate (**14a**) (30.4 mg, 0.1 mmol, 1 equiv.) according to **General Procedure 2.2**. The residue was purified by flash column, eluting with ethyl acetate/hexane (1:10) to afford the desired compound as a colorless oil (17.4 mg, 98% yield).

^{1}H NMR (400 MHz, $CDCl_3$) δ 7.89 – 7.82 (m, 2H), 7.39 – 7.29 (m, 2H), 4.28 (t, J = 6.7 Hz, 2H), 2.41 (s, 3H), 1.86 – 1.73 (m, 2H), 1.03 (t, J = 7.4 Hz, 3H).

^{13}C NMR (101 MHz, $CDCl_3$) δ 166.9, 138.1, 133.5, 130.5, 130.0, 128.2, 126.7, 66.5, 22.1, 21.3, 10.5.

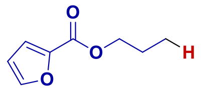

15.

Known compound reported by reference.[12]

propyl furan-2-carboxylate (15): Synthesized from 3-iodopropyl furan-2-carboxylate (**15a**) (28.0 mg, 0.1 mmol, 1 equiv.) according to **General Procedure 2.2**. The residue was purified by flash column, eluting with ethyl acetate/hexane (1:5) to afford the desired compound as a colorless oil (15.1 mg, 98% yield).

^{1}H NMR (400 MHz, $CDCl_3$) δ 7.61 – 7.53 (m, 1H), 7.17 (d, J = 3.5 Hz, 1H), 6.54 – 6.45 (m, 1H), 4.26 (t, J = 6.8 Hz, 2H), 1.83 – 1.71 (m, 2H), 1.00 (t, J = 7.5 Hz, 3H).

^{13}C NMR (101 MHz, $CDCl_3$) δ 158.9, 146.2, 144.9, 117.7, 111.8, 66.5, 22.1, 10.4.

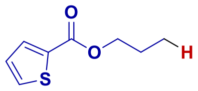

16.

Known compound reported by reference.[13]

propyl thiophene-2-carboxylate (16): Synthesized from 3-iodopropyl thiophene-2-carboxylate (**16a**) (29.6 mg, 0.1 mmol, 1 equiv.) according to **General Procedure 2.2**. The residue was purified by flash column, eluting with ethyl acetate/hexane (1:10) to afford the desired compound as a brown oil (16.7 mg, 98% yield).

^{1}H NMR (400 MHz, $CDCl_3$) δ 7.83 – 7.77 (m, 1H), 7.58 – 7.52 (m, 1H), 7.14 – 7.05 (m, 1H), 4.26 (t, J = 6.7 Hz, 2H), 1.85 – 1.71 (m, 2H), 1.02 (t, J = 7.5 Hz, 3H).

^{13}C NMR (101 MHz, $CDCl_3$) δ 162.36, 134.13, 133.2, 132.1, 127.7, 66.7, 22.1, 10.4.

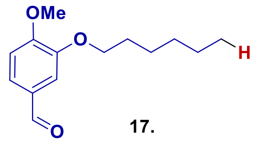

17.

3-(hexyloxy)-4-methoxybenzaldehyde (17): Synthesized from 3-((6-iodohexyl)oxy)-4-methoxybenzaldehyde (**17a**) (36.2 mg, 0.1 mmol, 1 equiv.) according to **General Procedure 2.2**. The residue was purified by flash column, eluting with ethyl acetate/hexane (1:10) to afford the desired compound as a colorless oil (23.1 mg, 98% yield).

^{1}H NMR (400 MHz, $CDCl_3$) δ 9.84 (s, 1H), 7.52 – 7.33 (m, 2H), 6.96 (d, J = 8.1 Hz, 1H), 4.10 (t, J = 6.8 Hz, 2H), 3.93 (s, 3H), 1.93 – 1.82 (m, 2H), 1.53 – 1.40 (m, 2H), 1.39 – 1.28 (m, 4H), 0.96 – 0.84 (m, 3H).

^{13}C NMR (101 MHz, $CDCl_3$) δ 190.9, 154.2, 149.9, 129.9, 126.8, 111.4, 109.3, 69.2, 56.0, 31.5, 28.9, 25.6, 22.6, 14.0.

HRMS (ESI-TOF) m/z: $[M + H]^+$ calcd. for $C_{14}H_{21}O_3$ 237.1491, found 237.1495.

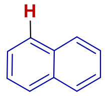

18.

Known compound reported by reference.[14]

Naphthalene (18): Synthesized from 1-iodonaphthalene (**18a**) (25.4 mg, 0.1 mmol, 1 equiv.) according to **General Procedure 2.2**. The residue was purified by flash column, eluting with hexane (100%) to afford the desired compound as a white solid (7.7 mg, 60% yield).

^{1}H NMR (400 MHz, $CDCl_3$) δ 7.90 – 7.79 (m, 4H), 7.54 – 7.42 (m, 4H).

^{13}C NMR (101 MHz, $CDCl_3$) δ 133.5, 127.9, 125.8.

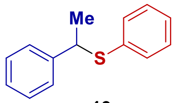

19.

Known compound reported by reference.[15]

Phenyl(1-phenylethyl)sulfane (19): Synthesized from 1-chloroethyl-benzene (26.4 μL, 0.2 mmol, 1 equiv.), and diphenyl disulfide (65.4 mg, 0.3 mmol, 1.5 equiv.) according to **General Procedure 2.4**. The residue was purified by flash column, eluting with hexane (100%) to afford the desired compound as a colorless oil (42.4 mg, 99% yield).

^{1}H NMR (400 MHz, $CDCl_3$) δ 7.35 – 7.26 (m, 6H), 7.25 – 7.17 (m, 4H), 4.35 (q, J = 7.0 Hz, 1H), 1.64 (d, J = 7.0 Hz, 3H).

^{13}C NMR (101 MHz, $CDCl_3$) δ 143.2, 135.1, 132.5, 128.7, 128.4, 127.3, 127.1, 48.0, 22.3.

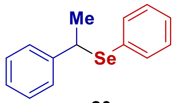

20.

Known compound reported by reference.[25]

Phenyl(1-phenylethyl)selane (20): Synthesized from 1-chloroethyl-benzene (26.4 µL, 0.2 mmol, 1 equiv.), and 1,2-diphenyl diselane (96.3 mg, 0.3 mmol, 1.5 equiv.) according to **General Procedure 2.4**. The residue was purified by flash column, eluting with hexane (100%) to afford the desired compound as a colorless oil (44.3 mg, 85% yield).

^{1}H NMR (400 MHz, $CDCl_3$) δ 7.46 – 7.38 (m, 2H), 7.29 – 7.14 (m, 8H), 4.44 (q, J = 7.0 Hz, 1H), 1.74 (d, J = 7.1 Hz, 3H).

^{13}C NMR (101 MHz, $CDCl_3$) δ 143.6, 135.5, 129.9, 128.8, 128.3, 127.8, 127.2, 126.9, 42.5, 22.2.

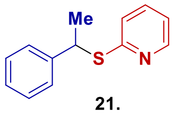

21.

Known compound reported by reference.[16]

2-((1-phenylethyl)thio)pyridine (21): Synthesized from 1-chloroethyl-benzene (26.4 μL, 0.2 mmol, 1 equiv.), and 2,2′-dipyridyl disulfide (66 mg, 0.3 mmol, 1.5 equiv.) according to **General Procedure 2.4**. The residue was purified by flash column, eluting with ethyl acetate/hexane (1:20) to afford the desired compound as a colorless oil (40.9 mg, 95% yield).

^{1}H NMR (400 MHz, $CDCl_3$) δ 8.52 – 8.39 (m, 1H), 7.49 – 7.40 (m, 3H), 7.34 – 7.27 (m, 2H), 7.25 – 7.19 (m, 1H), 7.15 – 7.08 (m, 1H), 7.01 – 6.93 (m, 1H), 5.11 (q, J = 7.1 Hz, 1H), 1.75 (d, J = 7.1 Hz, 3H).

^{13}C NMR (101 MHz, $CDCl_3$) δ 158.9, 149.4, 143.3, 136.0, 128.5, 127.4, 127.1, 122.9, 119.7, 43.6, 22.6.

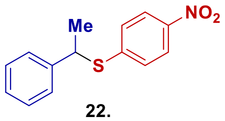

22.

Known compound reported by reference.[15]

(4-nitrophenyl)(1-phenylethyl)sulfane (22): Synthesized from 1-chloroethyl-benzene (26.4 µL, 0.2 mmol, 1 equiv.), and bis(4-nitrophenyl) disulfide (92.4 mg, 0.3 mmol, 1.5 equiv.) according to **General Procedure 2.4**. The residue was purified by flash column, eluting with ethyl acetate/hexane (1:6) to afford the desired compound as a colorless oil (47.6 mg, 92% yield).

^{1}H NMR (400 MHz, $CDCl_3$) δ 8.10 – 8.01 (m, 2H), 7.44 – 7.38 (m, 2H), 7.36 – 7.25 (m, 5H), 4.57 (q, J = 7.0 Hz, 1H), 1.71 (d, J = 7.0 Hz, 3H).

^{13}C NMR (101 MHz, $CDCl_3$) δ 146.3, 145.5, 142.1, 128.8, 128.5, 127.7, 127.1, 123.8, 46.5, 22.9.

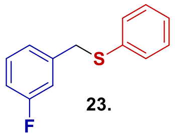

23.

Known compound reported by reference.[17]

(3-fluorobenzyl)(phenyl)sulfane (23): Synthesized from 3-fluorobenzyl bromide (24.5 µL, 0.2 mmol, 1 equiv.), and diphenyl disulfide (65.4 mg, 0.3 mmol, 1.5 equiv.) according to **General Procedure 2.4**. The residue was purified by flash column, eluting with hexane (100%) to afford the desired compound as a white solid (39.2 mg, 90% yield).

^{1}H NMR (400 MHz, $CDCl_3$) δ 7.33 – 7.14 (m, 6H), 7.06 – 6.95 (m, 2H), 6.95 – 6.86 (m, 1H), 4.07 (s, 2H).

^{13}C NMR (101 MHz, $CDCl_3$) δ 162.8 (d, J = 246.1 Hz), 140.2 (d, J = 7.7 Hz), 135.7, 130.2, 129.9 (d, J = 8.1 Hz), 128.9, 126.7, 124.5 (d, J = 2.9 Hz), 115.7 (d, J = 22.0 Hz), 114.1 (d, J = 20.9 Hz), 38.8 (d, J = 2.2 Hz).

^{19}F NMR (376 MHz, $CDCl_3$) δ -113.0.

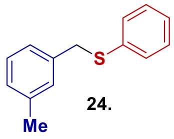

24.

Known compound reported by reference.[17]

(3-methylbenzyl)(phenyl)sulfane (24): Synthesized from 3-methylbenzyl bromide (27.0 µL, 0.2 mmol, 1 equiv.), and diphenyl disulfide (65.4 mg, 0.3 mmol, 1.5 equiv.) according to **General Procedure 2.4**. The residue was purified by flash column, eluting with hexane (100%) to afford the desired compound as a colorless oil (41.1 mg, 96% yield).

^{1}H NMR (400 MHz, $CDCl_3$) δ 7.34 – 7.29 (m, 2H), 7.29 – 7.21 (m, 2H), 7.21 – 7.14 (m, 2H), 7.13 – 7.07 (m, 2H), 7.07 – 7.02 (m, 1H), 4.09 (s, 2H), 2.32 (s, 3H).

^{13}C NMR (101 MHz, $CDCl_3$) δ 138.6, 137.3, 136.6, 129.7, 129.6, 128.8, 128.4, 127.9, 126.3, 125.9, 39.0, 21.3.

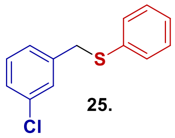

25.

Known compound reported by reference.[18]

(3-chlorobenzyl)(phenyl)sulfane (25): Synthesized from 3-chlorobenzyl bromide (26.2 μL, 0.2 mmol, 1 equiv.), and diphenyl disulfide (65.4 mg, 0.3 mmol, 1.5 equiv.) according to **General Procedure 2.4**. The residue was purified by flash column, eluting with ethyl acetate/hexane (1:10) to afford the desired compound as a yellow oil (44.5 mg, 95% yield).

^{1}H NMR (400 MHz, $CDCl_3$) δ 7.32 – 7.17 (m, 9H), 4.06 (s, 2H).

^{13}C NMR (101 MHz, $CDCl_3$) δ 136.2, 135.7, 132.9, 130.3, 130.1, 128.9, 128.6, 126.7, 38.6.

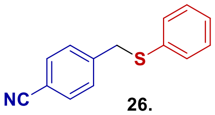

26.

Known compound reported by reference.[19]

4-((phenylthio)methyl)benzonitrile (26): Synthesized from 4-(bromomethyl)benzonitrile (39.2 mg, 0.2 mmol, 1 equiv.), and diphenyl disulfide (65.4 mg, 0.3 mmol, 1.5 equiv.) according to **General Procedure 2.4**. The residue was purified by flash column, eluting with ethyl acetate/hexane (1:10) to afford the desired compound as a yellow solid (41.4 mg, 92% yield).

^{1}H NMR (400 MHz, $CDCl_3$) δ 7.58 – 7.50 (m, 2H), 7.36 – 7.31 (m, 2H), 7.29 – 7.19 (m, 5H), 4.10 (s, 2H).

^{13}C NMR (101 MHz, $CDCl_3$) δ 143.4, 134.7, 132.2, 130.9, 129.5, 129.1, 127.2, 118.8, 110.9, 39.2.

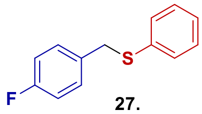

27.

Known compound reported by reference.[17]

(4-fluorobenzyl)(phenyl)sulfane (27): Synthesized from 4-fluorobenzyl bromide (24.9 µL, 0.2 mmol, 1 equiv.), and diphenyl disulfide (65.4 mg, 0.3 mmol, 1.5 equiv.) according to **General Procedure 2.4**. The residue was purified by flash column, eluting with ethyl acetate/hexane (1:20) to afford the desired compound as a white solid (39.2 mg, 90% yield).

^{1}H NMR (400 MHz, $CDCl_3$) δ 7.32 – 7.18 (m, 7H), 7.00 – 6.89 (m, 2H), 4.08 (s, 2H).

^{13}C NMR (101 MHz, $CDCl_3$) δ 161.9 (d, J = 245.8 Hz), 135.9, 133.3 (d, J = 3.3 Hz), 130.3 (d, J = 8.1 Hz), 130.2, 128.9, 126.6, 115.3 (d, J = 21.3 Hz), 38.5.

^{19}F NMR (376 MHz, $CDCl_3$) δ -115.4.

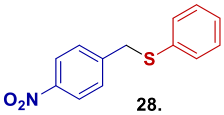

28.

Known compound reported by reference.[19]

(4-nitrobenzyl)(phenyl)sulfane (28): Synthesized from 4-nitrobenzyl bromide (43.2 mg, 0.2 mmol, 1 equiv.), and diphenyl disulfide (65.4 mg, 0.3 mmol, 1.5 equiv.) according to **General Procedure 2.4**. The residue was purified by flash column, eluting with ethyl acetate/hexane (1:10) to afford the desired compound as a yellow solid (41.6 mg, 85% yield).

^{1}H NMR (400 MHz, $CDCl_3$) δ 8.16 – 8.07 (m, 2H), 7.41 – 7.35 (m, 2H), 7.30 – 7.22 (m, 5H), 4.13 (s, 2H).

^{13}C NMR (101 MHz, $CDCl_3$) δ 145.6, 134.5, 131.1, 129.6, 129.1, 127.3, 123.7, 39.0.

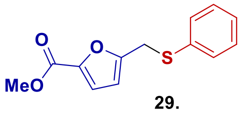

29.

methyl 5-((phenylthio)methyl)furan-2-carboxylate (29): Synthesized from methyl 5-(chloromethyl)furan-2-carboxylate (34.8 mg, 0.2 mmol, 1 equiv.), and diphenyl disulfide (65.4 mg, 0.3 mmol, 1.5 equiv.) according to **General Procedure 2.4**. The residue was purified by flash column, eluting with ethyl acetate/hexane (1:5) to afford the desired compound as a yellow oil (44.6 mg, 90% yield).

^{1}H NMR (400 MHz, $CDCl_3$) δ 7.37 – 7.31 (m, 2H), 7.31 – 7.20 (m, 3H), 7.06 (d, J = 3.4 Hz, 1H), 6.20 (d, J = 3.5 Hz, 1H), 4.12 (s, 2H), 3.88 (s, 3H).

^{13}C NMR (101 MHz, $CDCl_3$) δ 159.0, 156.0, 143.8, 134.7, 130.9, 129.0, 127.2, 119.1, 110.0, 51.9, 31.7.

HRMS (ESI-TOF) m/z: $[M + H]^+$ calcd. for $C_{13}H_{13}O_3S$ 249.0585, found 249.0581.

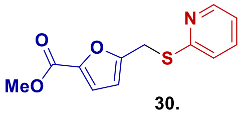

30.

methyl 5-((pyridin-2-ylthio)methyl)furan-2-carboxylate (30): Synthesized from methyl 5-(chloromethyl)furan-2-carboxylate (34.8 mg, 0.2 mmol, 1 equiv.), and 2,2′-dipyridyl disulfide (66.1 mg, 0.3 mmol, 1.5 equiv.) according to **General Procedure 2.4**. The residue was purified by flash column, eluting with ethyl acetate/hexane (1:5) to afford the desired compound as a yellow oil (42.3 mg, 85% yield).

^{1}H NMR (400 MHz, $CDCl_3$) δ 8.42 (ddd, J = 4.9, 1.9, 1.0 Hz, 1H), 7.46 (ddd, J = 8.1, 7.4, 1.9 Hz, 1H), 7.17 – 7.11 (m, 1H), 7.04 (d, J = 3.4 Hz, 1H), 7.01 – 6.95 (m, 1H), 6.37 – 6.32 (m, 1H), 4.48 (s, 2H), 3.85 (s, 3H).

^{13}C NMR (101 MHz, $CDCl_3$) δ 159.0, 156.9, 156.7, 149.4, 143.5, 136.1, 122.3, 119.9, 119.2, 109.9, 51.8, 26.2.

HRMS (ESI-TOF) m/z: $[M + H]^+$ calcd. for $C_{12}H_{12}NO_3S$ 250.0538, found 249.0535.

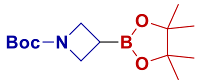

31.

Known compound reported by reference.[20]

***tert*-butyl 3-(4,4,5,5-tetramethyl-1,3,2-dioxaborolan-2-yl)azetidine-1-carboxylate (31):** Synthesized from tert-butyl 3-iodoazetidine-1-carboxylate (**1a**) (35.0 µL, 0.2 mmol, 1 equiv.) according to **General Procedure 2.5**. The residue was purified by flash column, eluting with ethyl acetate/hexane (1:10) to afford the desired compound as a yellow oil (39.6 mg, 70% yield).

^{1}H NMR (400 MHz, $CDCl_3$) δ 4.06 – 3.96 (m, 2H), 3.91 (t, J = 7.6 Hz, 2H), 2.18 – 2.05 (m, 1H), 1.43 (s, 9H), 1.26 (s, 12H).

^{13}C NMR (101 MHz, $CDCl_3$) δ 156.3, 83.7, 79.1, 50.5, 28.4, 24.8.

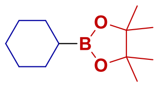

32.

Known compound reported by reference.[21]

2-cyclohexyl-4,4,5,5-tetramethyl-1,3,2-dioxaborolane (32): Synthesized from **Iodocyclohexane** (25.9 μL, 0.2 mmol, 1 equiv.) according to **General Procedure 2.5**. The residue was purified by flash column, eluting with hexane (100%) to afford the desired compound as a colorless oil (25.6 mg, 61% yield).

^{1}H NMR (400 MHz, $CDCl_3$) δ 1.72 – 1.53 (m, 5H), 1.45 – 1.16 (m, 17H), 1.05 – 0.93 (m, 1H).

^{13}C NMR (101 MHz, $CDCl_3$) δ 82.7, 53.4, 30.9, 29.7, 27.9, 27.1, 26.8, 24.7.

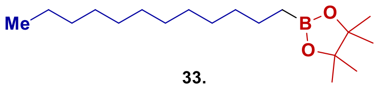

33.

Known compound reported by reference.[22]

2-dodecyl-4,4,5,5-tetramethyl-1,3,2-dioxaborolane (33): Synthesized from 1-iodododecane (49.3 μL, 0.2 mmol, 1 equiv.) according to **General Procedure 2.5**. The residue was purified by flash column, eluting with hexane (100%) to afford the desired compound as a colorless liquid (36.7 mg, 62% yield).

^{1}H NMR (400 MHz, $CDCl_3$) δ 1.44 – 1.35 (m, 2H), 1.33 – 1.18 (m, 30H), 0.93 – 0.82 (m, 3H), 0.76 (t, J = 7.8 Hz, 2H).

^{13}C NMR (101 MHz, $CDCl_3$) δ 82.8, 32.4, 31.9, 29.7, 29.7, 29.6, 29.4, 29.4, 24.8, 24.0, 22.7, 14.1, 11.1.

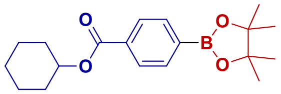

36.

Known compound reported by reference.[23]

cyclohexyl 4-(4,4,5,5-tetramethyl-1,3,2-dioxaborolan-2-yl)benzoate (34): Synthesized from cyclohexyl 4-iodobenzoate (66 mg, 0.2 mmol, 1 equiv.) according to **General Procedure 2.5**. The residue was purified by flash column, eluting with ethyl acetate/hexane (1:10) to afford the desired compound as a yellow oil (16.5 mg, 25% yield).

^{1}H NMR (400 MHz, $CDCl_3$) δ 8.02 (d, J = 8.3 Hz, 2H), 7.86 (d, J = 8.4 Hz, 2H), 5.09 – 4.97 (m, 1H), 2.02 – 1.90 (m, 2H), 1.86 – 1.73 (m, 2H), 1.67 – 1.54 (m, 3H), 1.52 – 1.31 (m, 15H).

^{13}C NMR (101 MHz, $CDCl_3$) δ 166.0, 134.6, 133.2, 128.5, 84.1, 73.2, 31.6, 25.5, 24.9, 23.7.

6. Radical Trapping Studies:

6.1. Dehalogenation:

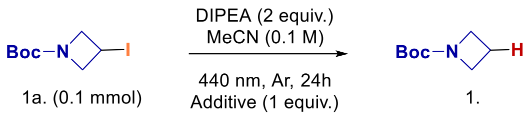

Additive	Yield of 1
TEMPO	Trace
1,1-DPE	Trace
BHT	Trace

tert-butyl 3-iodoazetidine-1-carboxylate (**1a**) (17.5 µL, 0.1 mmol, 1 equiv.), DIPEA (35 µL, 0.2 mmol, 2 equiv.), additive (1 equiv.), and acetonitrile (1 mL) were mixed in a 10 mL microwave vial equipped with a stir bar under argon atmosphere. The vial was sealed with a septum-cap and placed 2 cm away from two 440 nm blue LED (40W). The temperature was kept at approximately 35 °C through cooling with a fan (heating caused by LED lamp). After being stirred for 24 hours, the reaction mixture was directly transferred into a scintillation vial, and concentrated under vacuum. Aliquots were collected for HRMS analysis and the crude yields were obtained using ^{1}H NMR analysis using dibromomethane as internal standard.

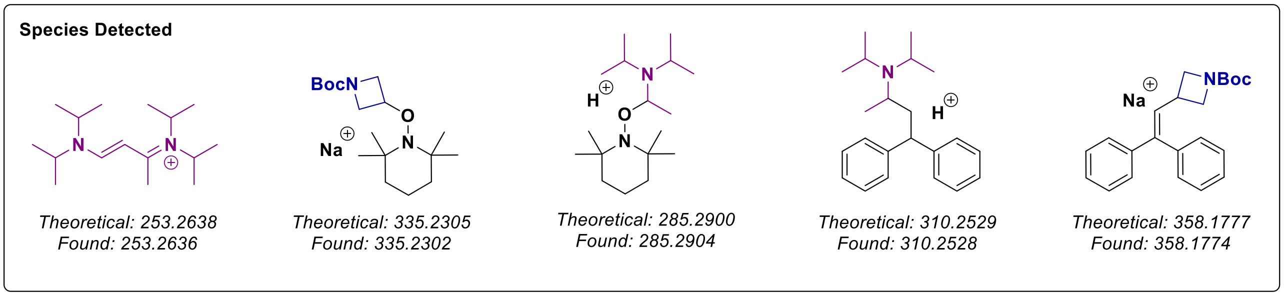

6.2. Chalcogenation:

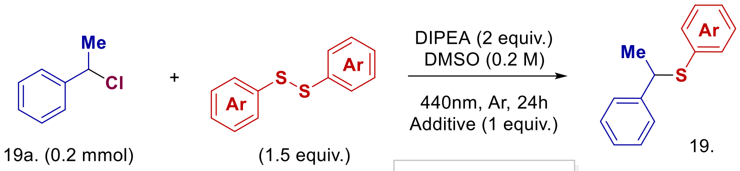

Additive	Yield of 19
TEMPO	35%
1,1-DPE	< 10%
BHT	< 10%

1-chloroethyl-benzene (26.4 µL, 0.2 mmol, 1 equiv.), diphenyl disulfide (65.4 mg, 0.3 mmol, 1.5 equiv.), DIPEA (70 µL, 0.4 mmol, 2 equiv.), additive (1 equiv.), and DMSO (1 mL) were mixed in a 10 mL microwave vial equipped with a stir bar under argon atmosphere. The vial was sealed with a septum-cap and placed 2 cm away from two 440 nm blue LED (40W). The temperature was kept at approximately 35 °C through cooling with a fan (heating caused by LED lamp). After being stirred for 24 hours, the reaction mixture was the reaction mixture was poured into 20 mL of water, and the resulting mixture was extracted with DCM (3 × 20 mL). The combined organic phase was dried over $NaSO_4$, filtered, and concentrated under vacuum. Aliquots were collected for HRMS analysis and the crude yields were obtained using ^{1}H NMR analysis using dibromomethane as internal standard.

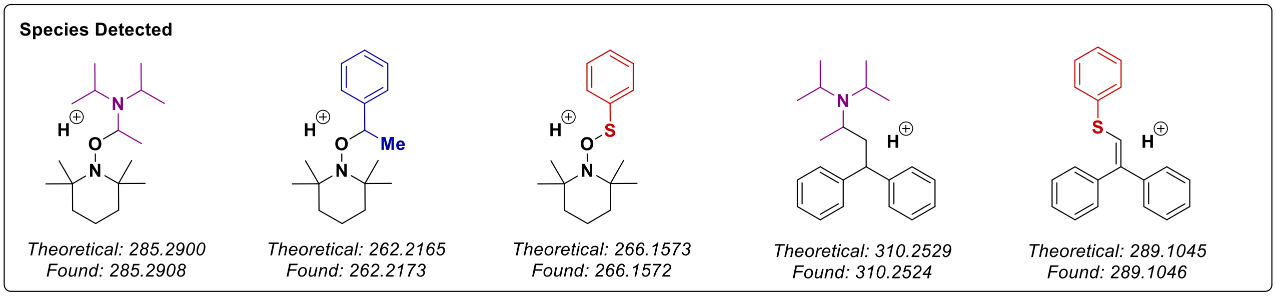

7. Dehalogenation Reaction Using Acetonitrile-d_3:

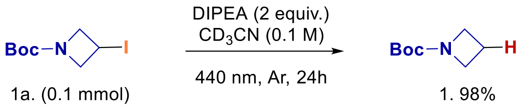

tert-butyl 3-iodoazetidine-1-carboxylate (**1a**) (17.5 µL, 0.1 mmol, 1 equiv.), DIPEA (35 µL, 0.2 mmol, 2 equiv.), and acetonitrile-d_3 (1 mL) were mixed in a 10 mL microwave vial equipped with a stir bar under argon atmosphere. The vial was sealed with a septum-cap and placed 2 cm away from two 440 nm blue LED (40W). The temperature was kept at approximately 35 °C through cooling with a fan (heating caused by LED lamp). After being stirred for 24 hours, the reaction mixture was directly transferred into a scintillation vial, and concentrated under vacuum. Aliquots were collected for GC-MS analysis and the crude yields were obtained using ^{1}H NMR analysis using dibromomethane as internal standard.

*The resulting defunctionalized product **1** exhibited no incorporation of deuterium, indicating that the hydrogen-atom transfer (HAT) event does not originate from the solvent. Instead, this suggests that DIPEA is the operative hydrogen donor under the reaction conditions.*

8. UV-Vis Studies:

8.1. Dehalogenation: Investigating the possibility of donor-acceptor complex

Preparation of Stock Solutions:

1. *tert*-butyl-3-iodoazetidine-1-carboxylate (**1a**): 0.1 mmol/mL in MeCN.
2. DIPEA: 0.2 mmol/mL in MeCN.

UV-Vis Spectra Experiments:

- Final concentrations: 0.05 mmol/mL of **1a**, and 0.1 mmol/mL of DIPEA.

Experiments:

- Experiment A: 1 mL of stock solution 1, diluted to 2 mL in MeCN.
- Experiment B: 1 mL of stock solution 2, diluted to 2 mL in MeCN.
- Experiment C: 1 mL of stock solution 1 and 1 mL of stock solution 2.

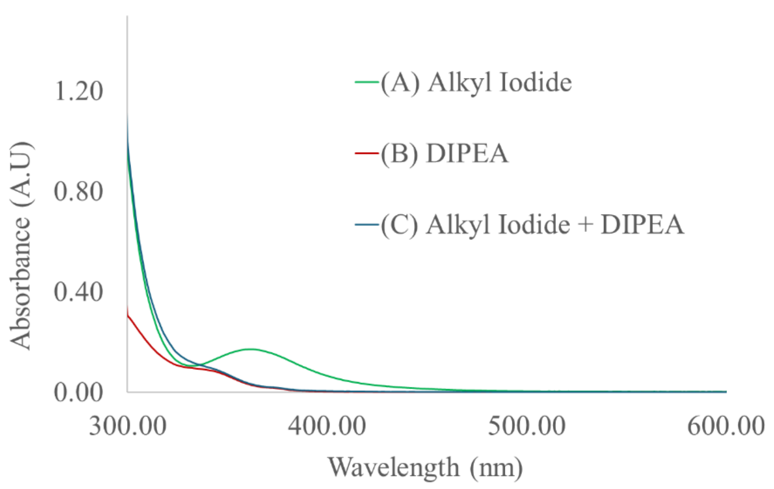

The absence of a charge-transfer band upon mixing the two components suggests a low likelihood of photoactive aggregate formation.

8.2. Dehalogenation: Time-dependent absorption spectra of reaction crude

UV-Vis Spectra Experiments:

- Final concentrations: 0.05 mmol/mL of **1a**, and 0.1 mmol/mL of DIPEA.

Experiments:

- Experiment A: *tert*-butyl 3-iodoazetidine-1-carboxylate (**1a**) (17.5 µL, 0.1 mmol, 1 equiv.), DIPEA (35 µL, 0.2 mmol, 2 equiv.), and acetonitrile (1 mL) were added into a uv-vis cuvette, diluted to 2 mL MeCN and absorption spectra was recorded.
- Experiment B: *tert*-butyl 3-iodoazetidine-1-carboxylate (**1a**) (17.5 µL, 0.1 mmol, 1 equiv.), DIPEA (35 µL, 0.2 mmol, 2 equiv.), and acetonitrile (1 mL) were mixed in a 10 mL microwave vial equipped with a stir bar under argon atmosphere. After being stirred for 1800 seconds, the reaction mixture was directly transferred into a uv-vis cuvette, diluted to 2 mL MeCN and absorption spectra was recorded.

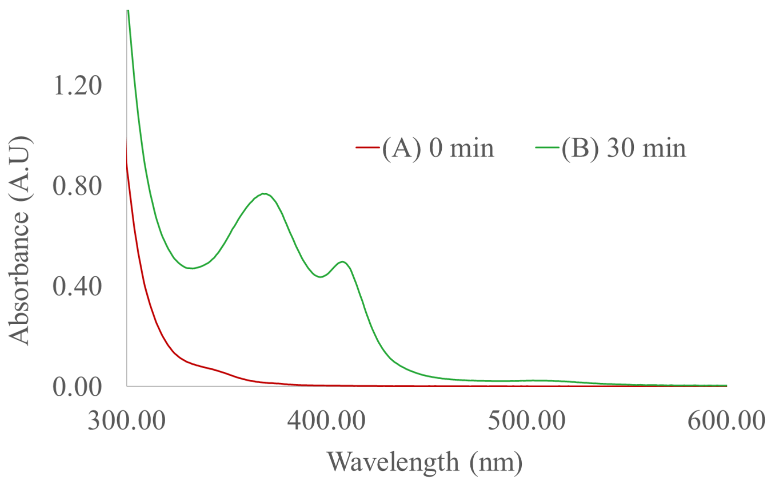

UV–Vis spectra at 0 and 30 minutes showed increased absorbance with distinct peaks at 370 and 410 nm, consistent with in situ formation of a photoactive species.

8.3. Chalcogenation: Investigating the possibility of donor-acceptor complex

Preparation of Stock Solutions:

1. 3-fluorobenzyl bromide (**22a**): 0.2 mmol/mL in MeCN.
2. diphenyl disulfide (Ph_2S_2): 0.3 mmol/mL in MeCN
3. DIPEA: 0.4 mmol/mL in MeCN.

UV-Vis Spectra Experiments:

- Final concentrations: 0.1 mmol/mL of **22a**, 0.15 mmol/mL of Ph_2S_2 and 0.2 mmol/mL of DIPEA.

Experiments:

- Experiment A: 1 mL of stock solution 1, diluted to 2 mL in MeCN.
- Experiment B: 1 mL of stock solution 2, diluted to 2 mL in MeCN.
- Experiment C: 1 mL of stock solution 3, diluted to 2 mL in MeCN.
- Experiment C: 1 mL of stock solution 1, and 1 mL of stock solution 2.

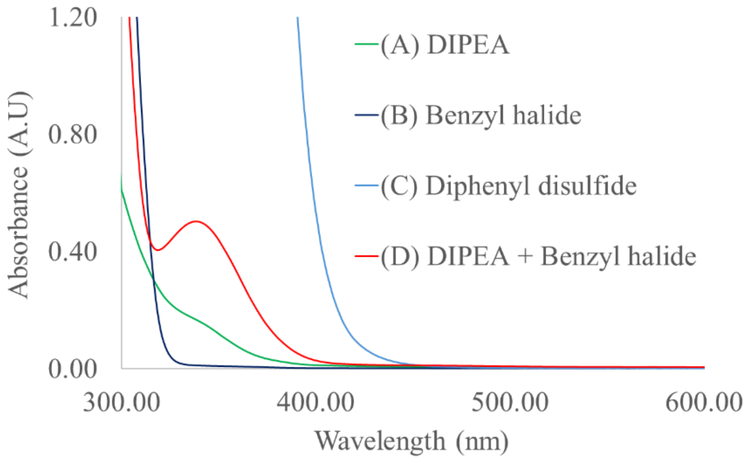

*The observation of a charge-transfer band upon mixing **22a** with DIPEA indicates the potential formation of a photoactive aggregate.*

9. Reaction Profile:

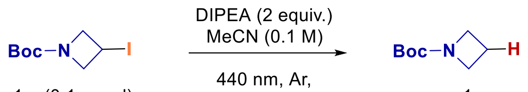

tert-butyl 3-iodoazetidine-1-carboxylate (**1a**) (17.5 µL, 0.1 mmol, 1 equiv.), DIPEA (35 µL, 0.2 mmol, 2 equiv.), and acetonitrile (1 mL) were mixed in a 10 mL microwave vial equipped with a stir bar under argon atmosphere. The vial was sealed with a septum-cap and placed 2 cm away from two 440 nm blue LED (40W). The temperature was kept at approximately 35 °C through cooling with a fan (heating caused by LED lamp). After being stirred for a fixed time period (0 s, 900 s, 1800 s, 2700 s, 3600 s, and 4500 seconds), the reaction mixture was directly transferred into a scintillation vial, and concentrated under vacuum. The crude yields were obtained using ^{1}H NMR analysis using dibromomethane as internal standard. The same experiment was reproduced three times to obtain the average value.

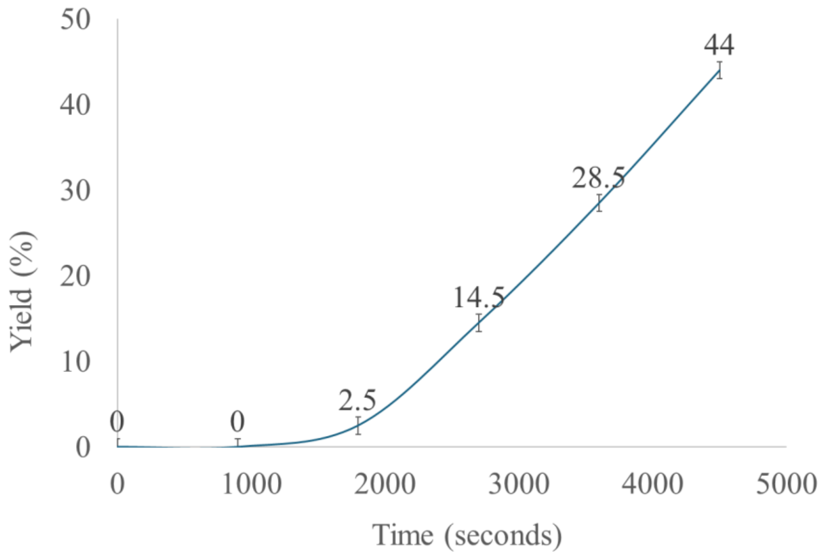

10. Photon-Flux Calculation.[24]

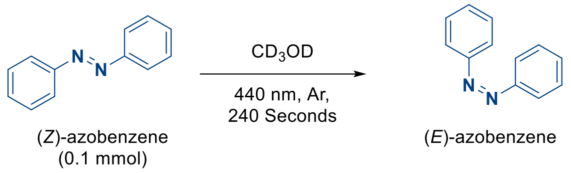

(Z)-azobenzene (18.2 mg, 0.1 mmol, 1 equiv.), and methanol-d^5 (1 mL) were mixed in a 10 mL microwave vial equipped with a stir bar under argon atmosphere. The vial was sealed with a septum-cap and placed 2 cm away from two 440 nm blue LED (40W). The temperature was kept at approximately 35 °C through cooling with a fan (heating caused by LED lamp). The mixture was stirred under irradiation for 240 seconds, after which it was directly transferred to an NMR tube. The extent of isomerization was quantified by comparing the integrals of the (Z)- and (E)-isomers in the resulting ^{1}HNMR spectrum. The same experiment was reproduced three times to obtain the average value.

$$\Phi = \frac{M}{F \times t \times f} \qquad (1)$$

$$f = 1 - 10^{-A} \qquad (2)$$

Φ = Quantum Yield, M = Moles of product formed, F = Photon flux, t = Time in seconds, f = Fraction of absorbance

Φ of azobenzene w.r.t the reaction condition was obtained from literature reports. f = 0.99, as absorbance of reaction mixture is >3 (*vide infra*).

Substituting these data into equation 1,

$$0.3047 = \frac{1.98 \times 10^{-5}\ \text{mol}}{F \times 240\ \text{s} \times 0.99} \qquad (3)$$

Thus, photon flux (F),

$$\text{F} = \frac{1.98 \times 10^{-5}\ \text{mol}}{0.3047 \times 240\ \text{s} \times 0.99} \qquad (4)$$

$$= 2.73 \times 10^{-8}\ \text{mol s}^{-1}$$

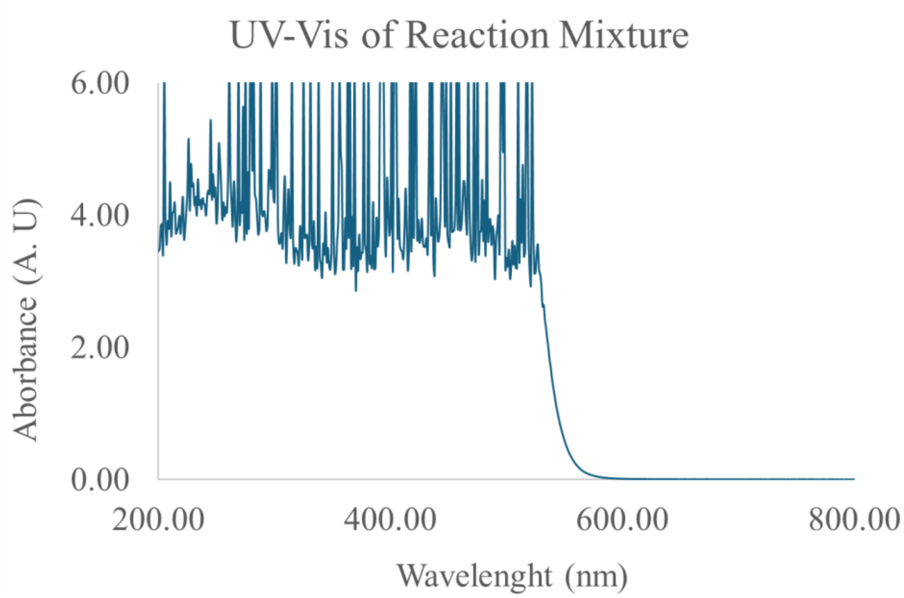
UV-Vis of Reaction Mixture
6.00
4.00
2.00
0.00
Aborbance (A. U)
200.00
400.00
600.00
800.00
Wavelenght (nm)

11. Quantum Yield Calculation.[24]

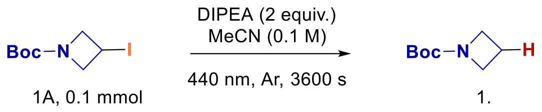

tert-butyl 3-iodoazetidine-1-carboxylate (**1A**) (17.5 µL, 0.2 mmol, 1 equiv.), DIPEA (35 µL, 0.2 mmol, 2 equiv.), and acetonitrile (1 mL) were mixed in a 10 mL microwave vial equipped with a stir bar under argon atmosphere. The vial was sealed with a septum-cap and placed 2 cm away from two 440 nm blue LED (40W). The temperature was kept at approximately 35 °C through cooling with a fan (heating caused by LED lamp). After being stirred for 2700 seconds, the reaction mixture was directly transferred into a scintillation vial, and concentrated under vacuum. Crude yields were determined by ^{1}HNMR analysis, employing dibromomethane as an internal standard. The same experiment was reproduced three times to obtain the average value.

Substituting these data into equation 1,

$$\Phi = \frac{1.45 \times 10^{-5}\ \text{mol}}{2.73 \times 10^{-8}\ \text{mol s}^{-1} \times 2700\ \text{s} \times 0.01} \quad (5)$$

Photon flux (F) = 2.73×10^{-8} mol s^{-1}, obtained from equation (4)

$f = 0.01$, as there is minimal absorbance in UV-Vis of reaction mixture.

Thus, Quantum yield (Φ),

$$\Phi \sim 20$$

12. ^{1}H NMR, ^{19}F NMR, ^{13}C NMR Spectra

^{1}H NMR (400 MHz, $CDCl_3$)

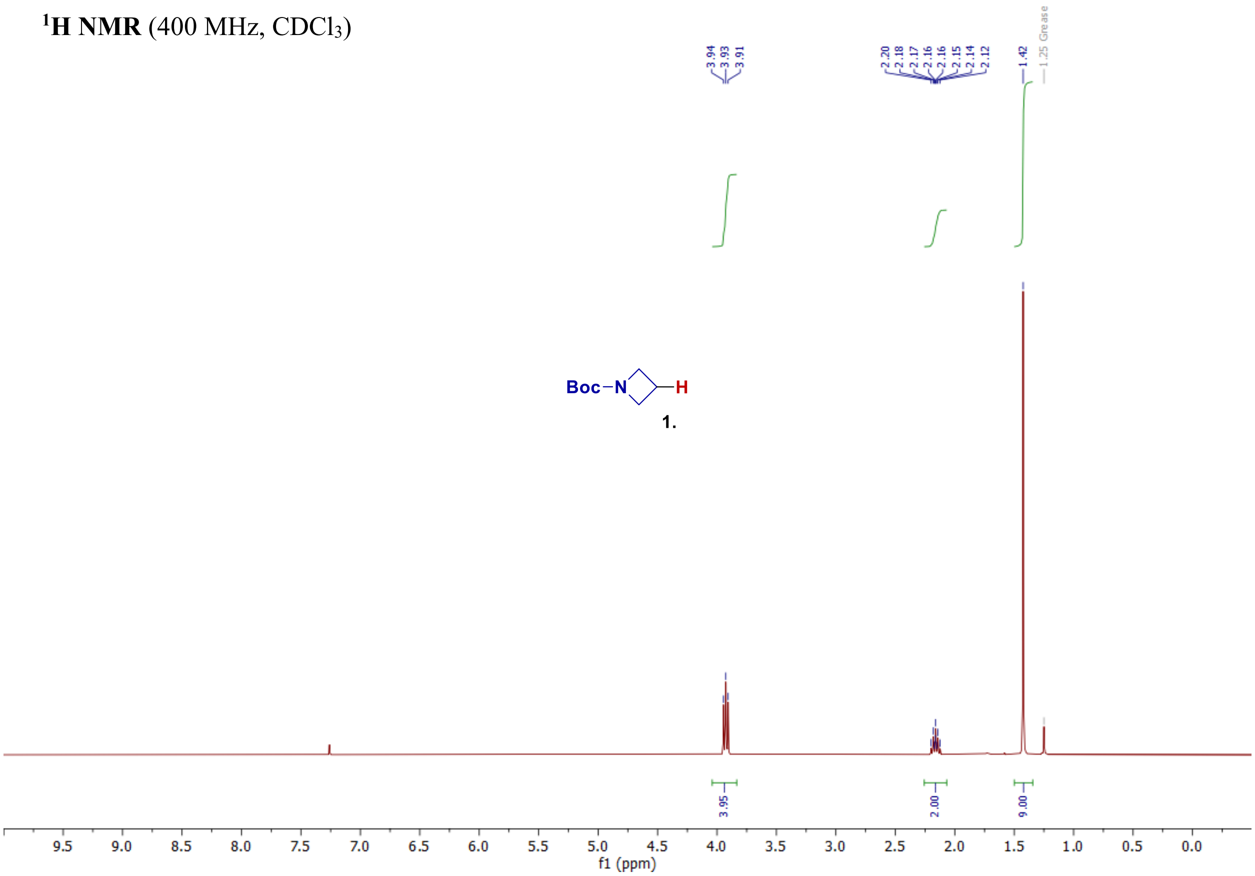

^{13}C NMR (101 MHz, $CDCl_3$)

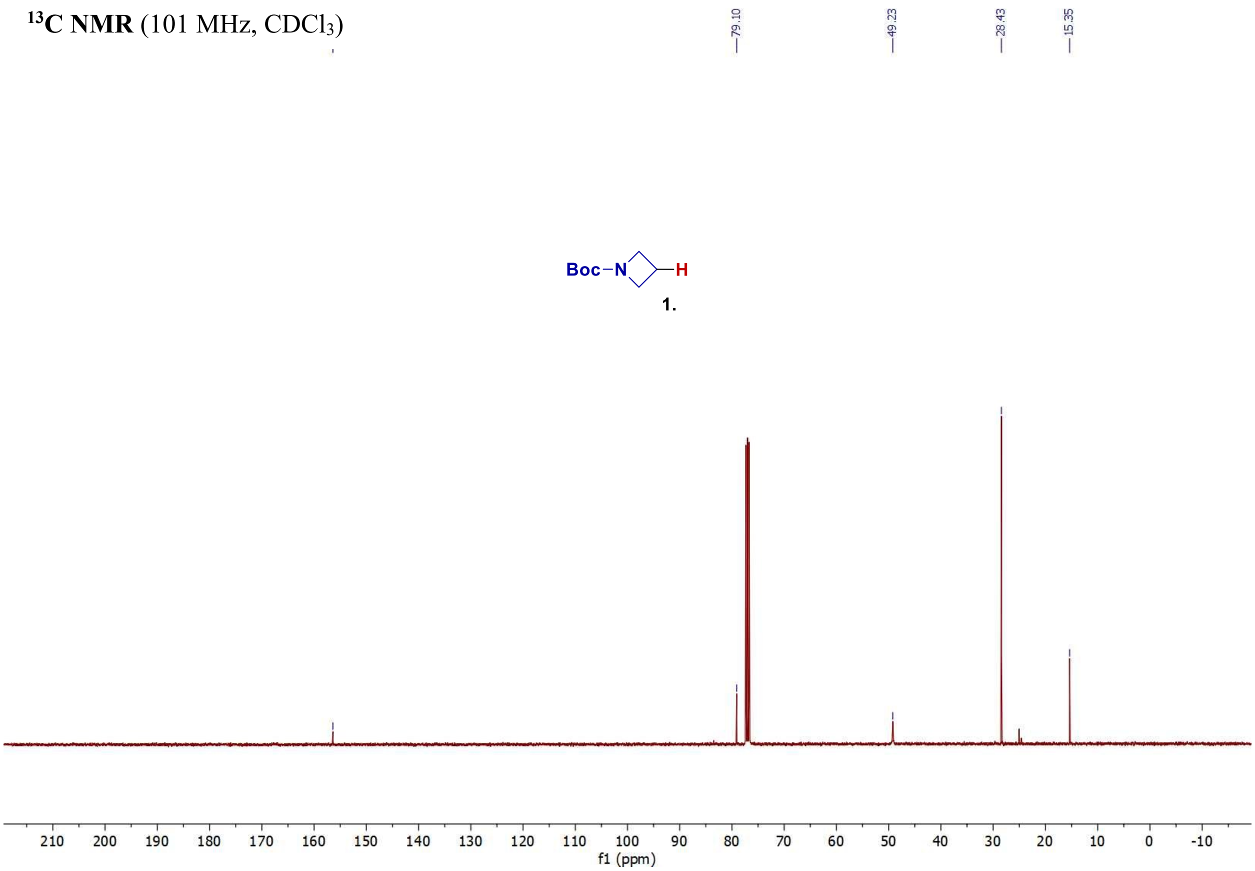

^{1}H NMR (400 MHz, $CDCl_3$)

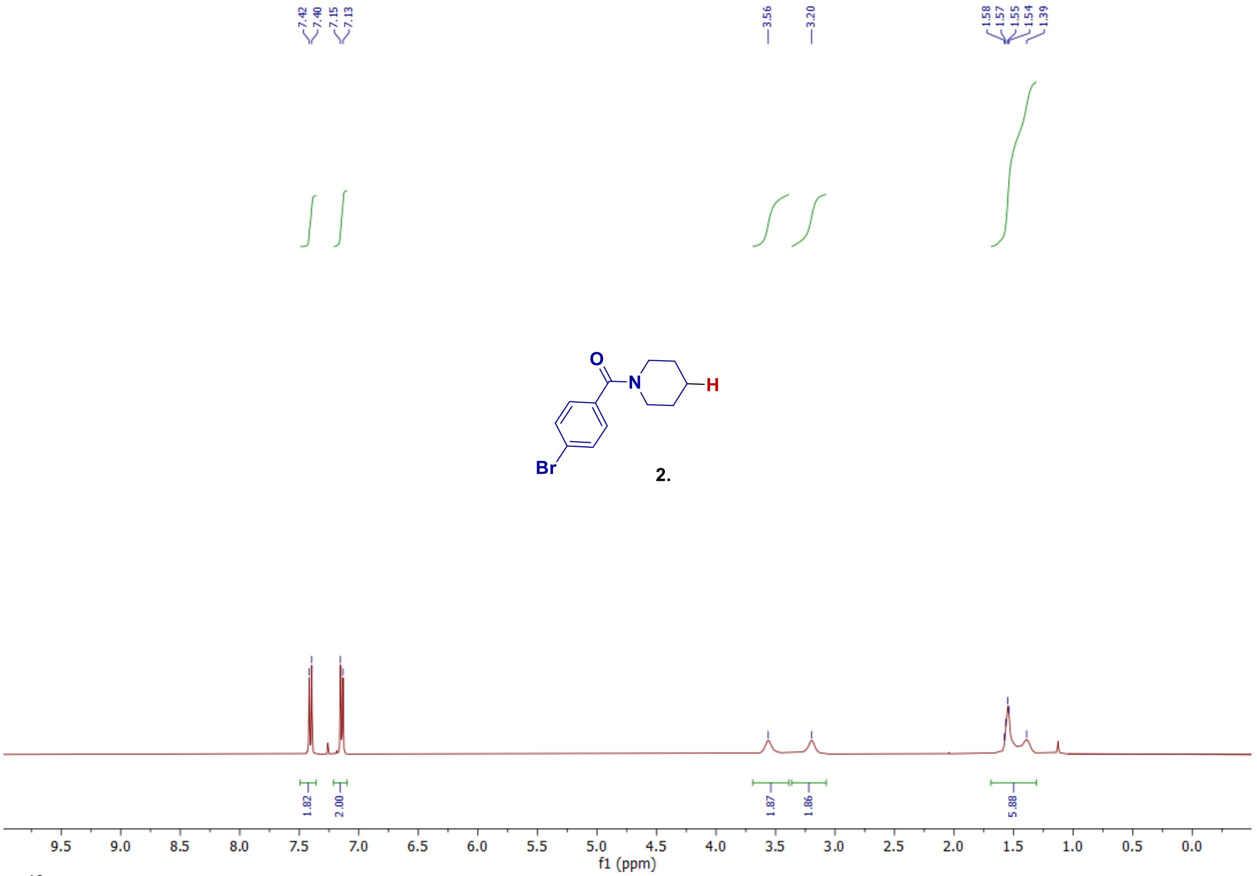

^{13}C NMR (101 MHz, $CDCl_3$)

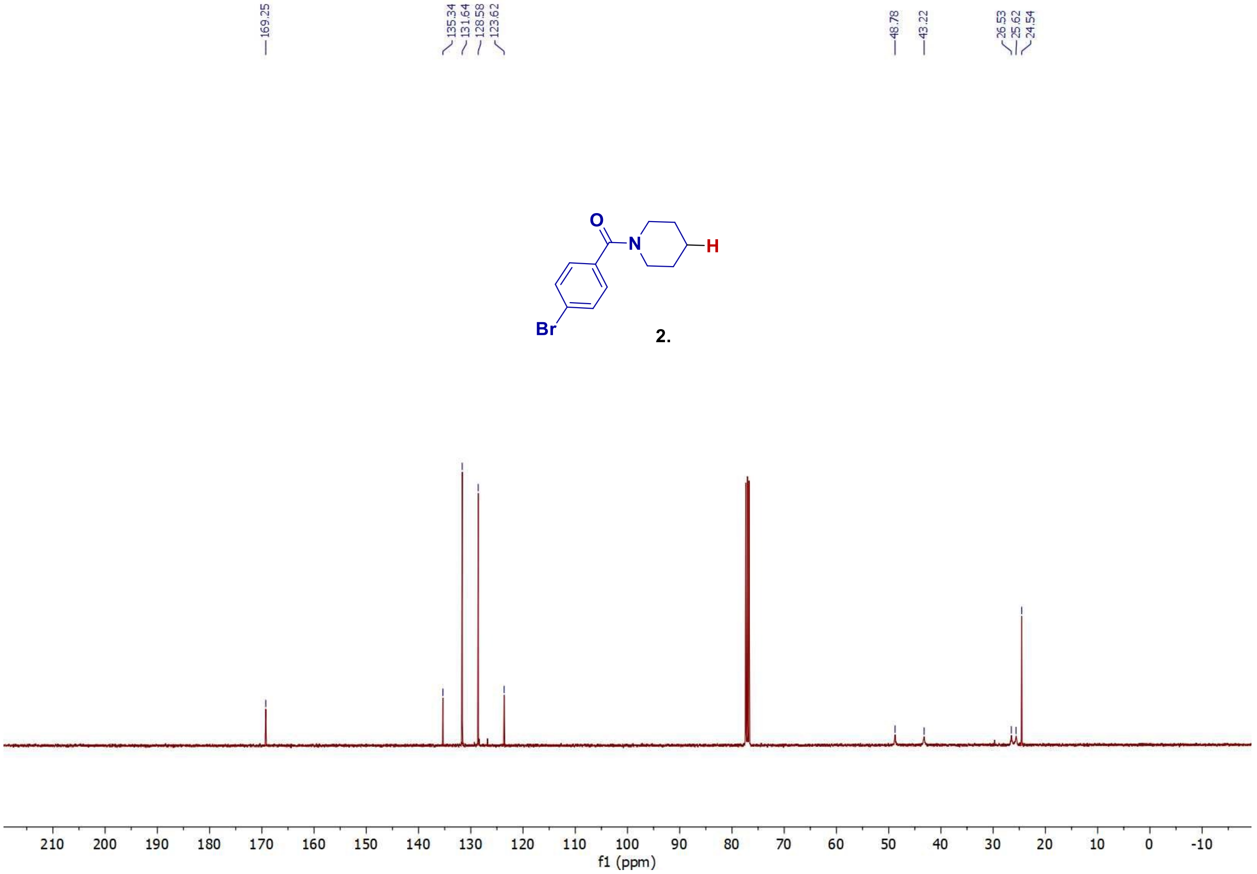

^{1}H NMR (400 MHz, $CDCl_3$)

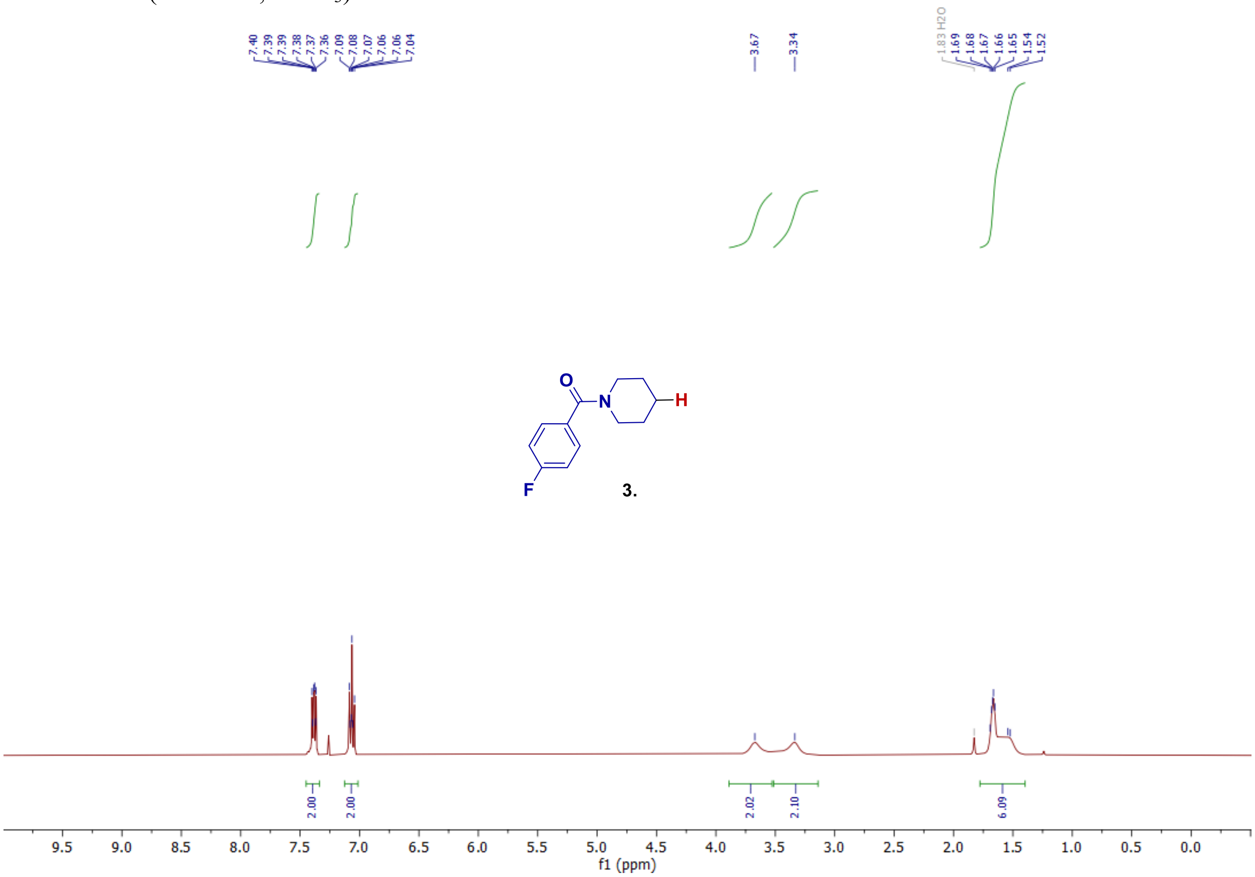

^{13}C NMR (101 MHz, $CDCl_3$)

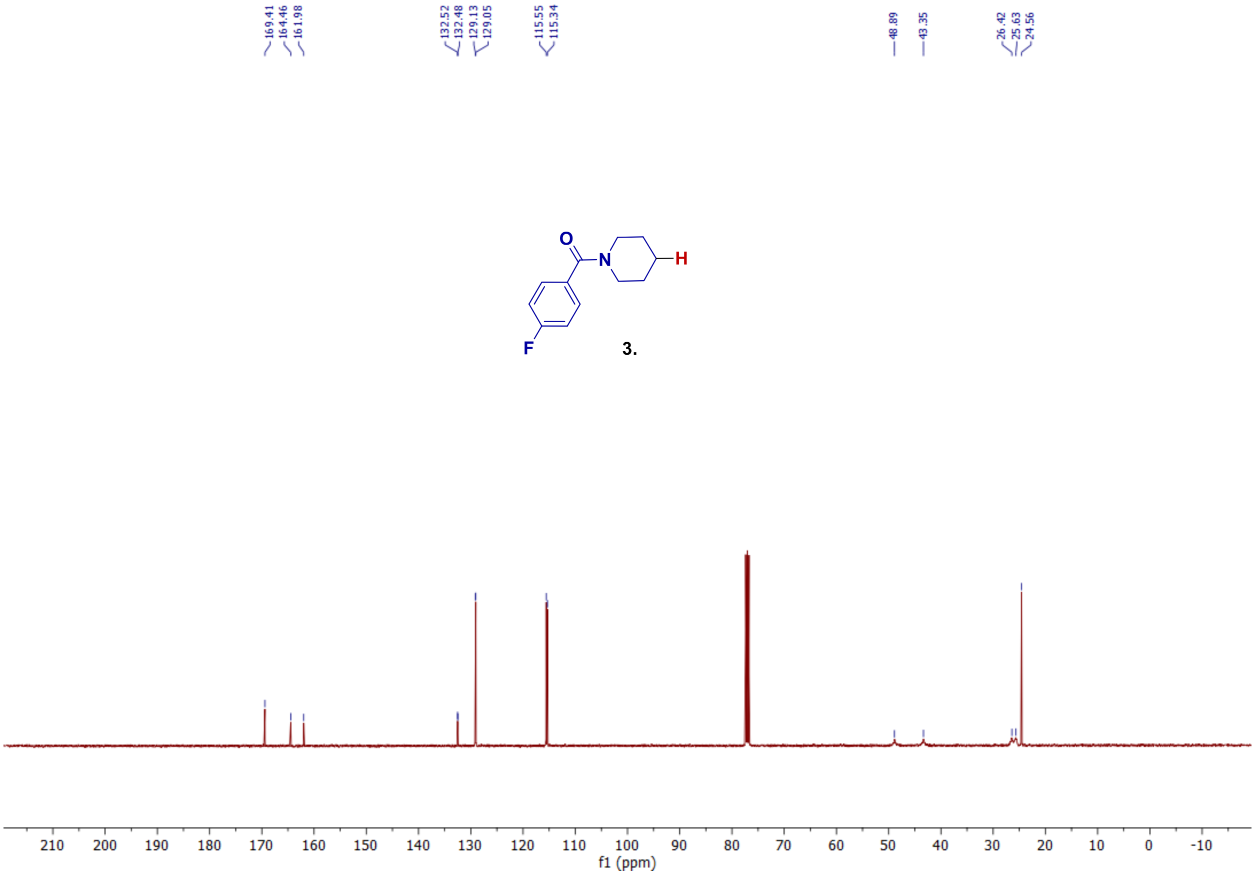

^{1}H NMR (400 MHz, $CDCl_3$)

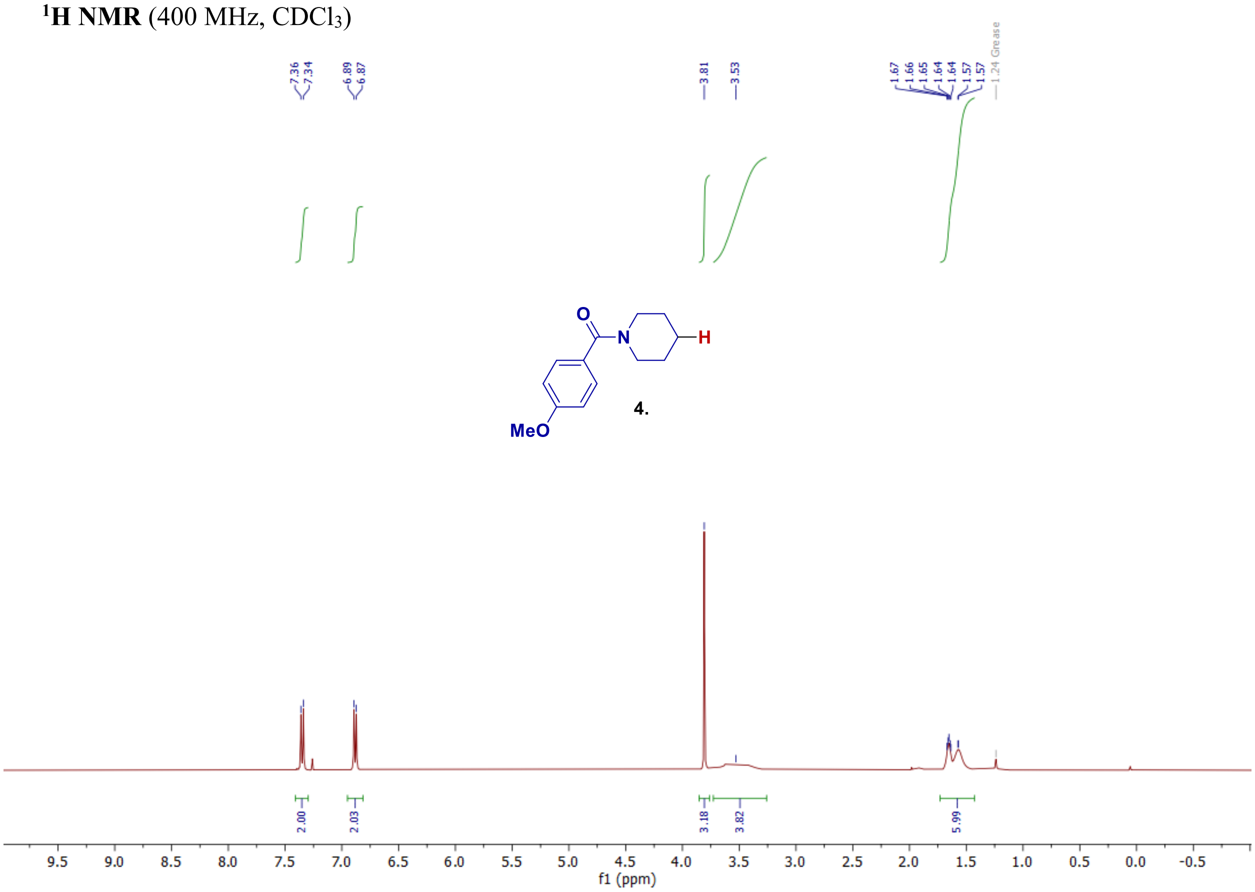

^{13}C NMR (101 MHz, $CDCl_3$)

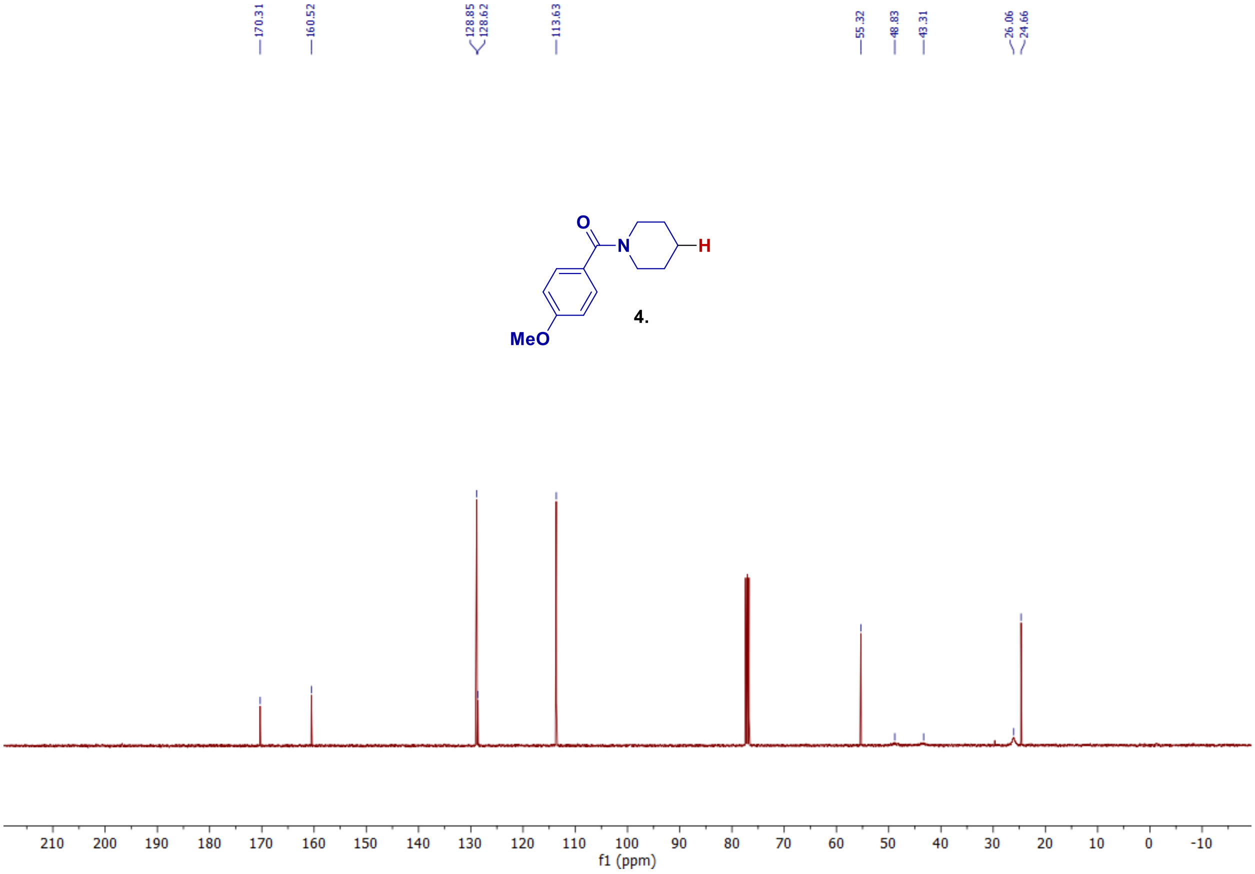

^{1}**H NMR** (400 MHz, $CDCl_3$)

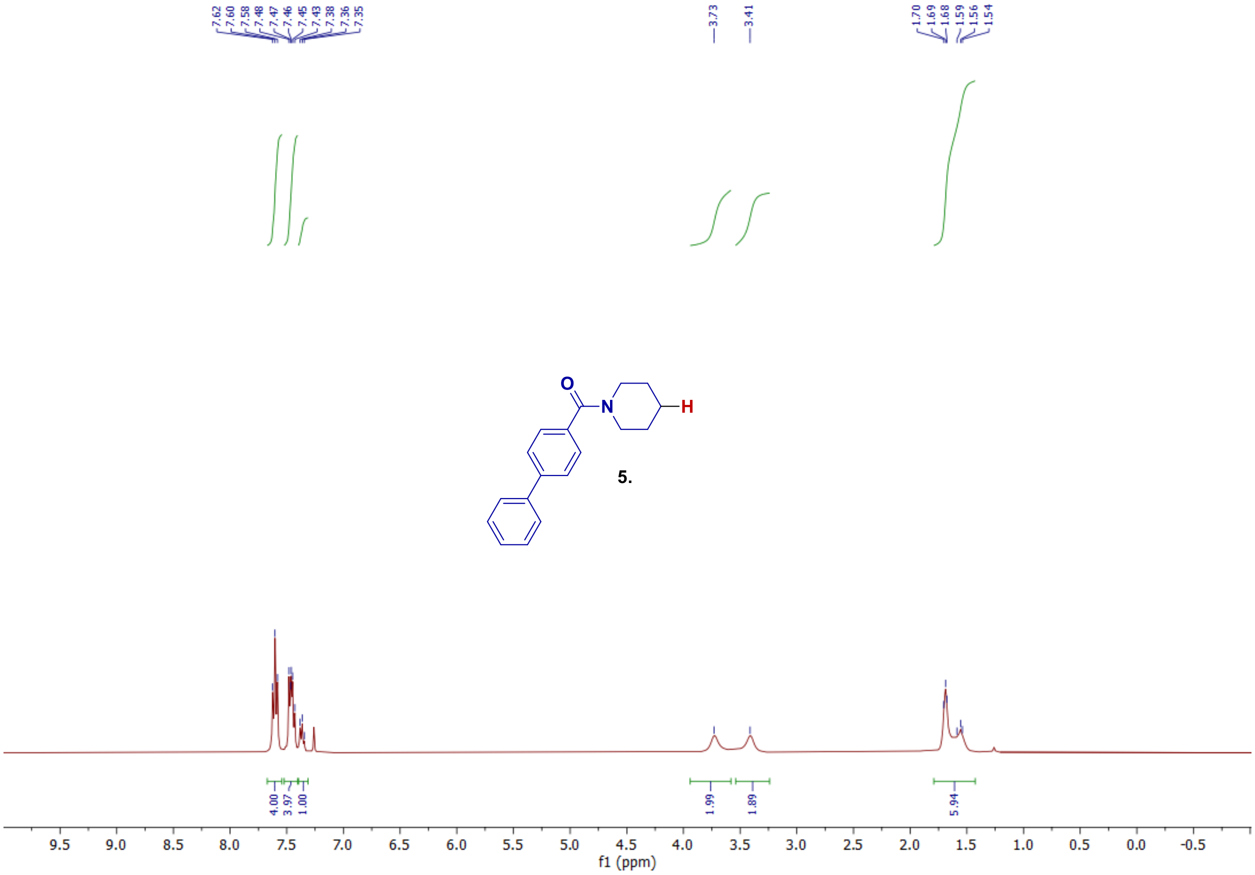

^{13}**C NMR** (101 MHz, $CDCl_3$)

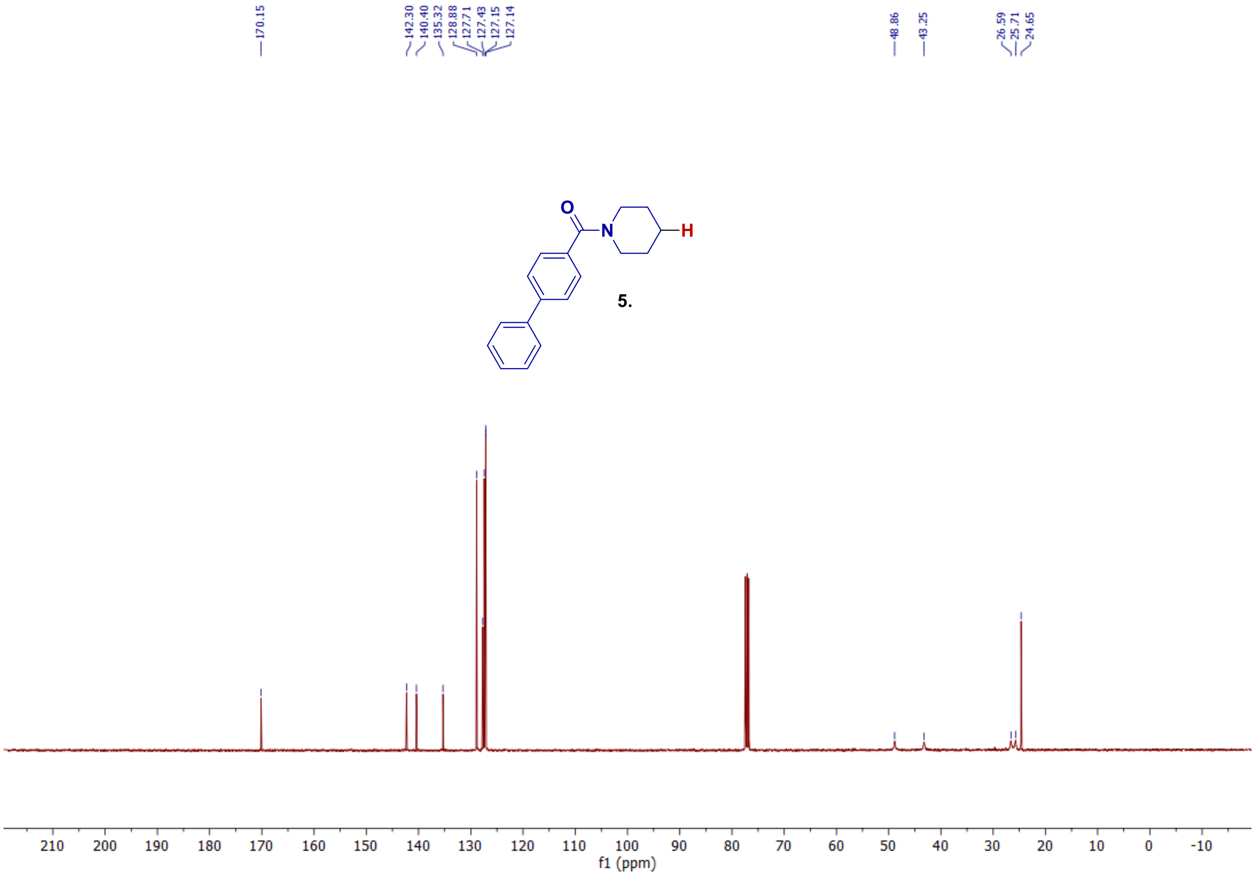

^{1}H NMR (400 MHz, $CDCl_3$)

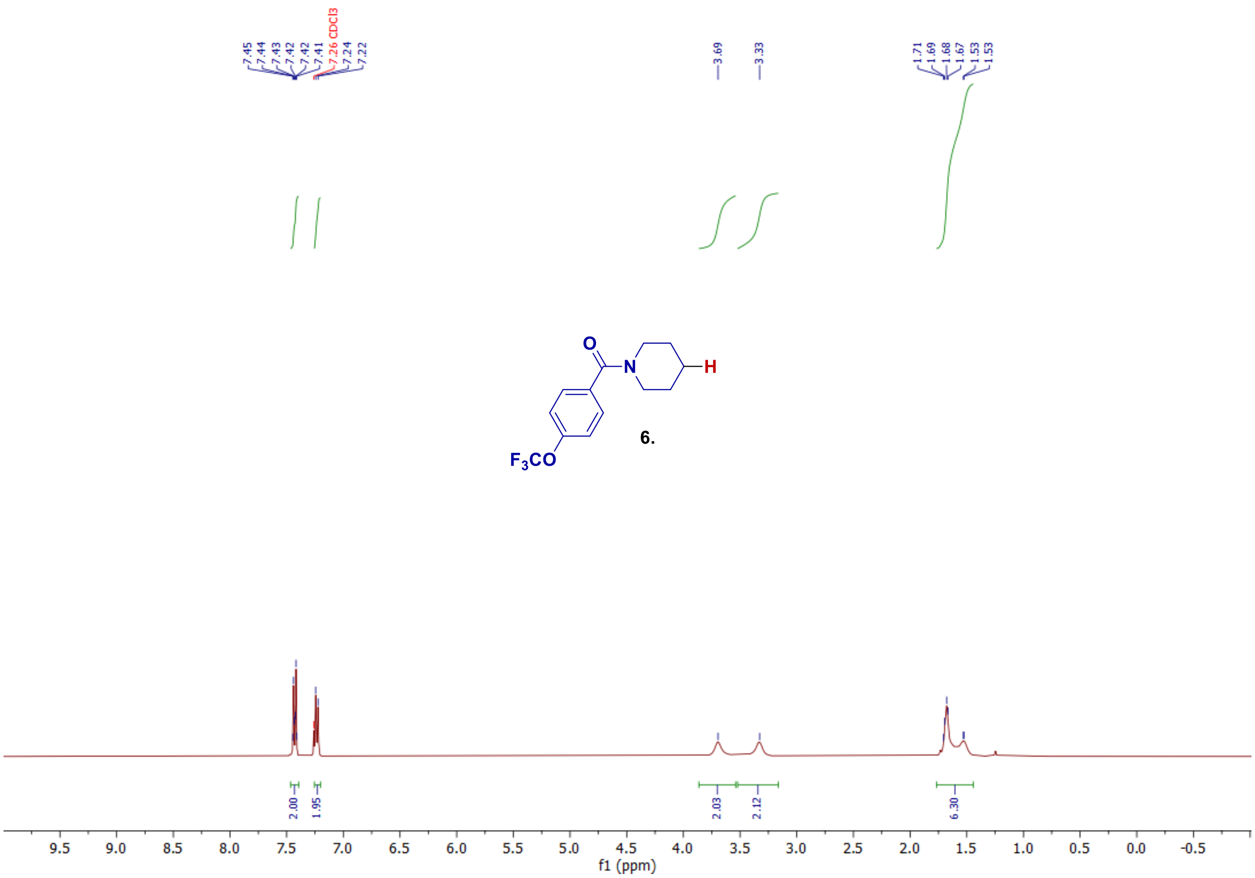

^{13}C NMR (101 MHz, $CDCl_3$)

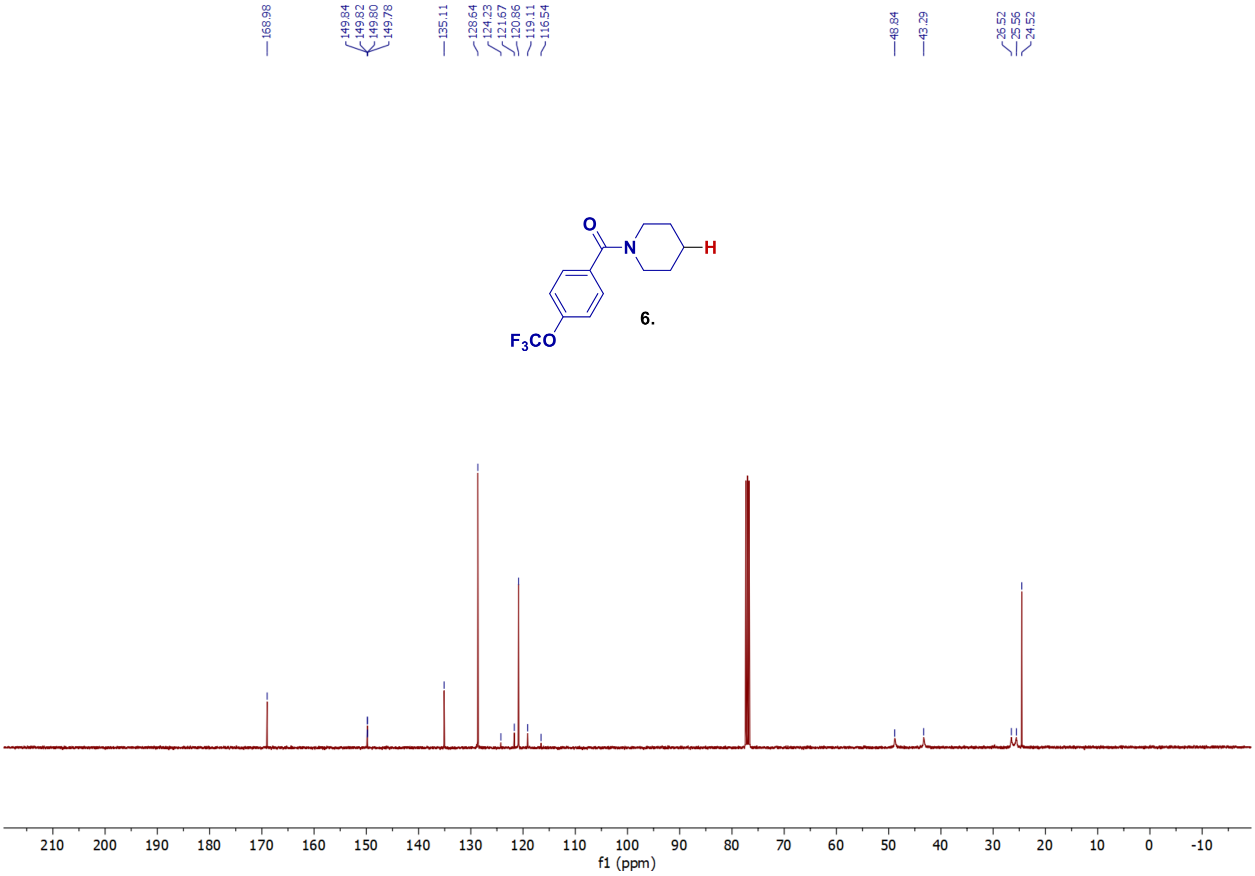

^{19}F NMR (377 MHz, $CDCl_3$)

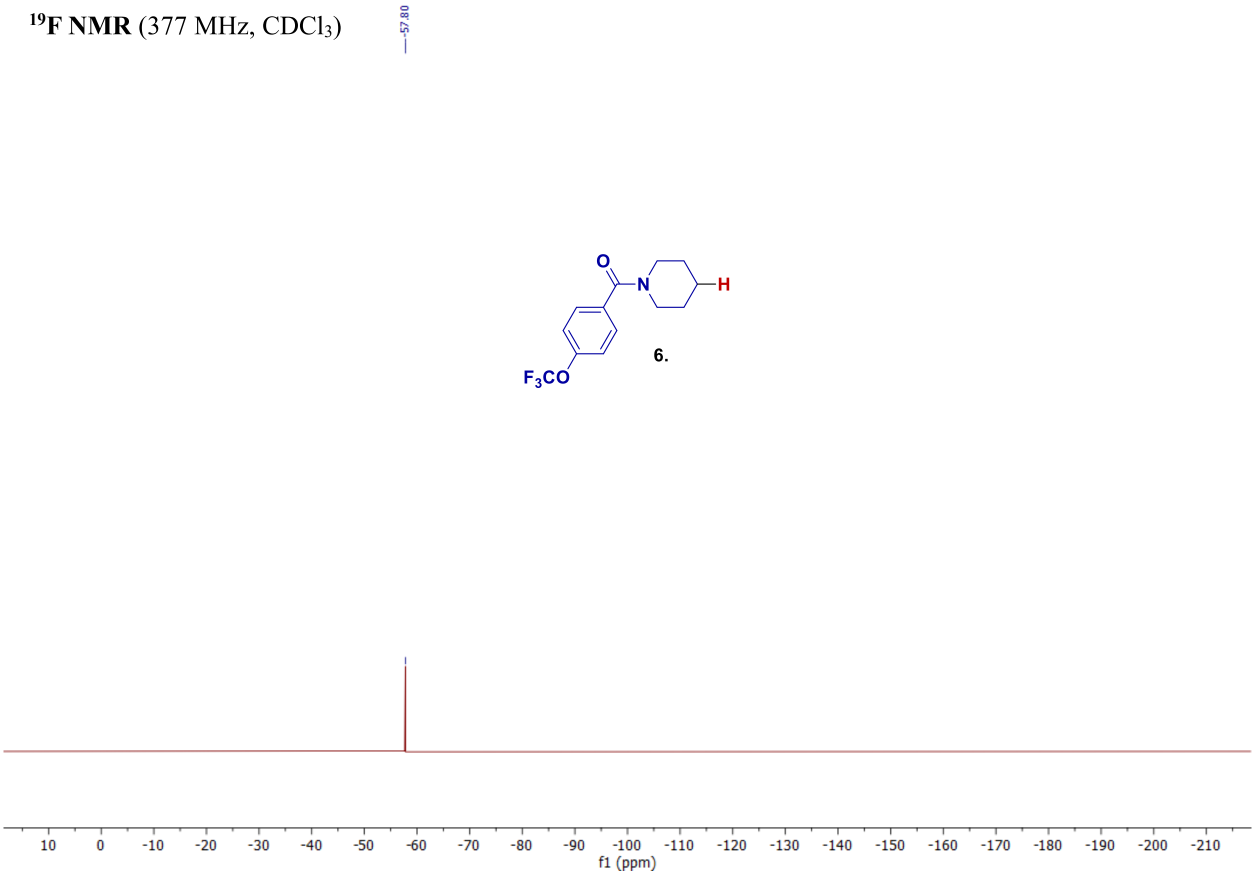

6.

^{1}H NMR (400 MHz, $CDCl_3$)

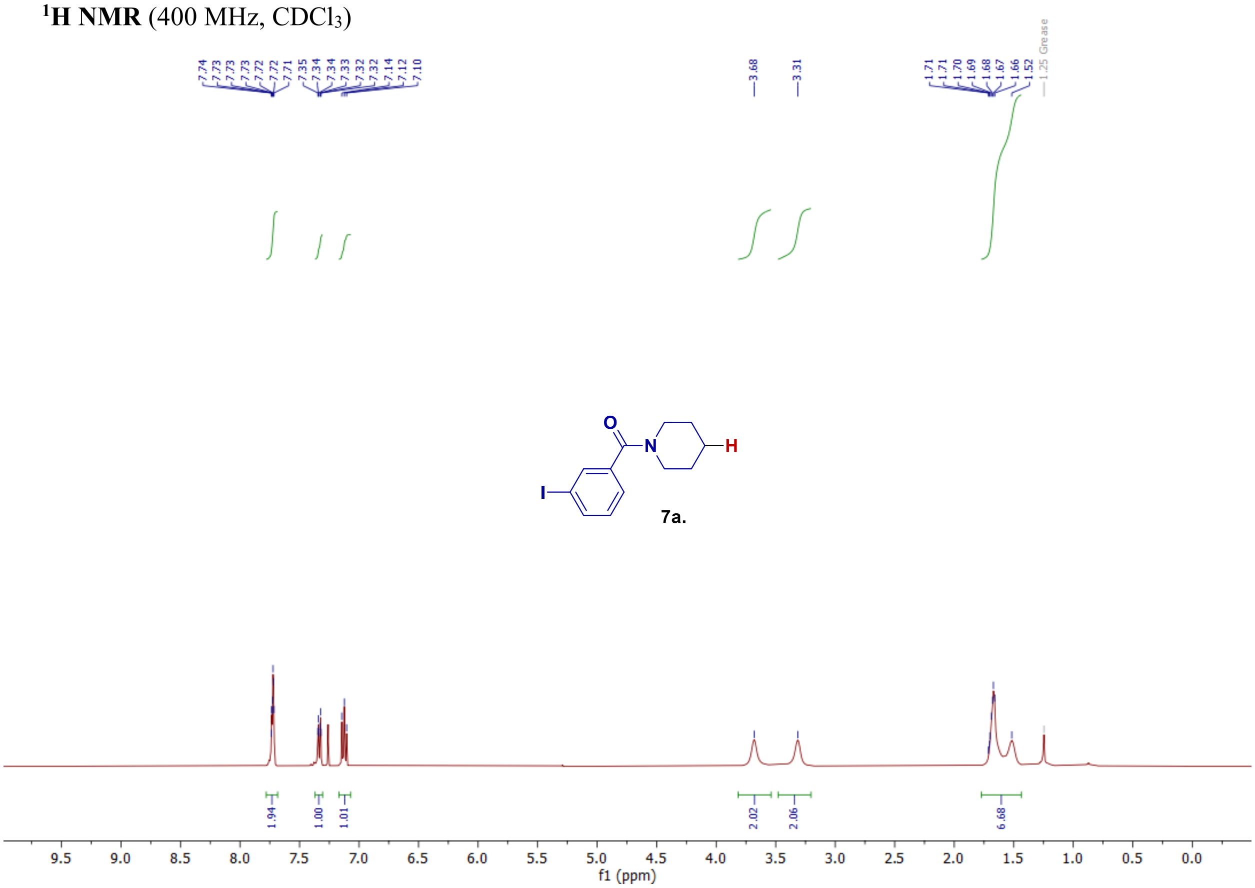

^{13}C NMR (101 MHz, $CDCl_3$)

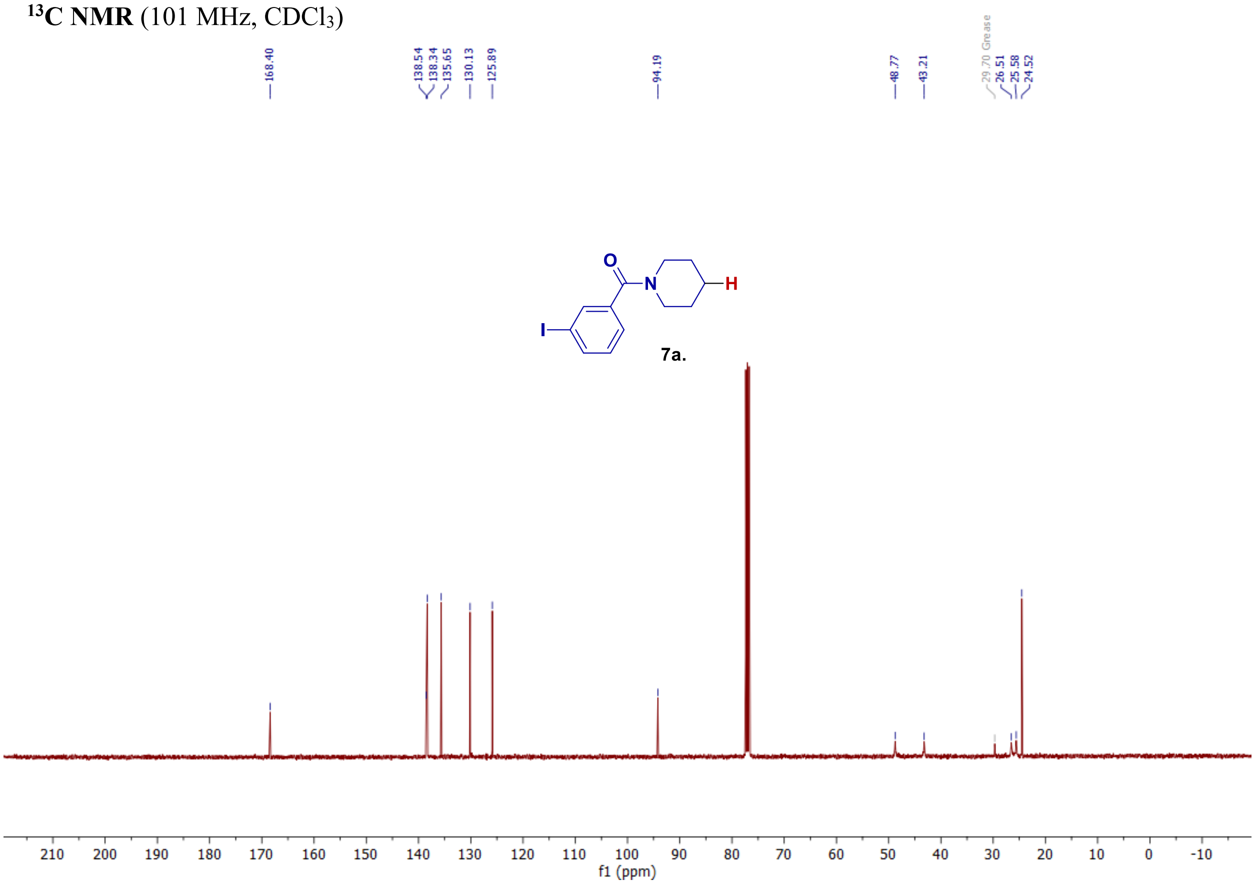

^{1}H NMR (400 MHz, $CDCl_3$)

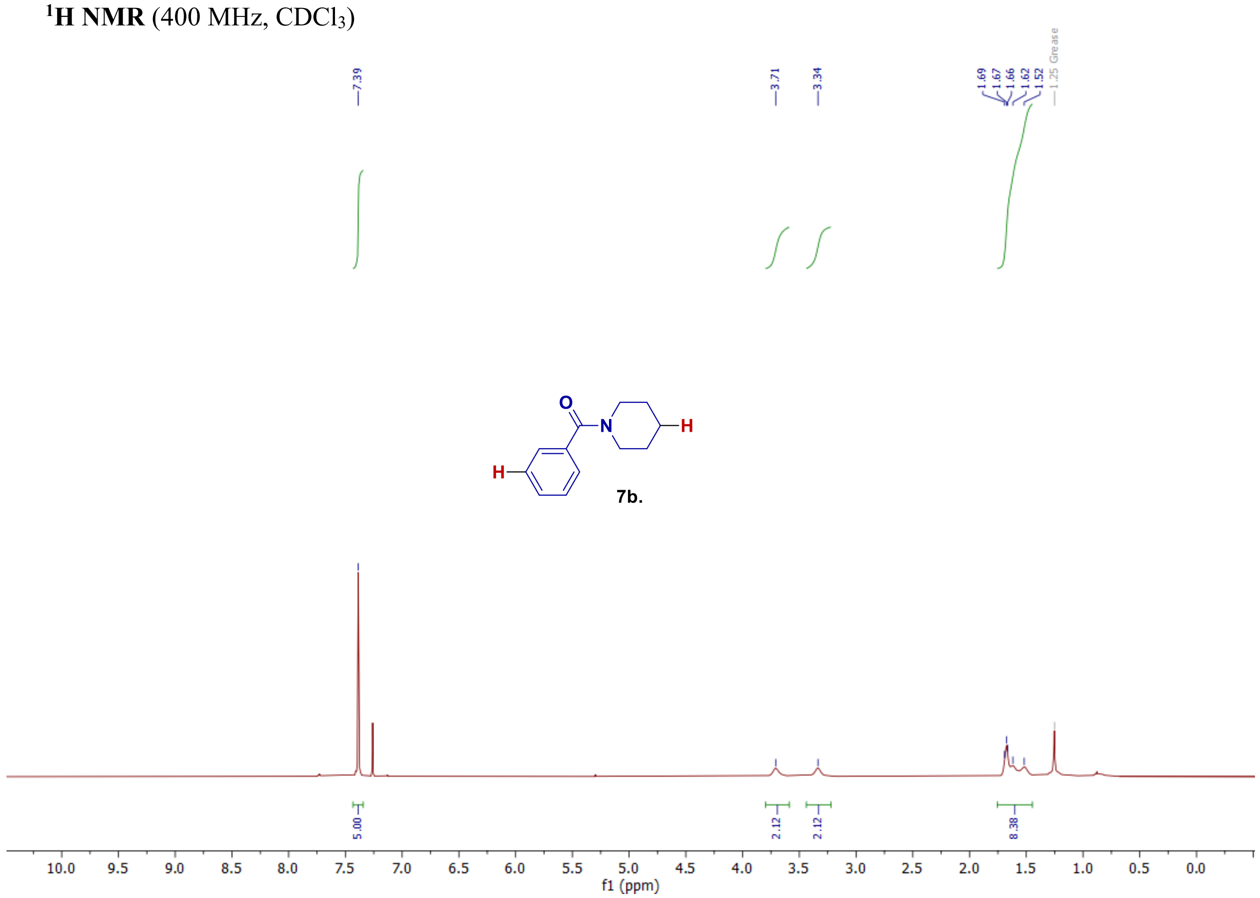

^{13}C NMR (101 MHz, $CDCl_3$)

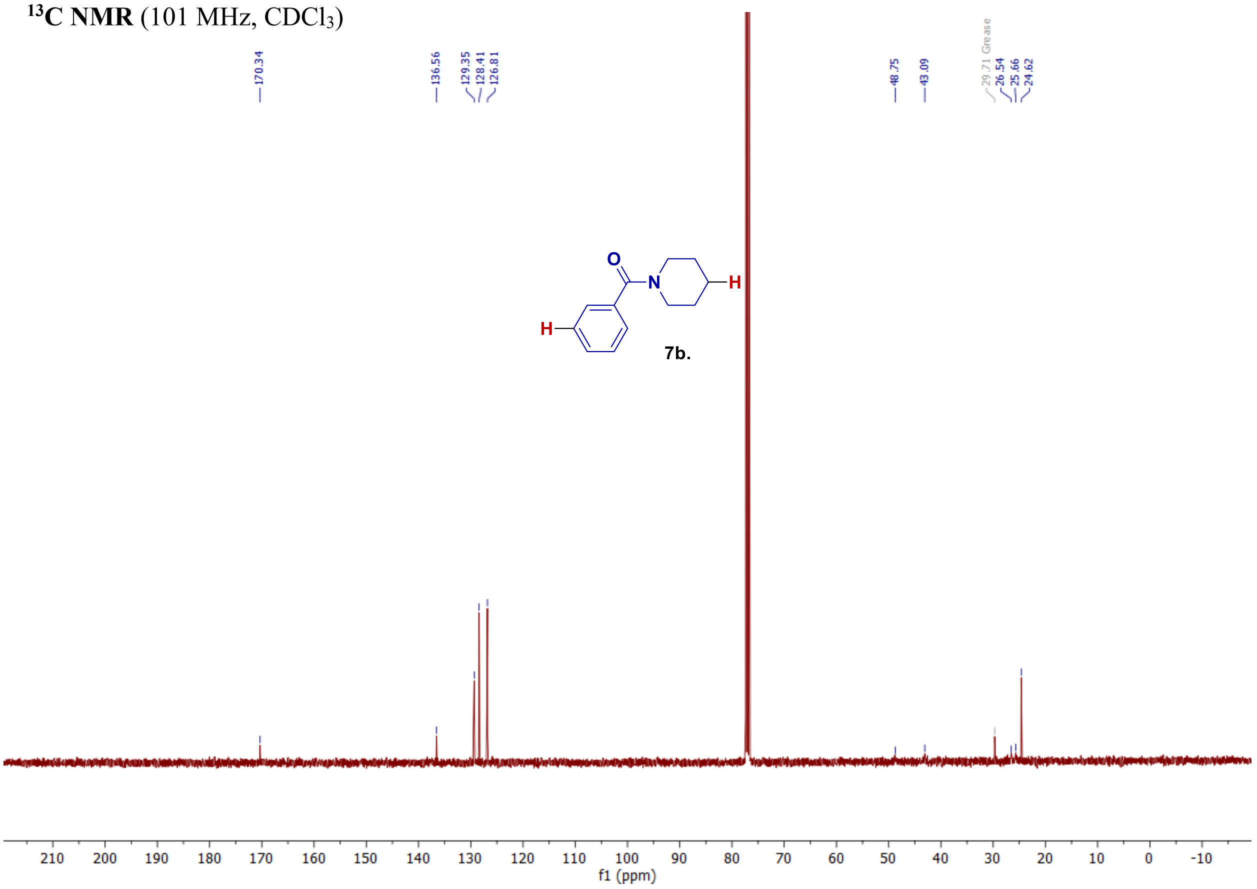

^{1}H NMR (400 MHz, $CDCl_3$)

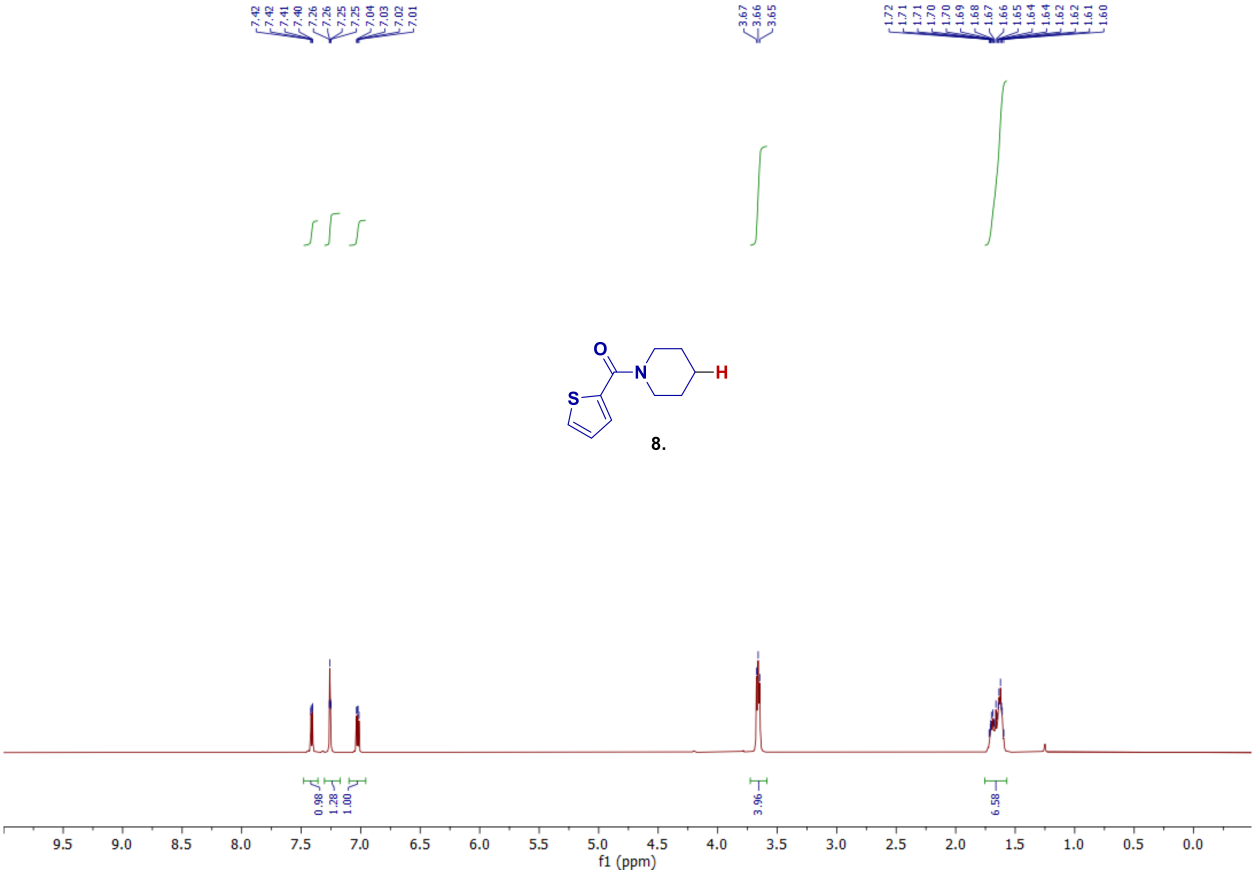

^{13}C NMR (101 MHz, $CDCl_3$)

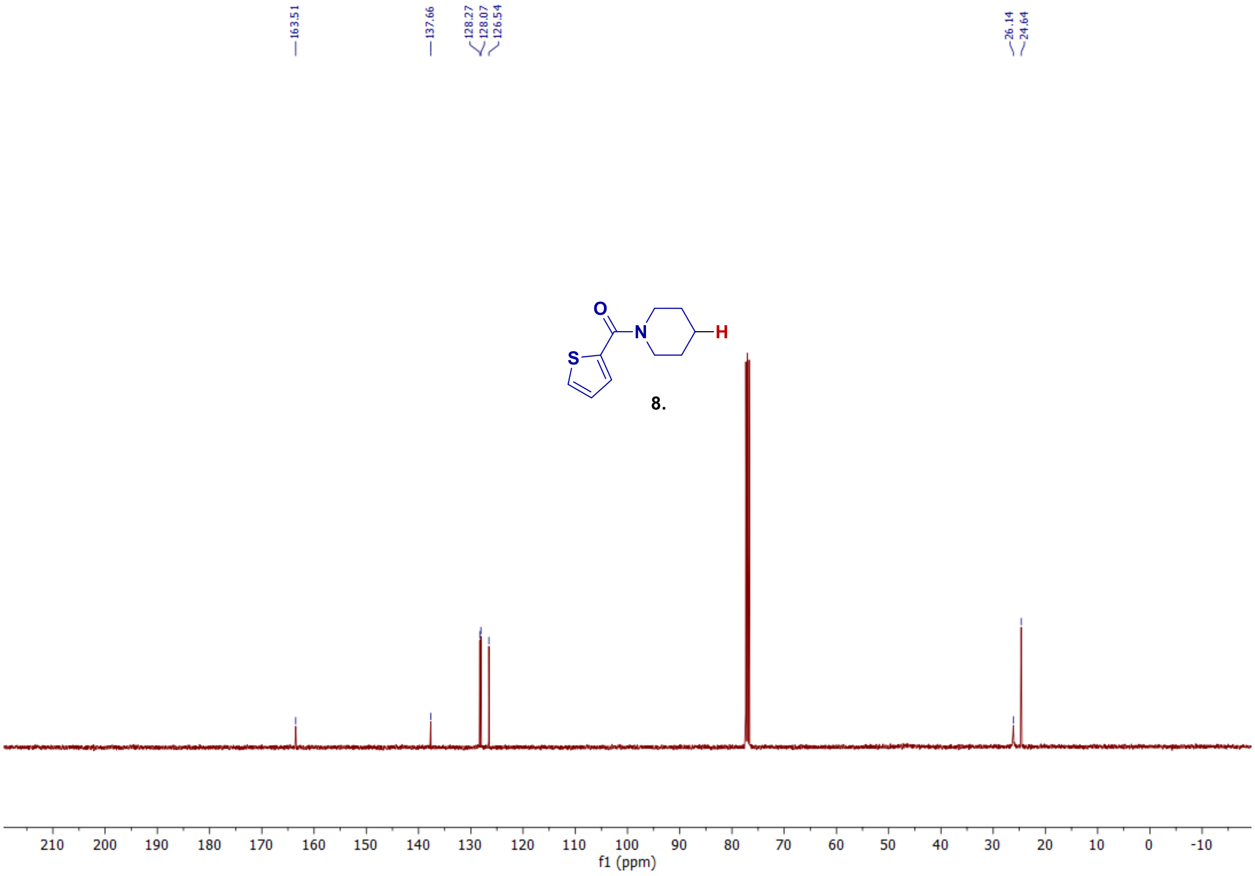

^{1}H NMR (400 MHz, $CDCl_3$)

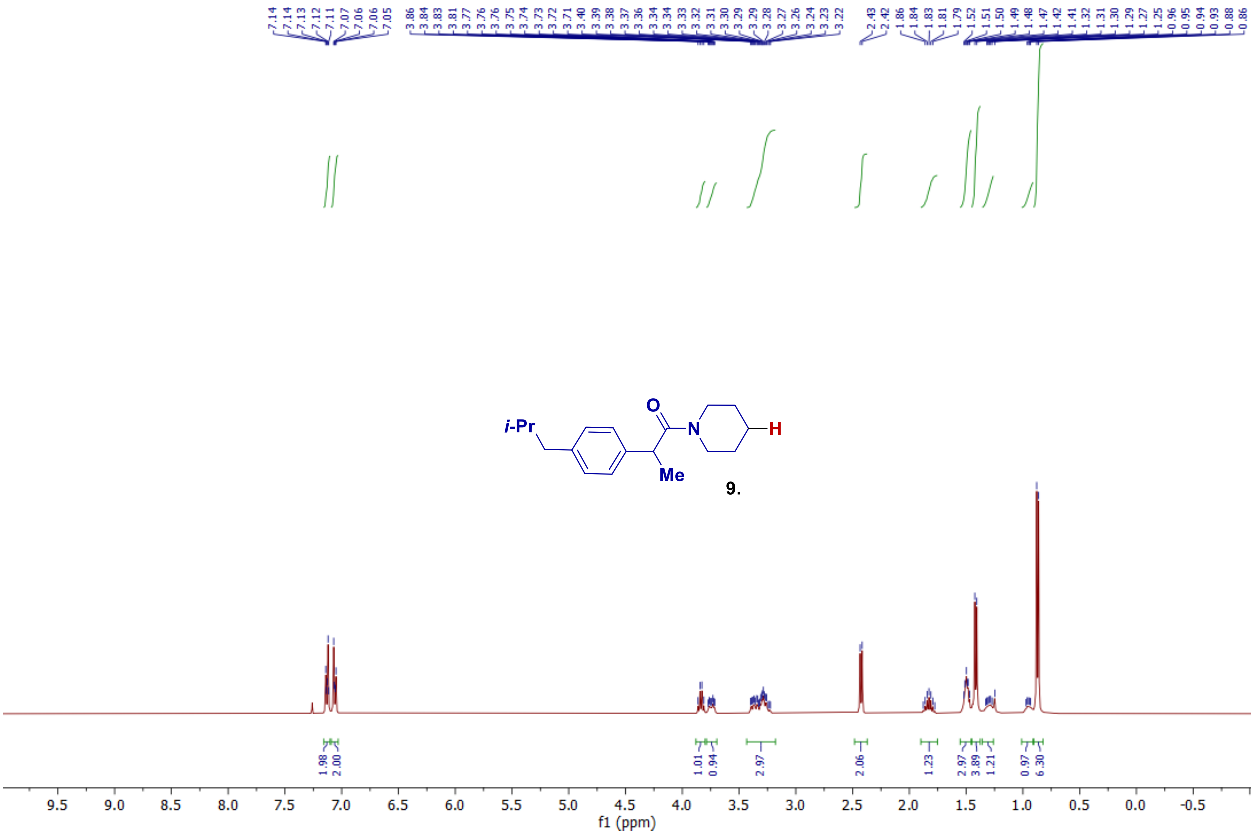

^{13}C NMR (101 MHz, $CDCl_3$)

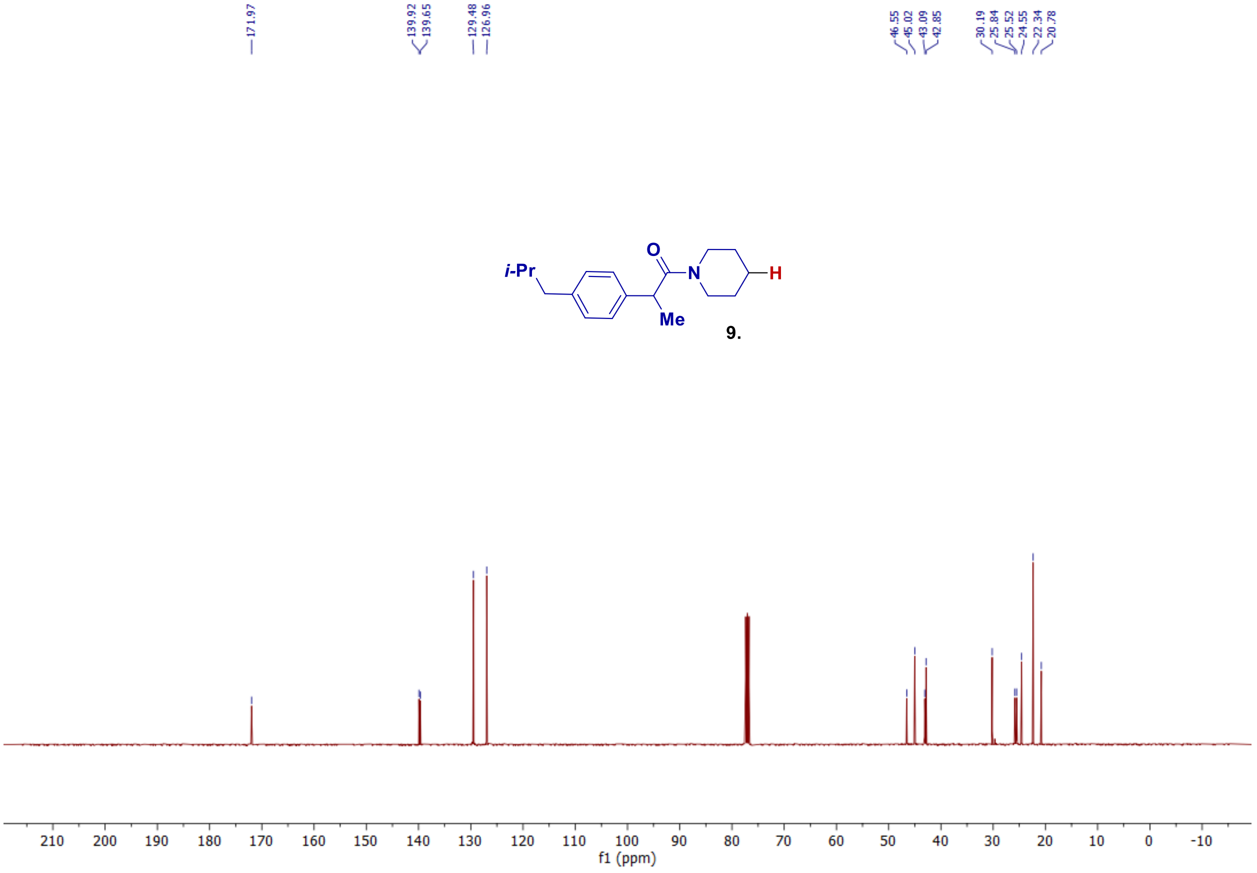

^{1}H NMR (400 MHz, $CDCl_3$)

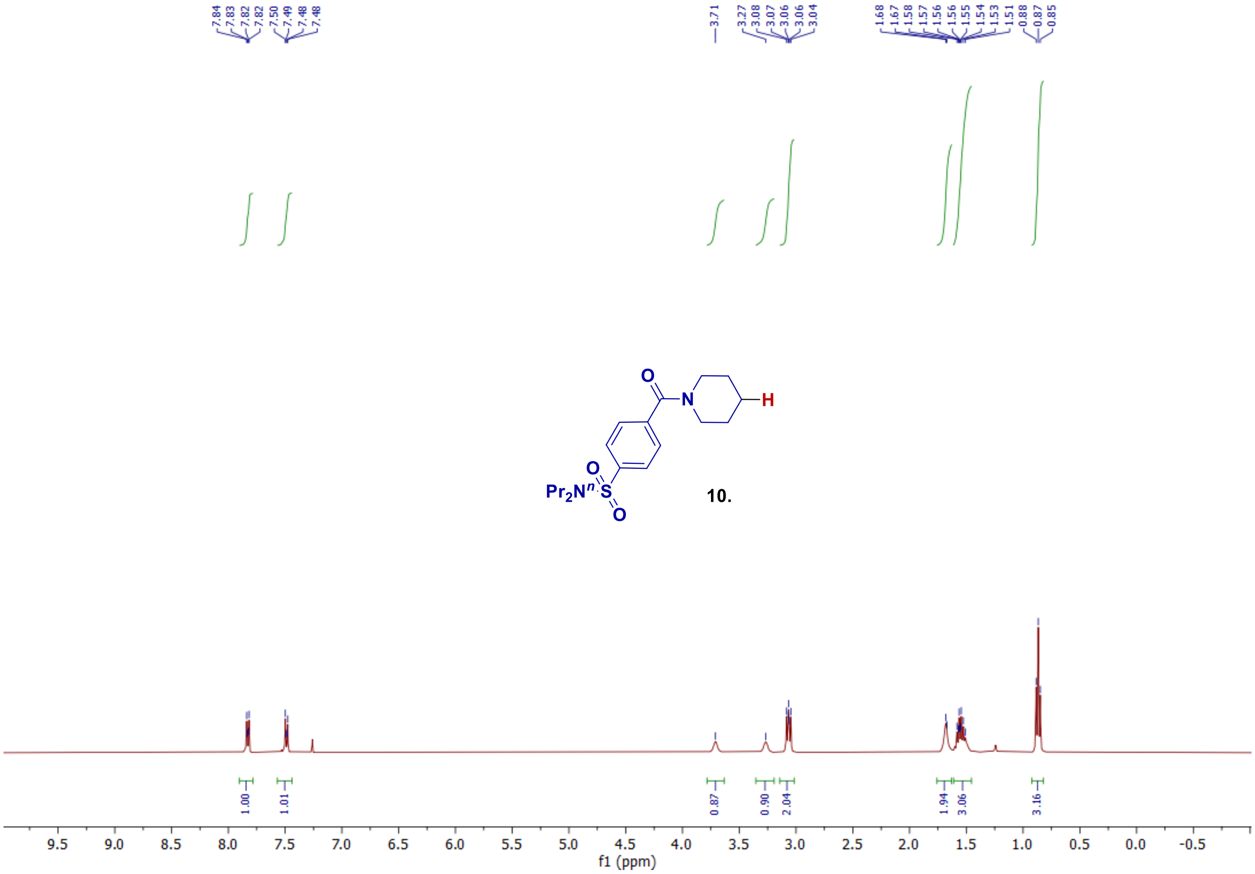

^{13}C NMR (101 MHz, $CDCl_3$)

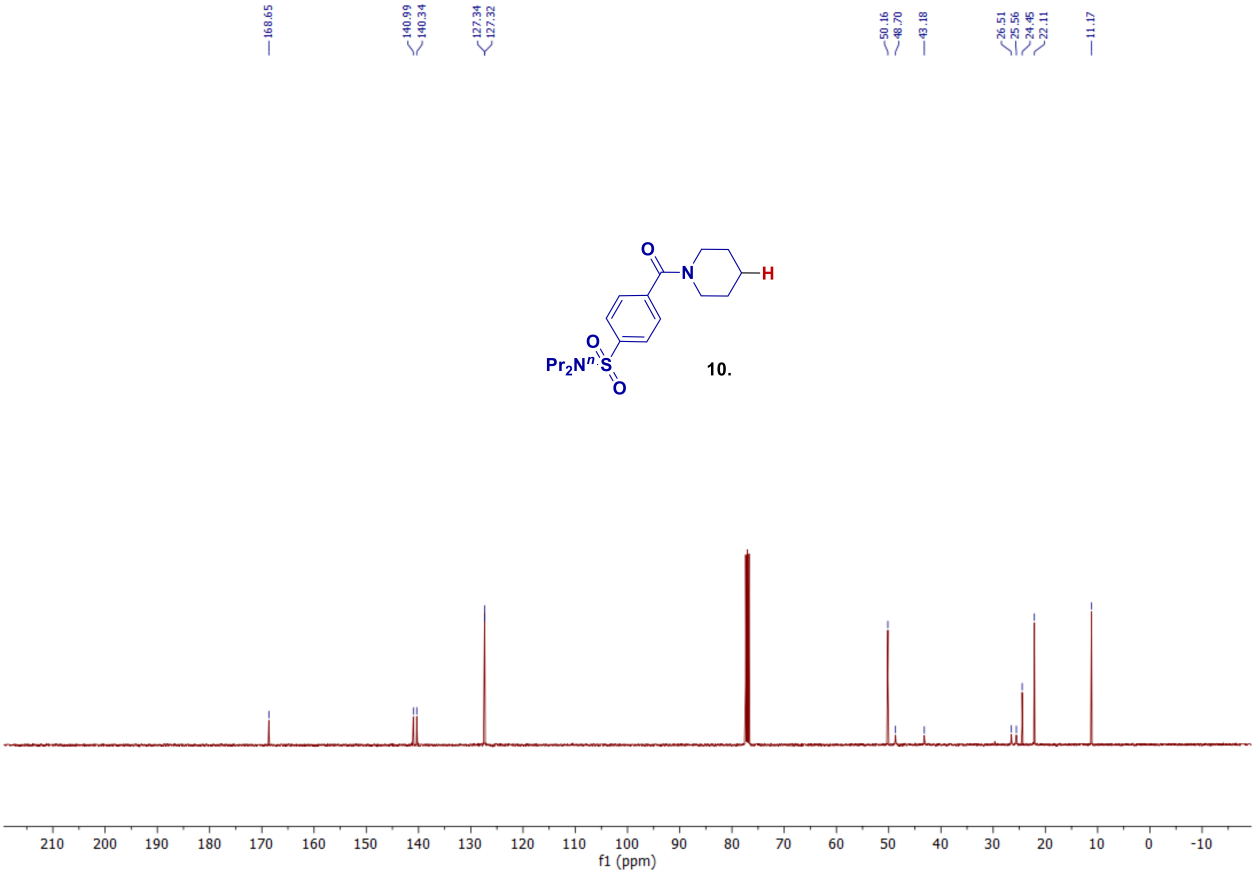

^{1}H NMR (400 MHz, $CDCl_3$)

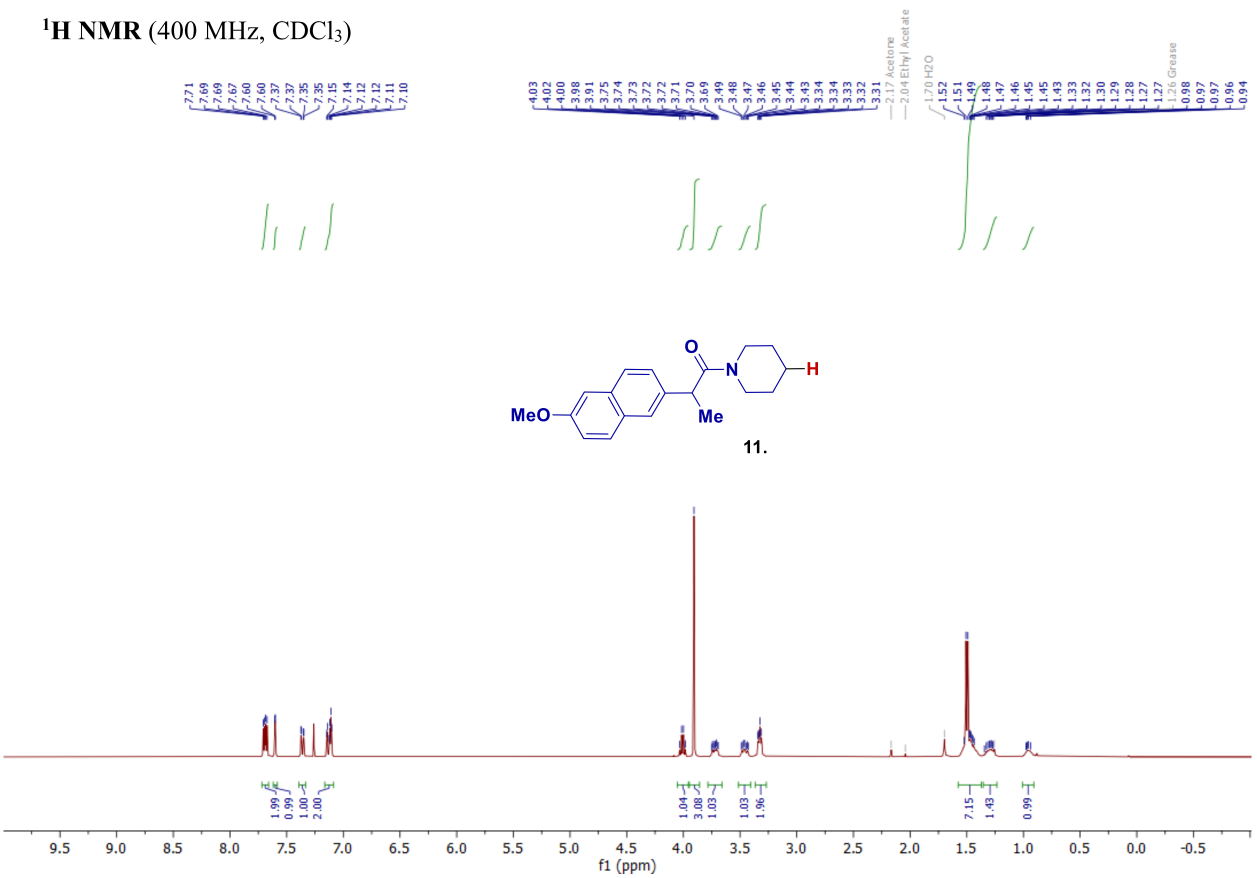

11.

^{13}C NMR (101 MHz, $CDCl_3$)

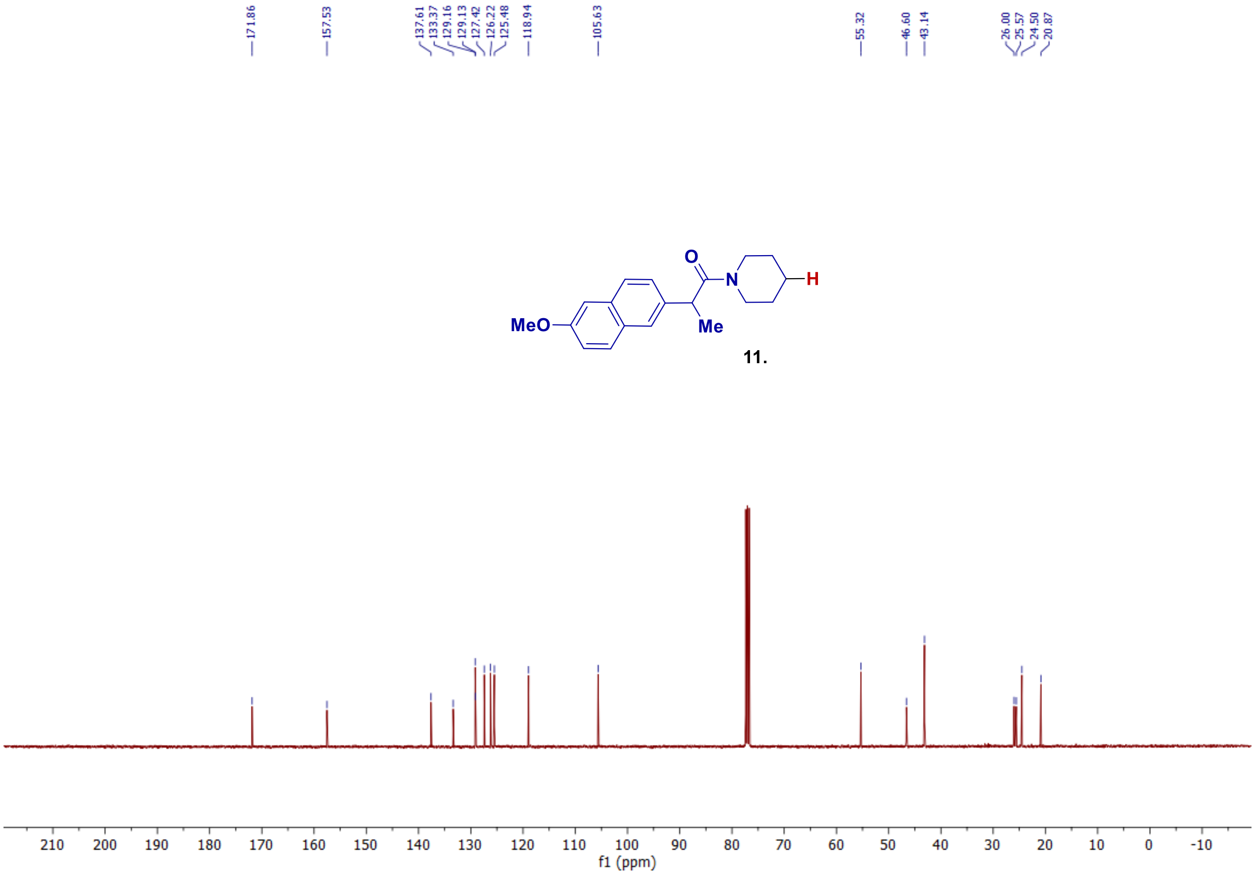

11.

^{1}H NMR (400 MHz, $CDCl_3$)

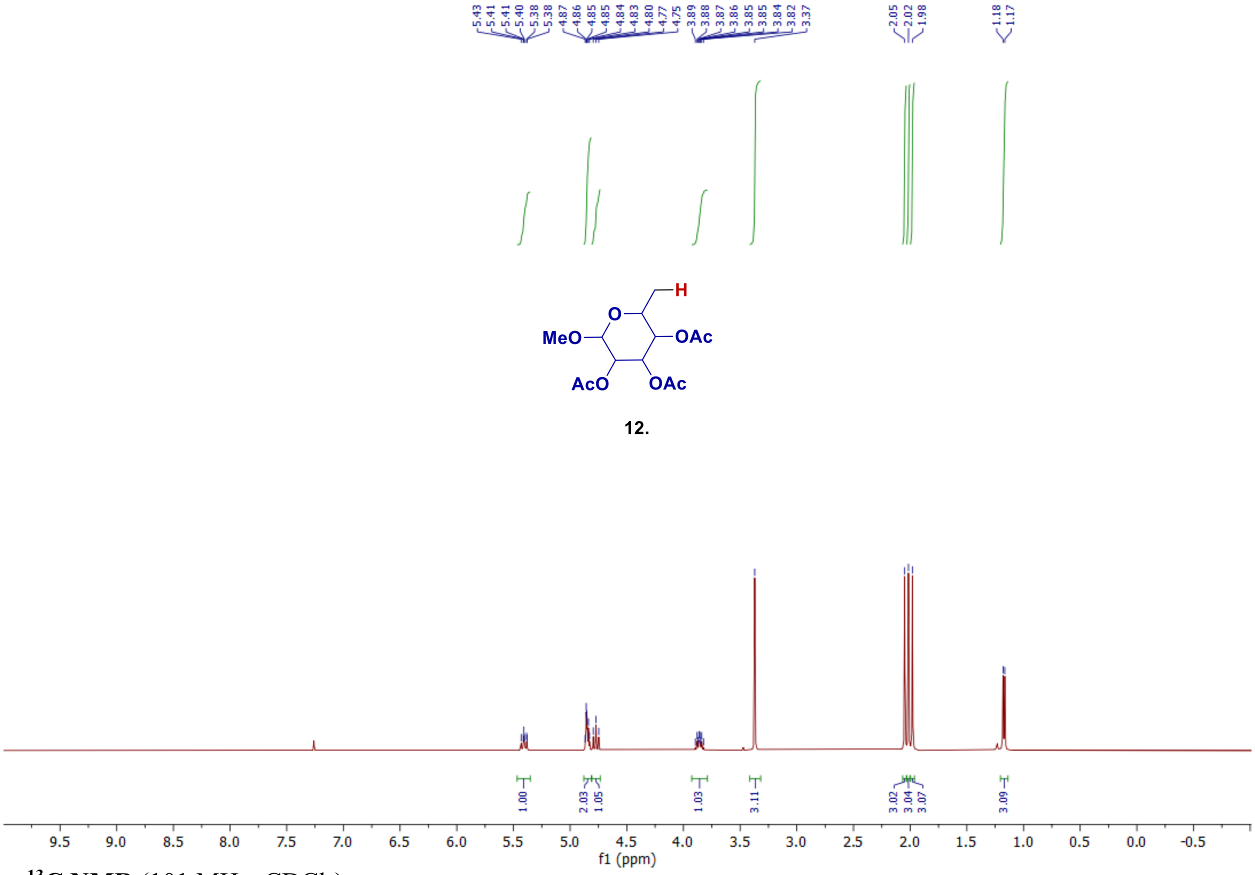

^{13}C NMR (101 MHz, $CDCl_3$)

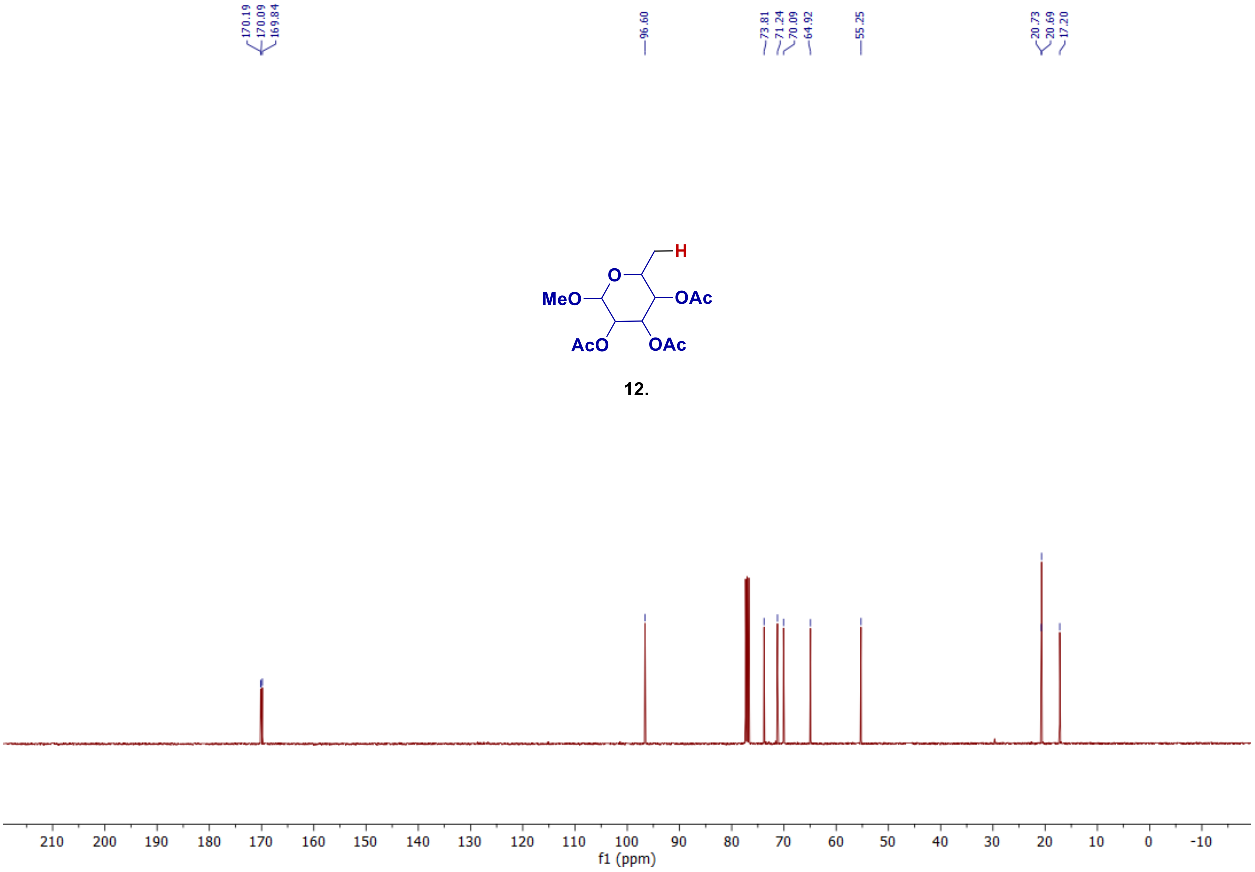

^{1}H NMR (400 MHz, $CDCl_3$)

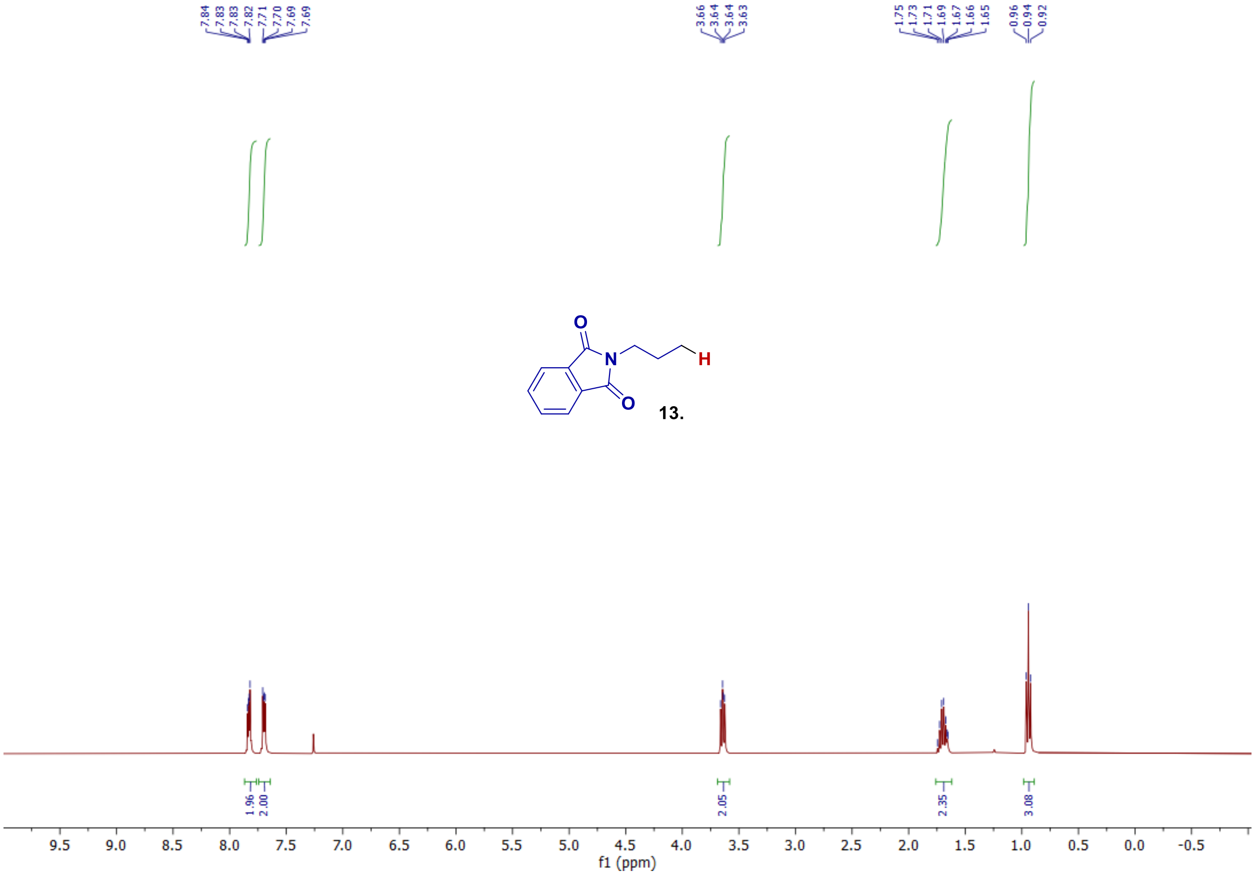

^{13}C NMR (101 MHz, $CDCl_3$)

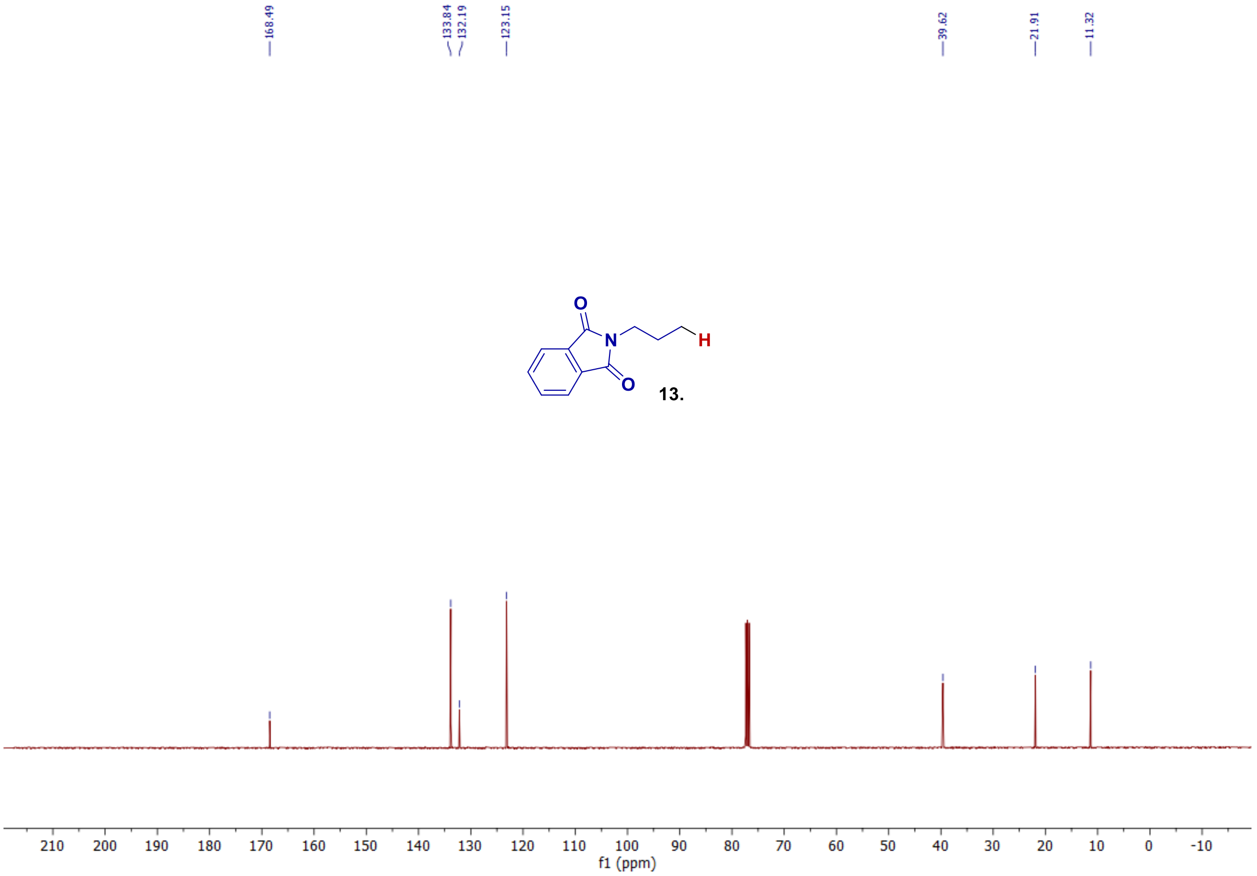

^{1}H NMR (400 MHz, $CDCl_3$)

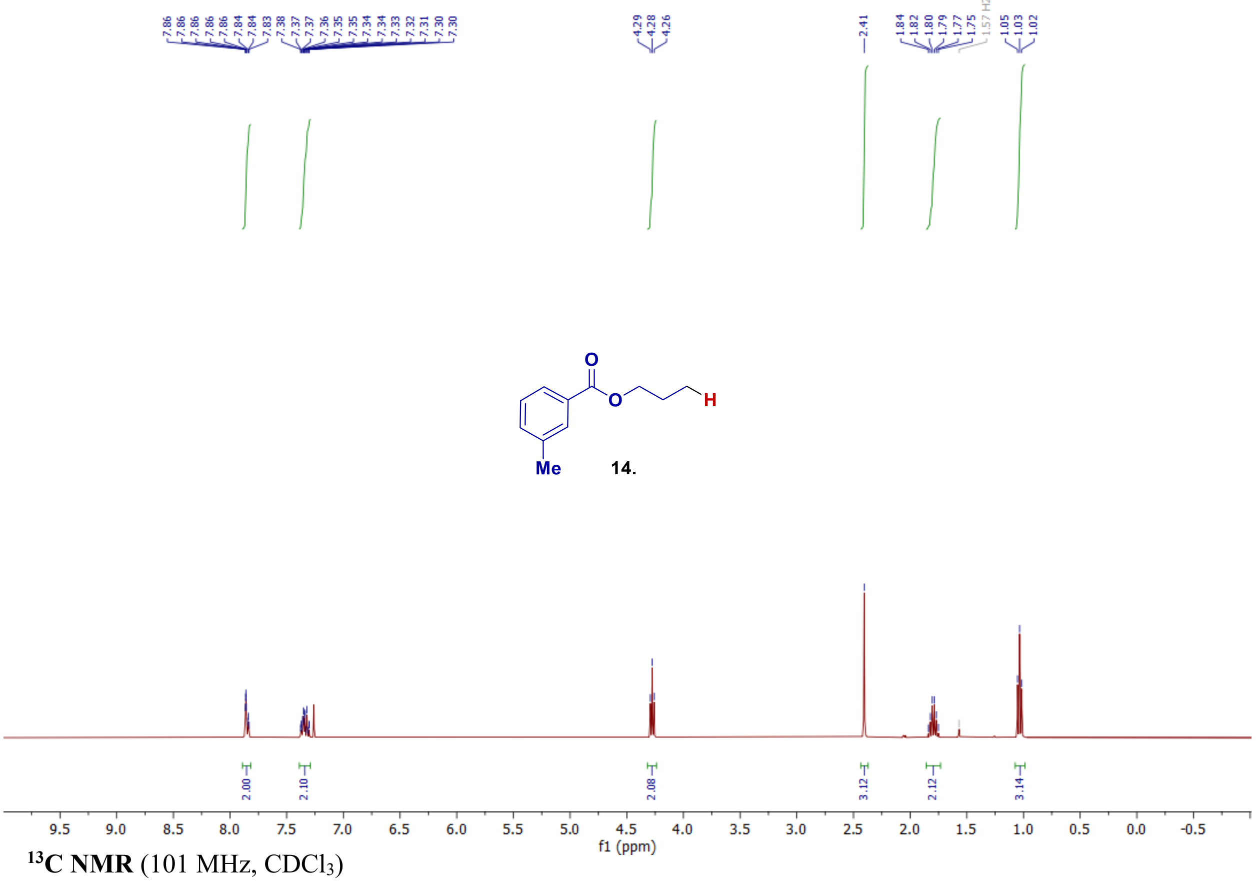

^{13}C NMR (101 MHz, $CDCl_3$)

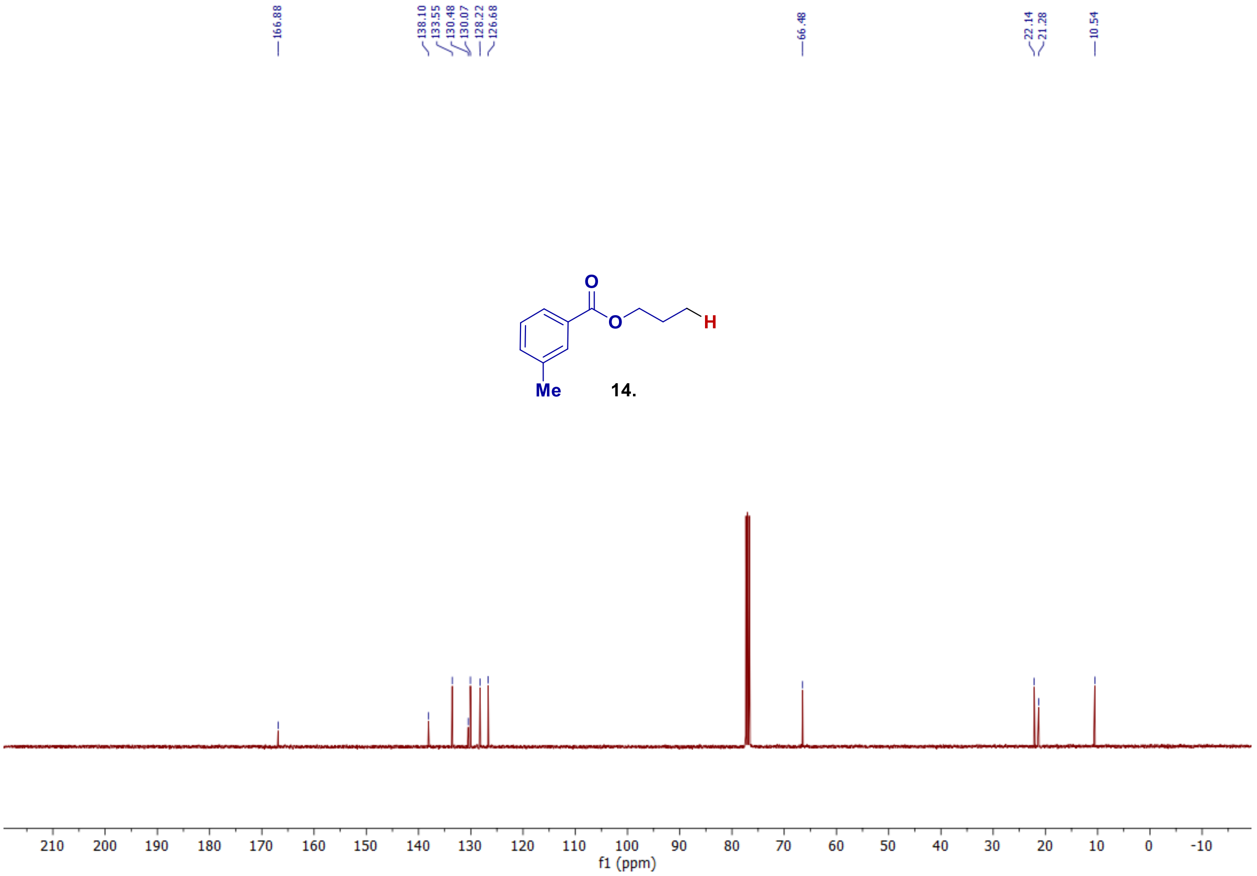

^{1}H NMR (400 MHz, $CDCl_3$)

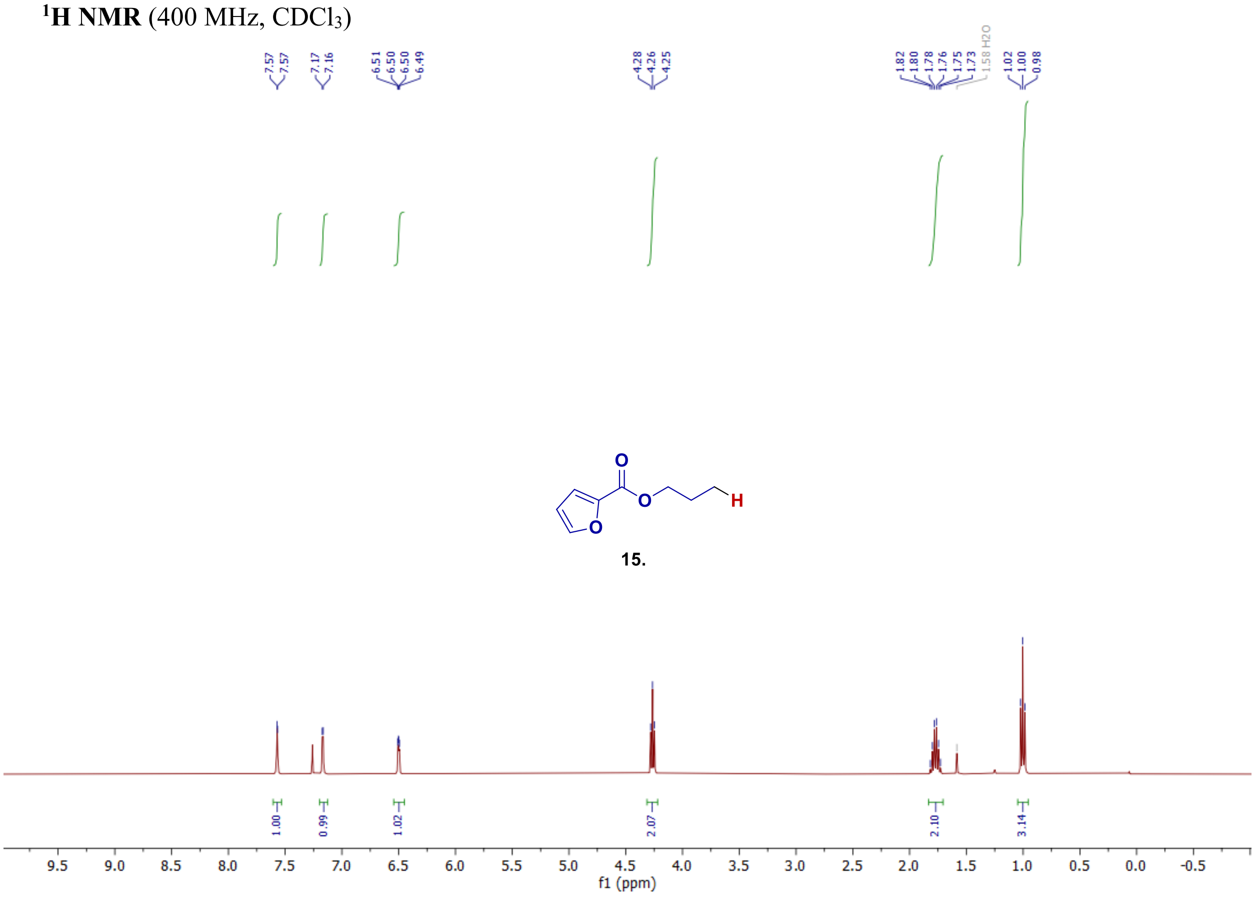

15.

^{13}C NMR (101 MHz, $CDCl_3$)

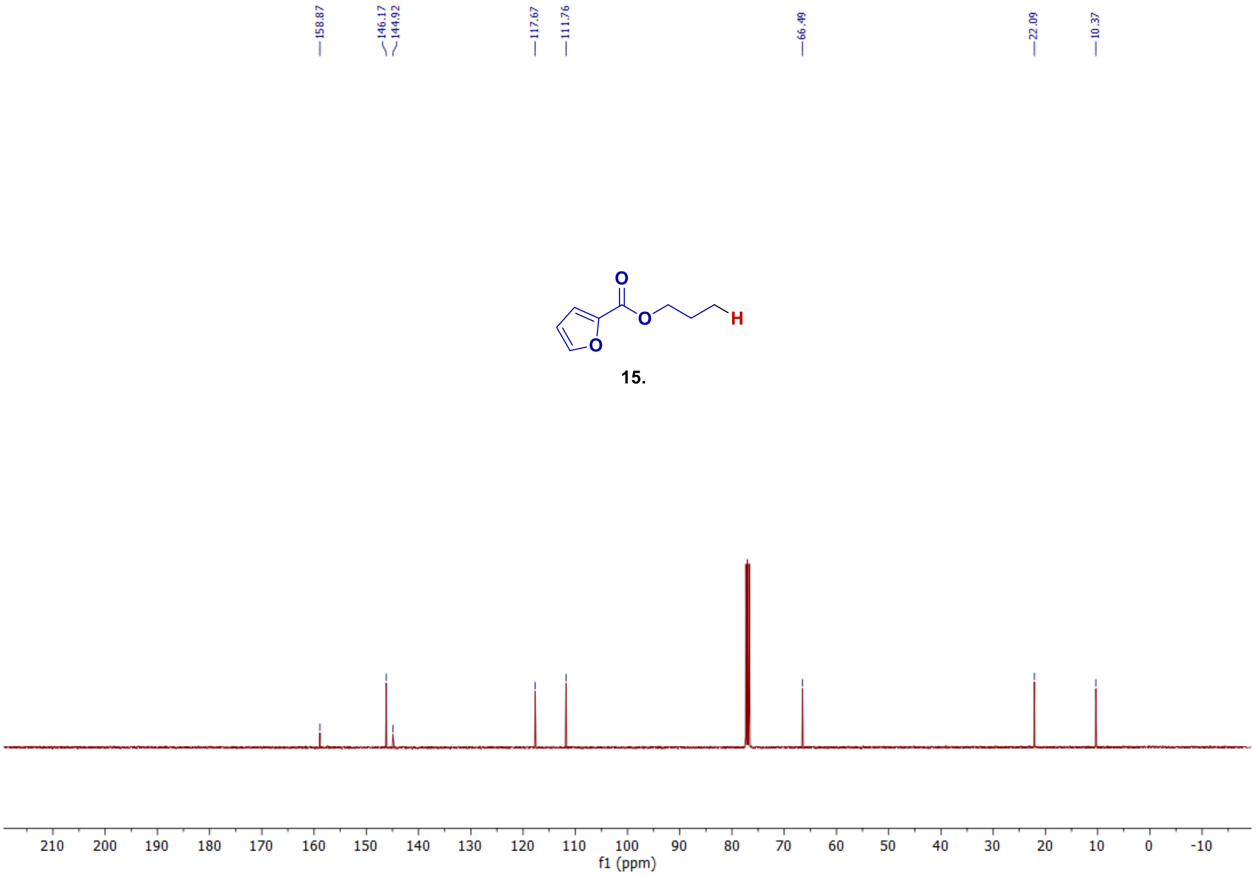

15.

^{1}H NMR (400 MHz, $CDCl_3$)

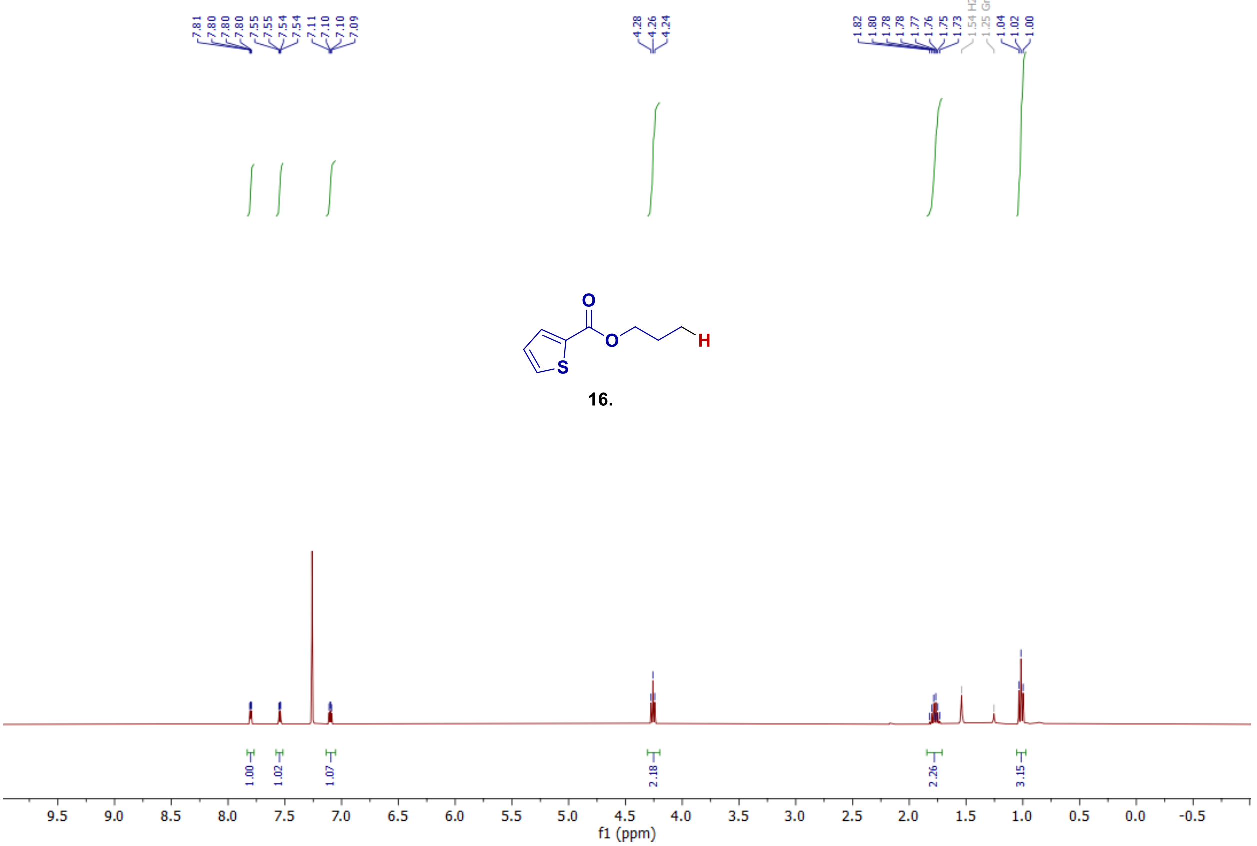

16.

^{13}C NMR (101 MHz, $CDCl_3$)

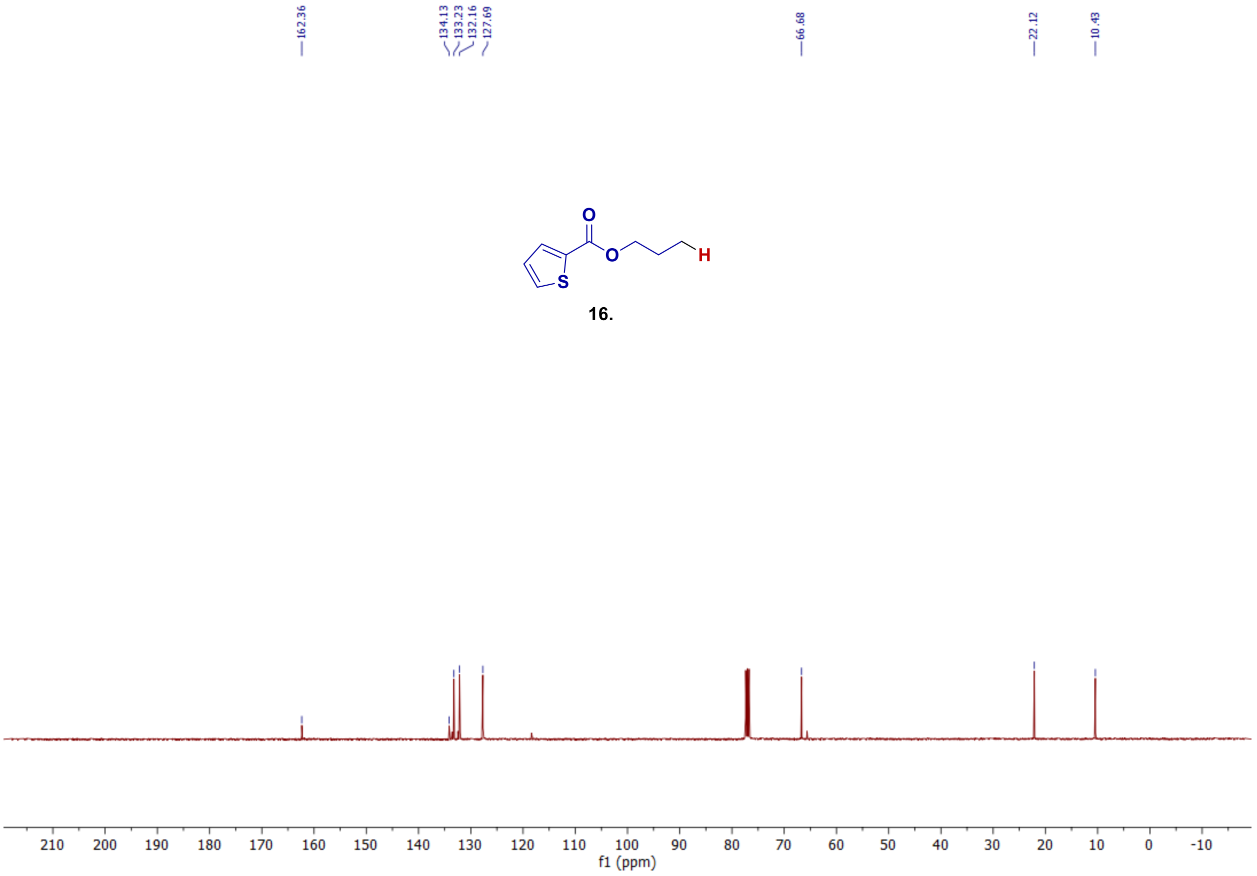

16.

^{1}H NMR (400 MHz, $CDCl_3$)

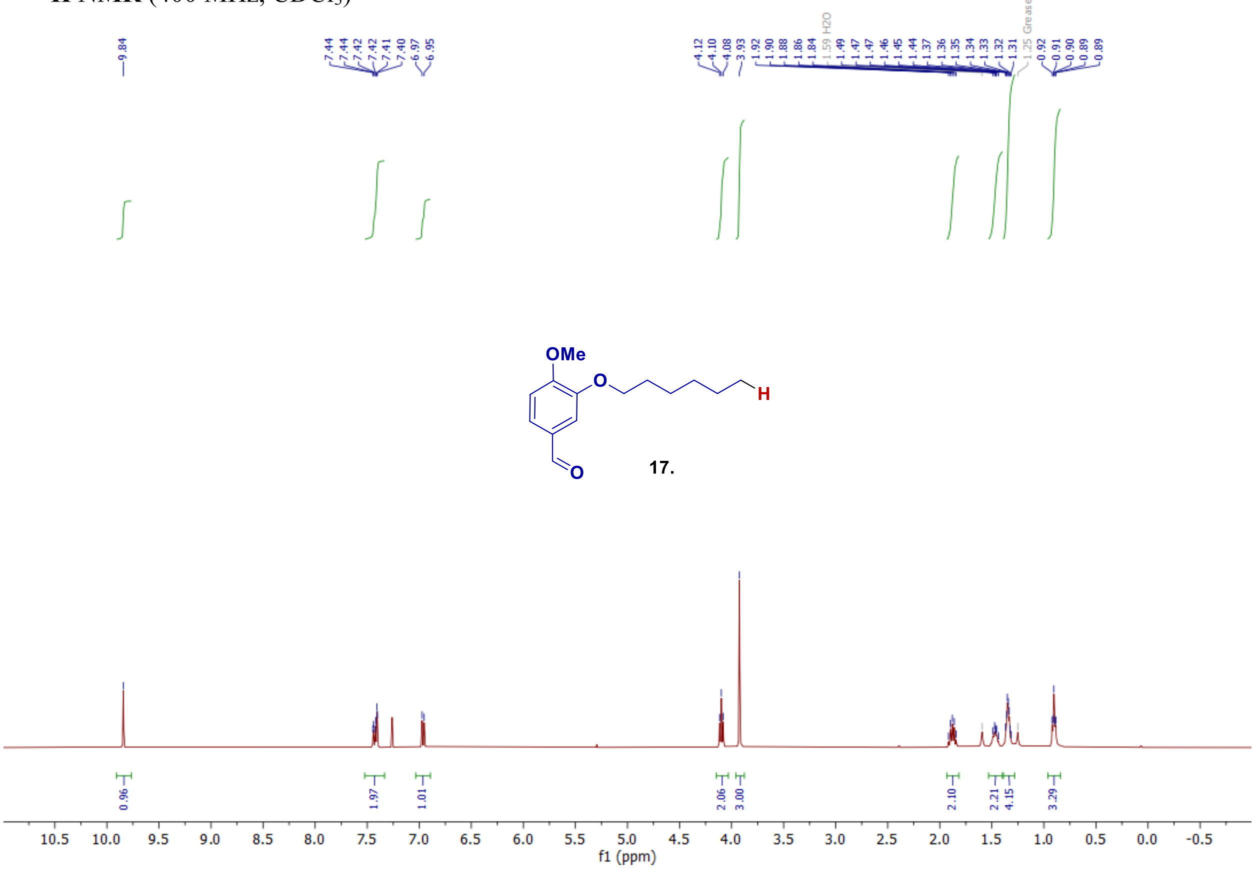

^{13}C NMR (101 MHz, $CDCl_3$)

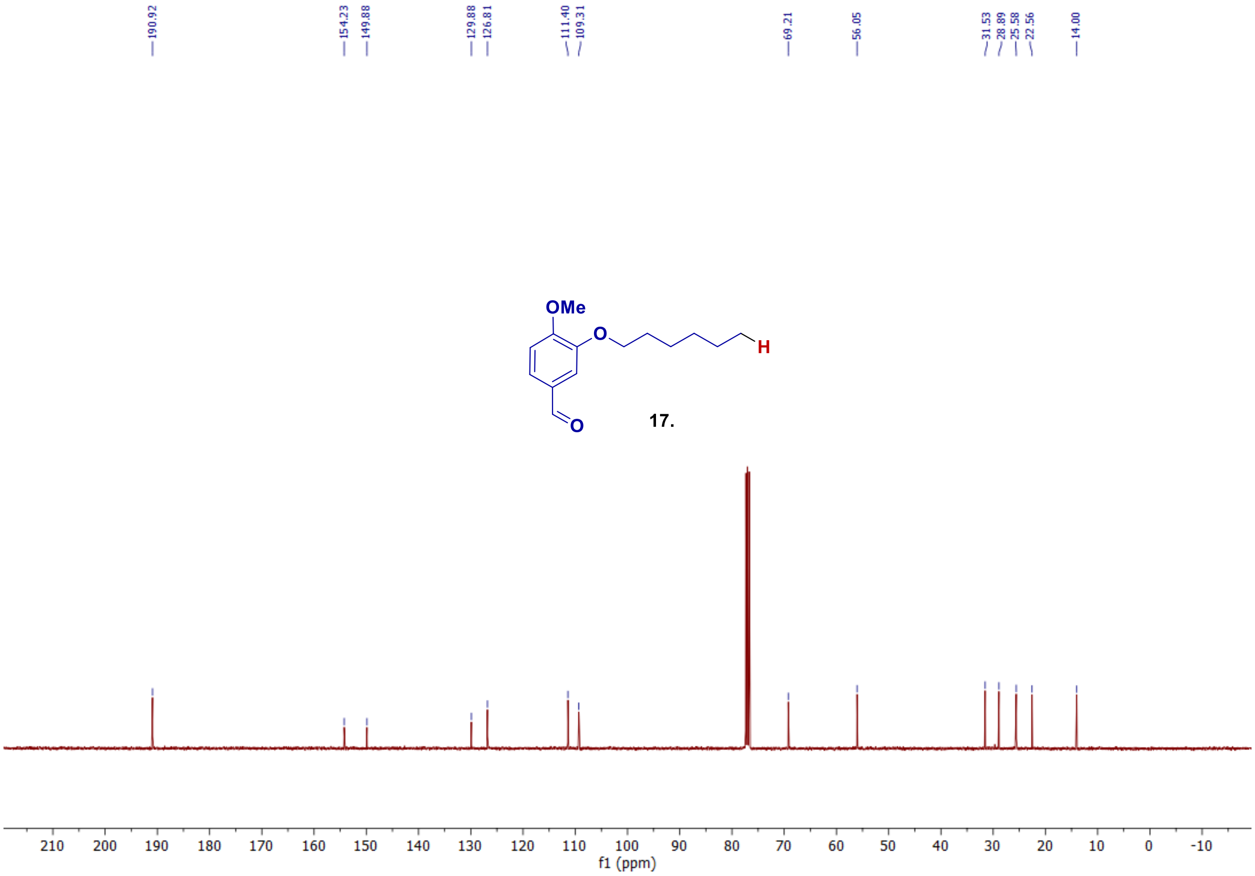

^{1}**H NMR** (400 MHz, $CDCl_3$)

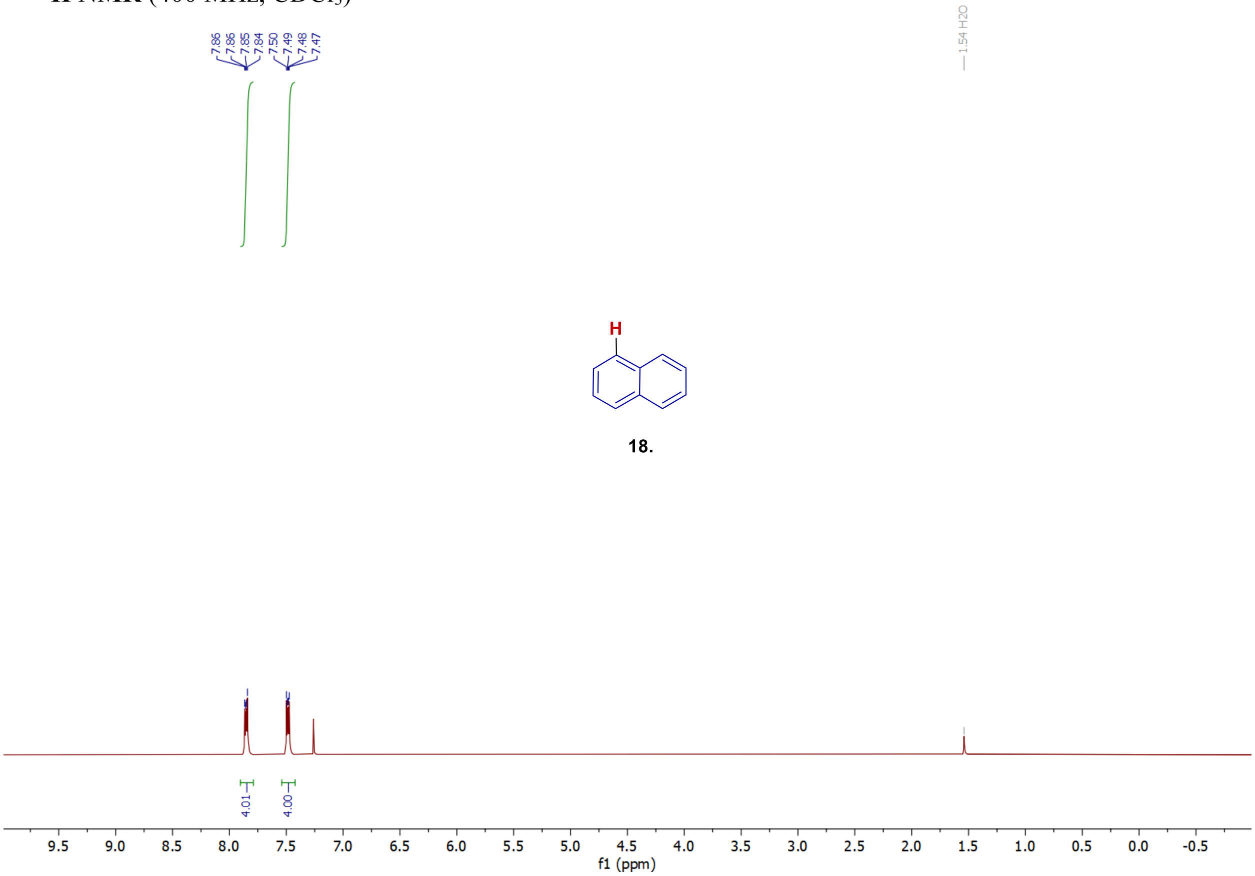

^{13}**C NMR** (101 MHz, $CDCl_3$)

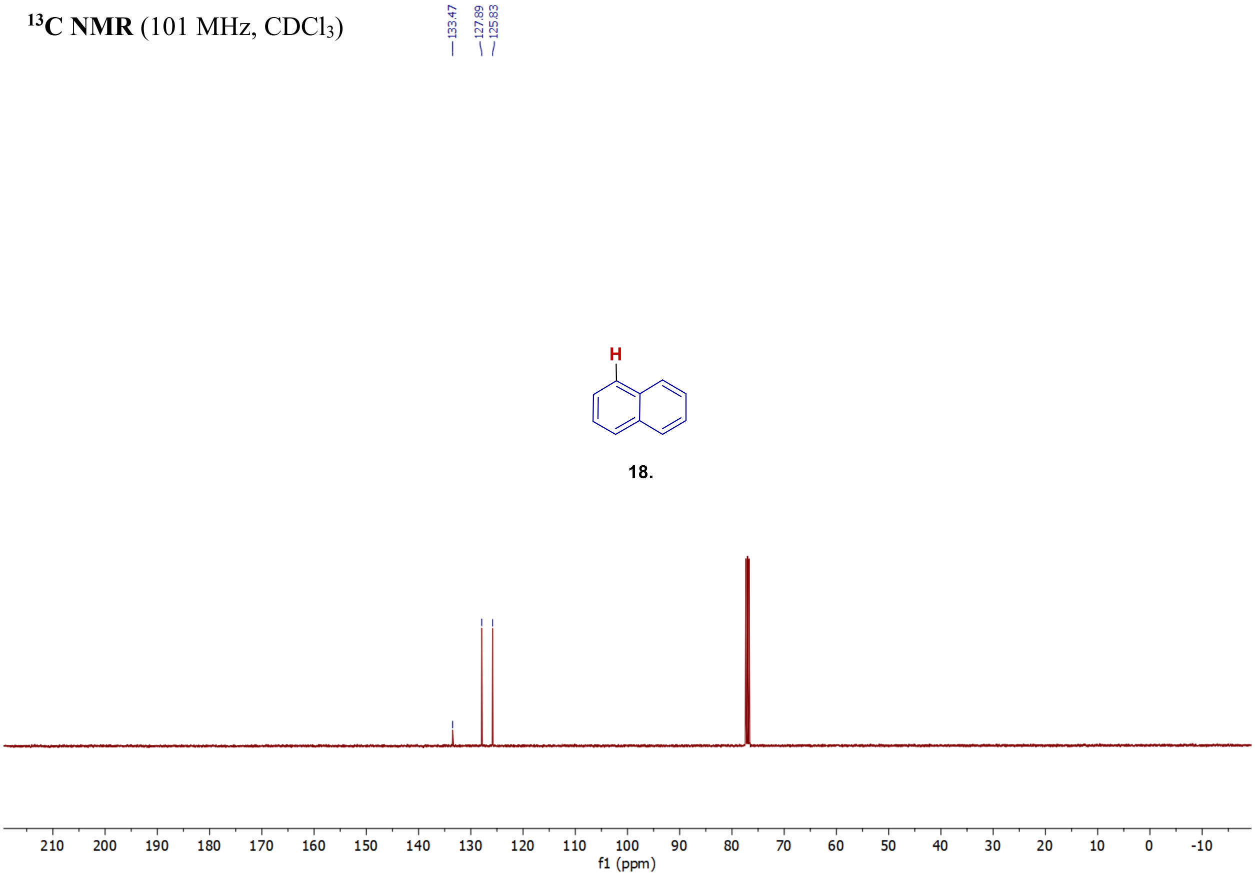

^{1}H NMR (400 MHz, $CDCl_3$)

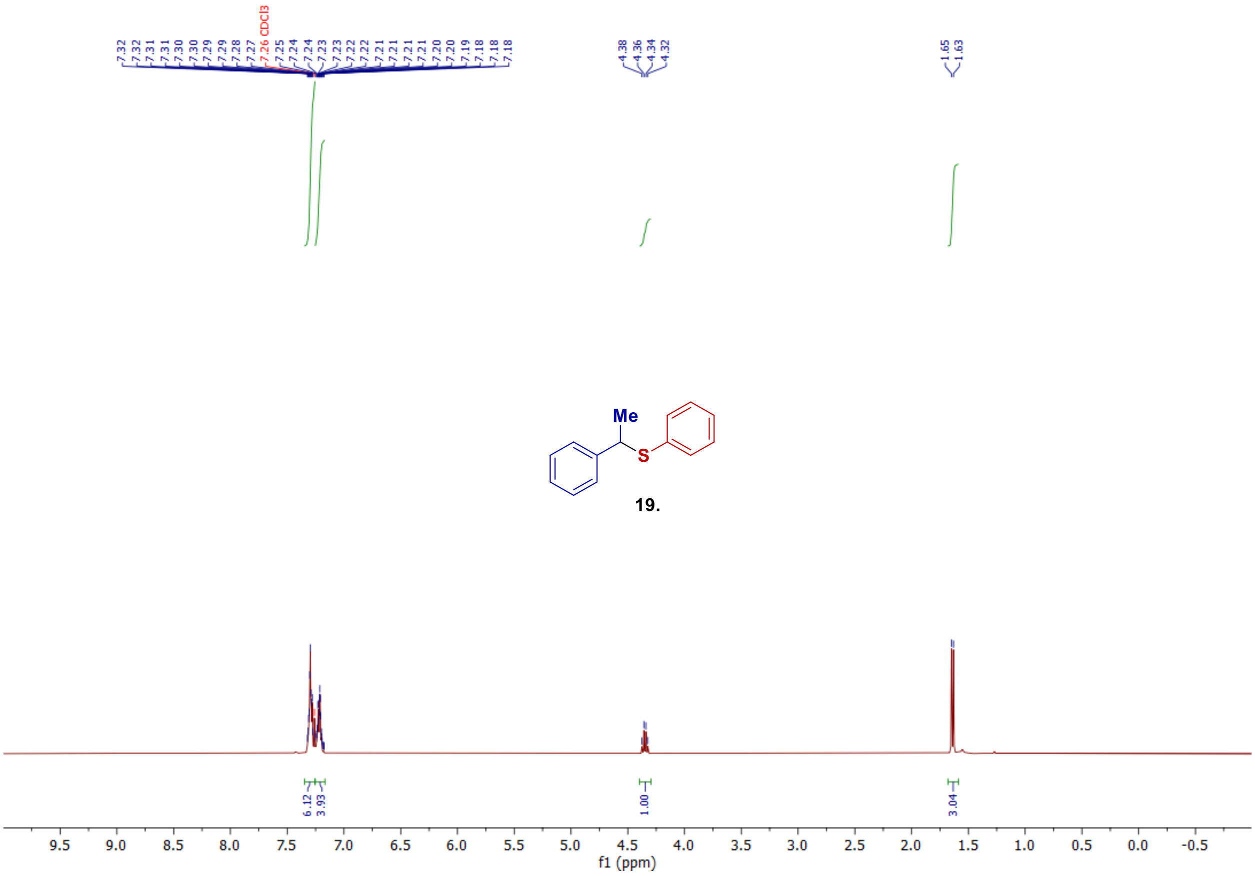

^{13}C NMR (101 MHz, $CDCl_3$)

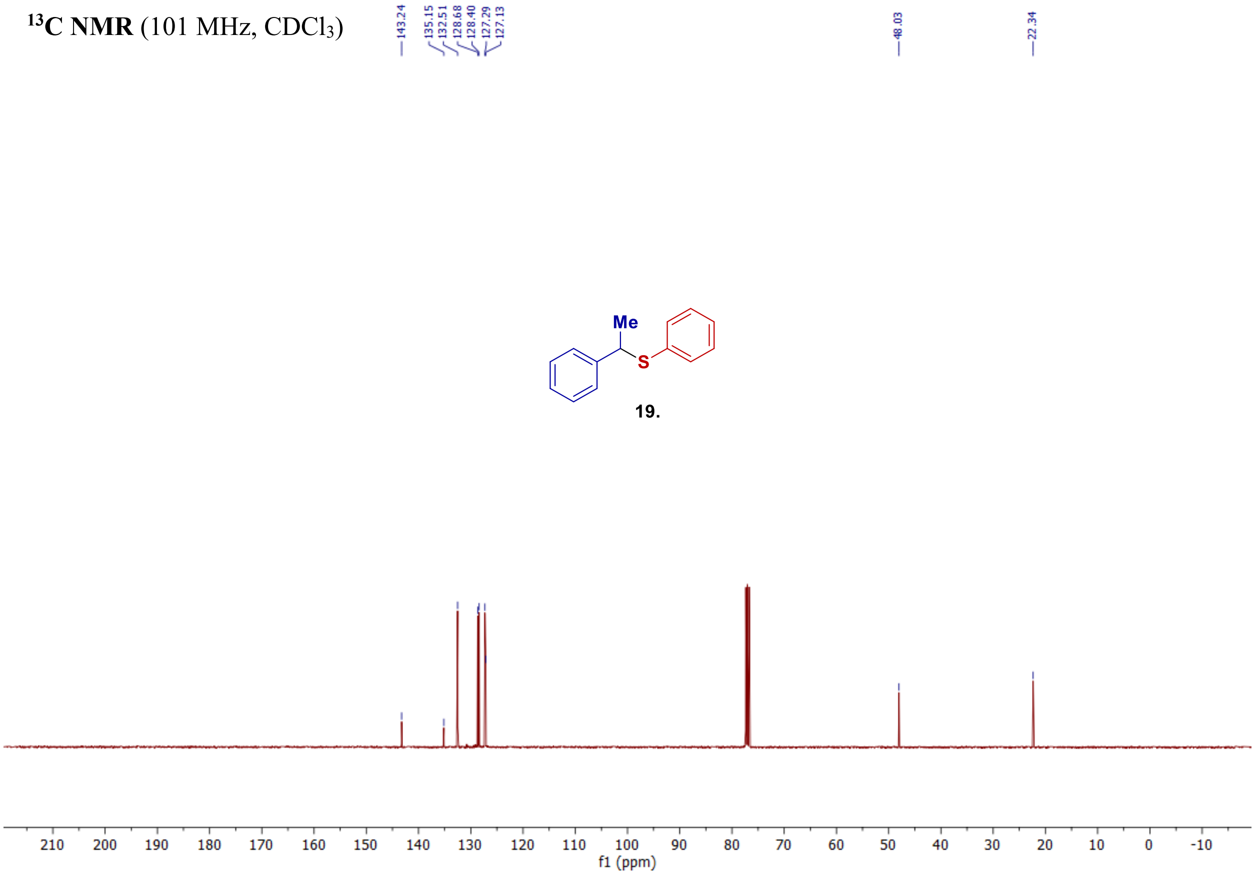

^{1}H NMR (400 MHz, $CDCl_3$)

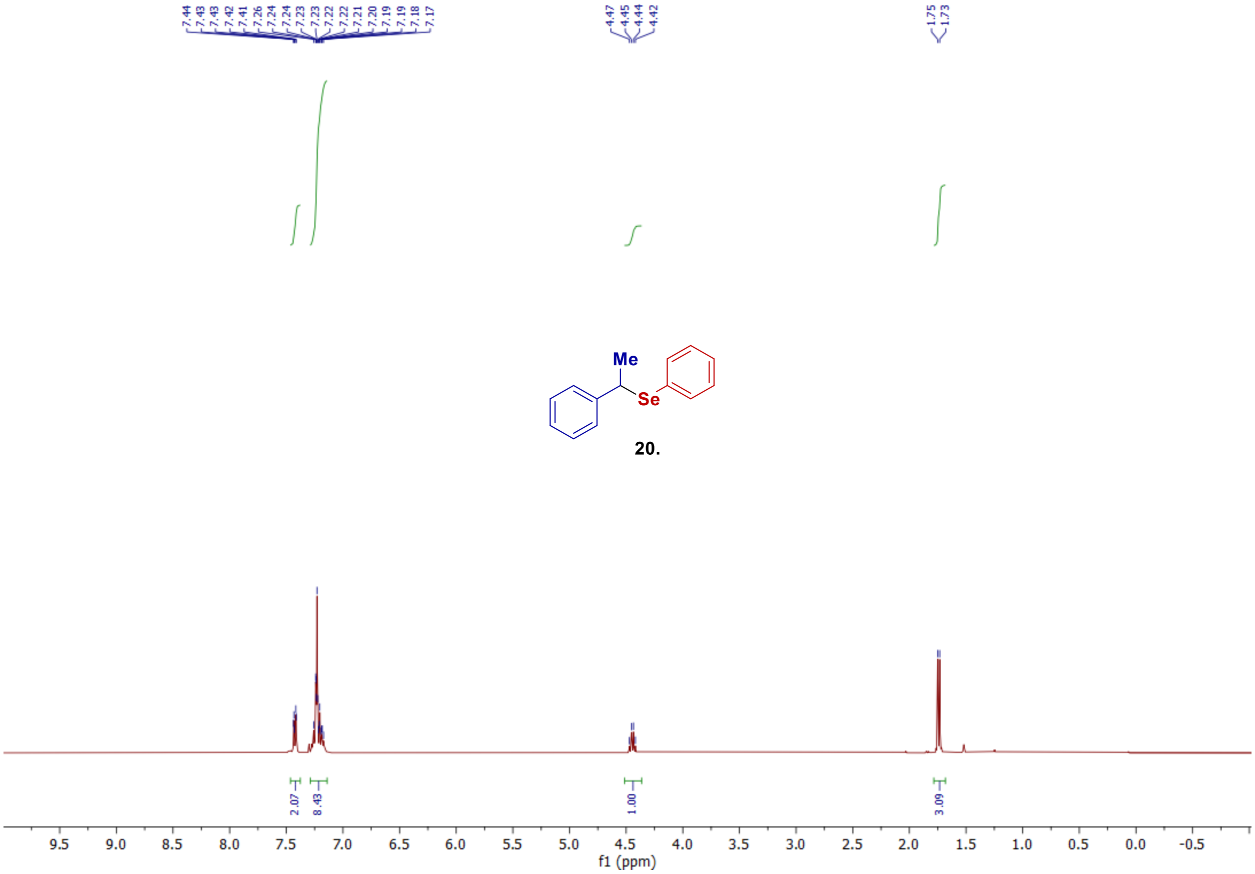

^{13}C NMR (101 MHz, $CDCl_3$)

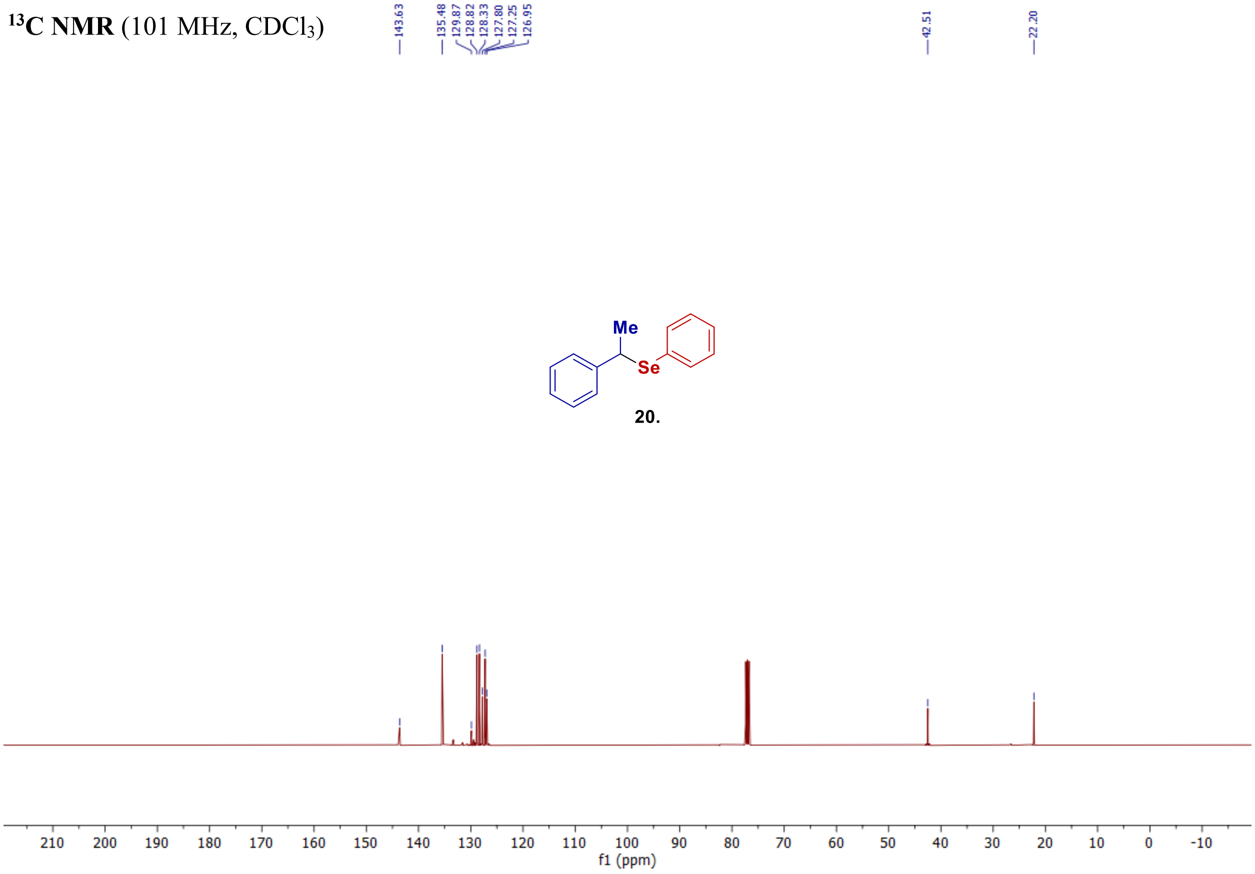

^{1}H NMR (400 MHz, $CDCl_3$)

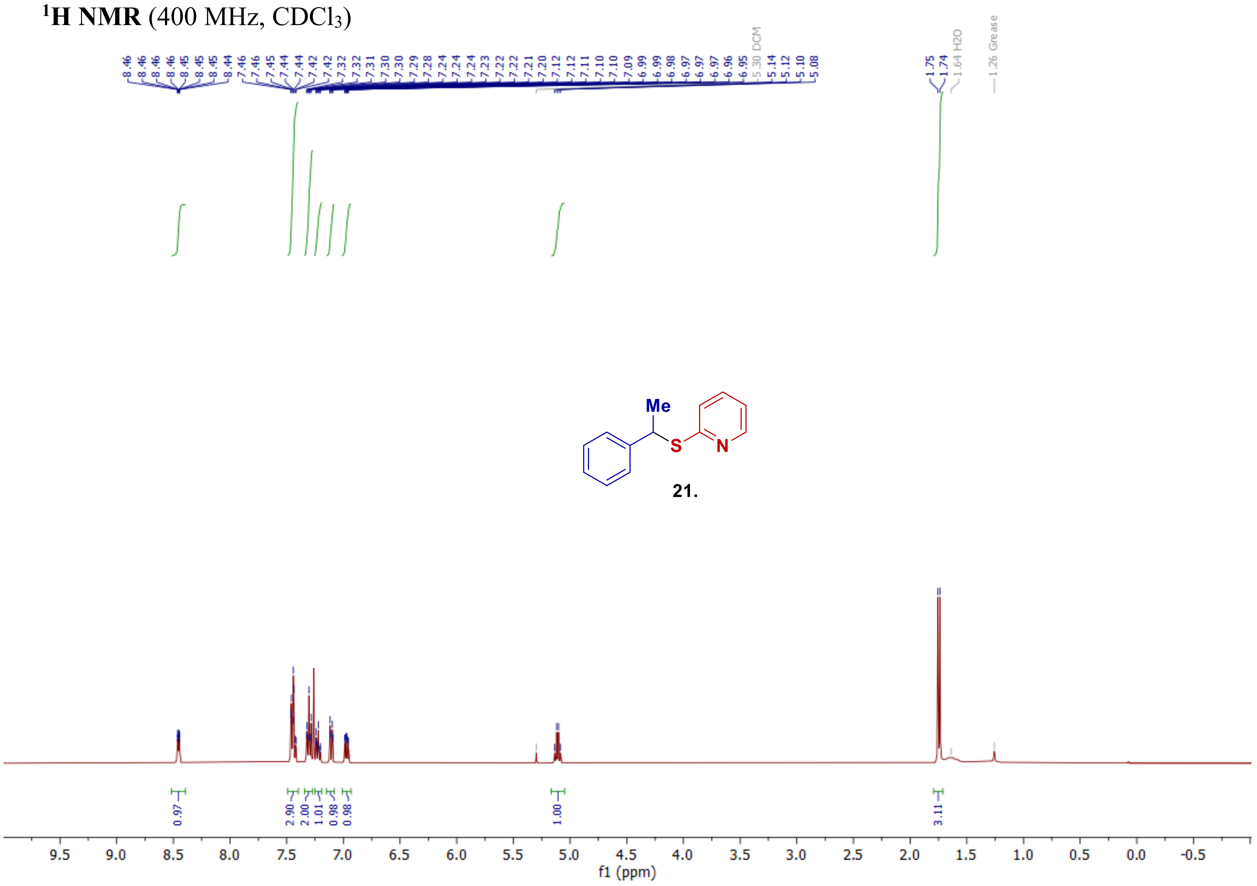

^{13}C NMR (101 MHz, $CDCl_3$)

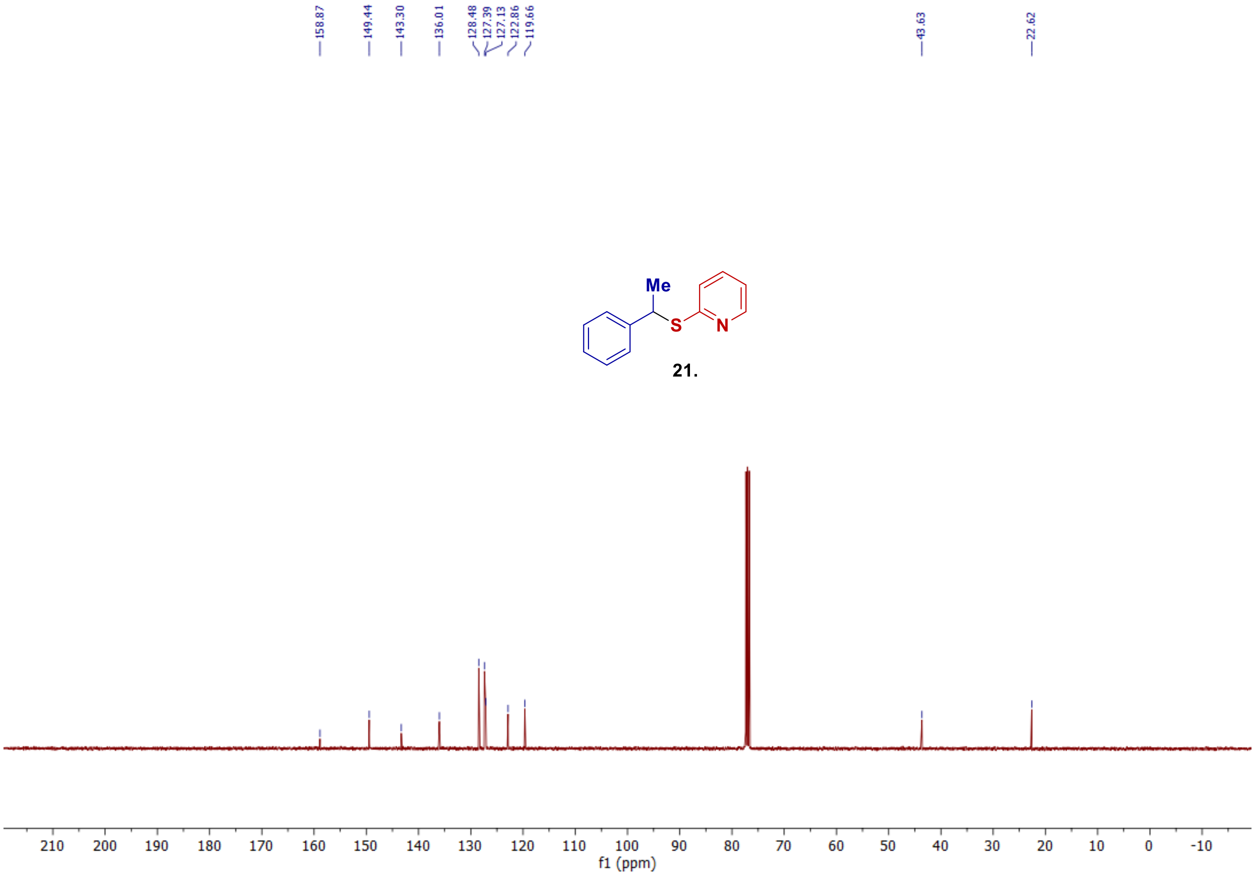

^{1}H NMR (400 MHz, $CDCl_3$)

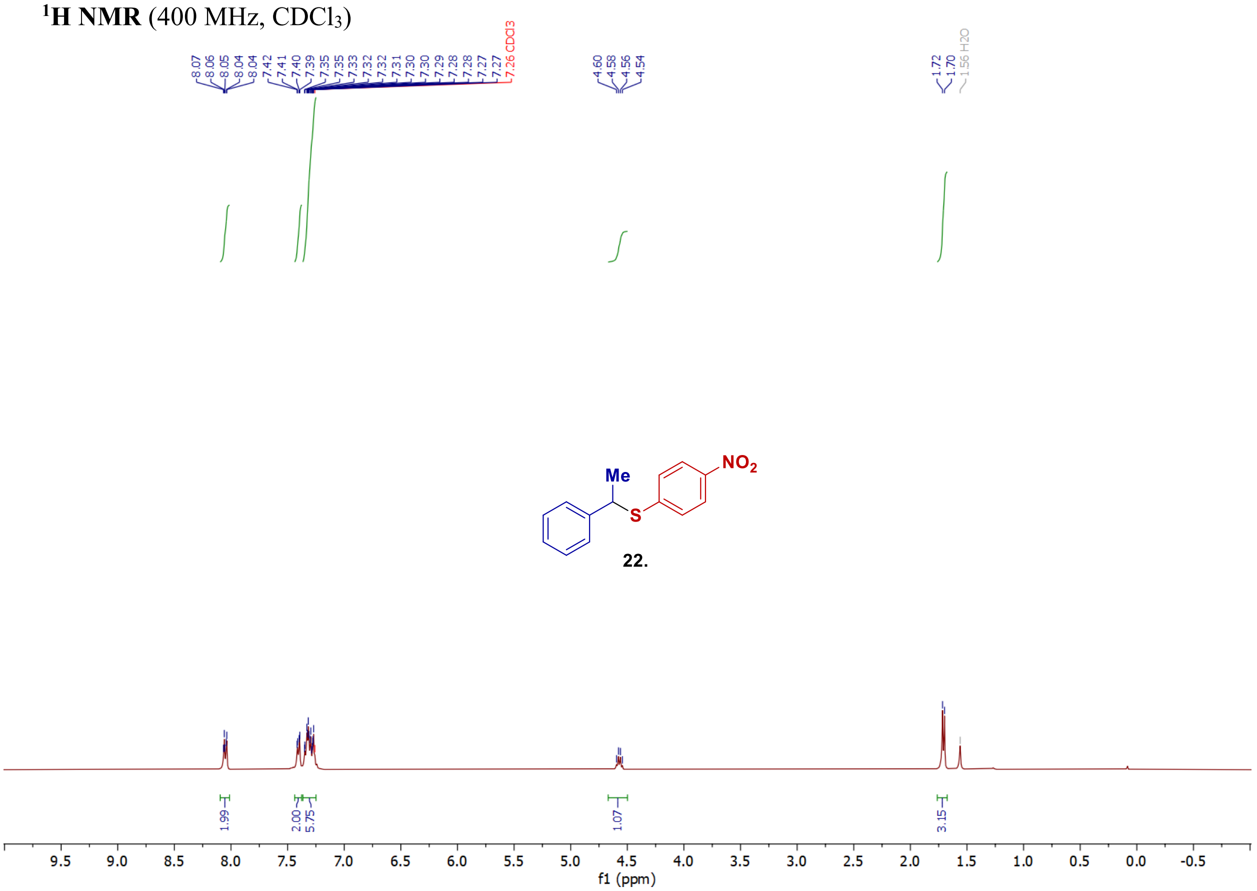

^{13}C NMR (101 MHz, $CDCl_3$)

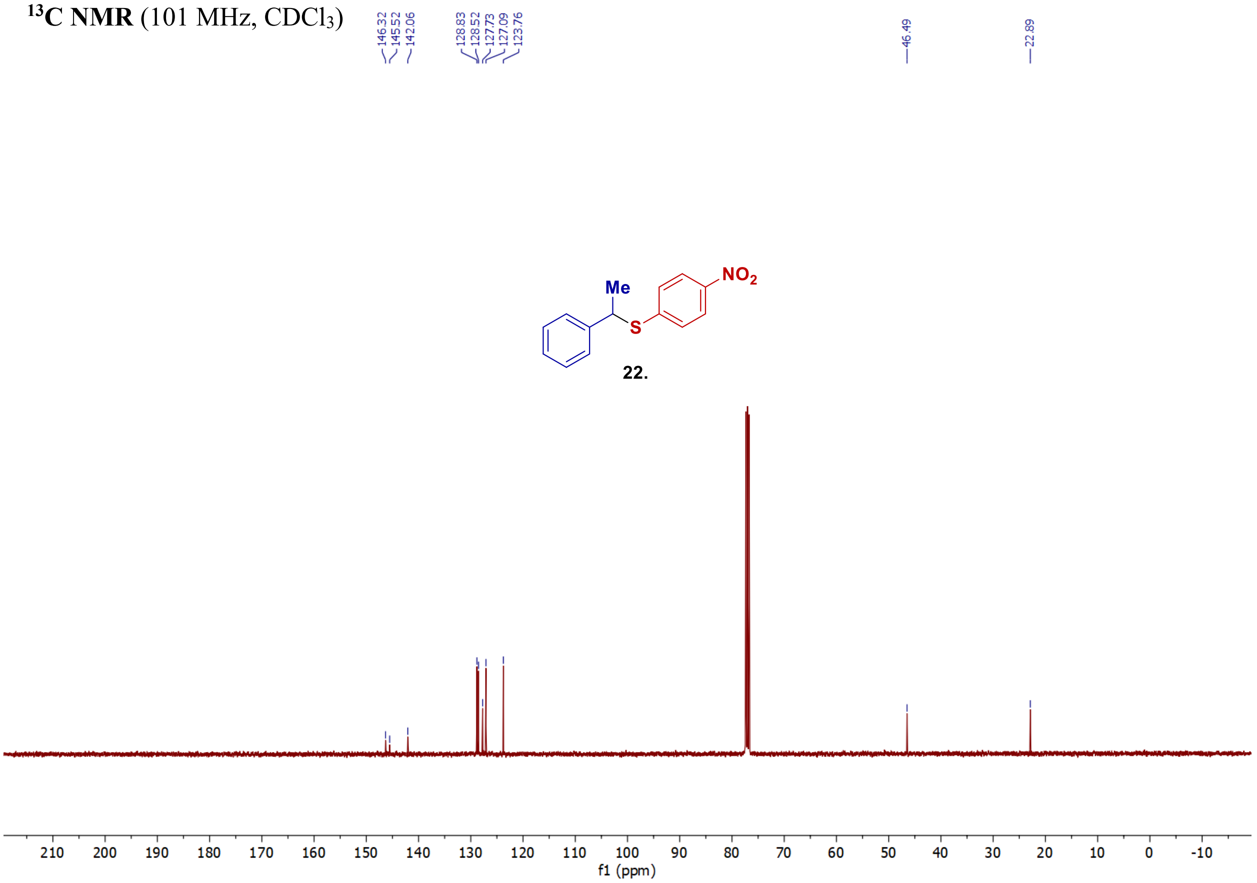

^{1}H NMR (400 MHz, $CDCl_3$)

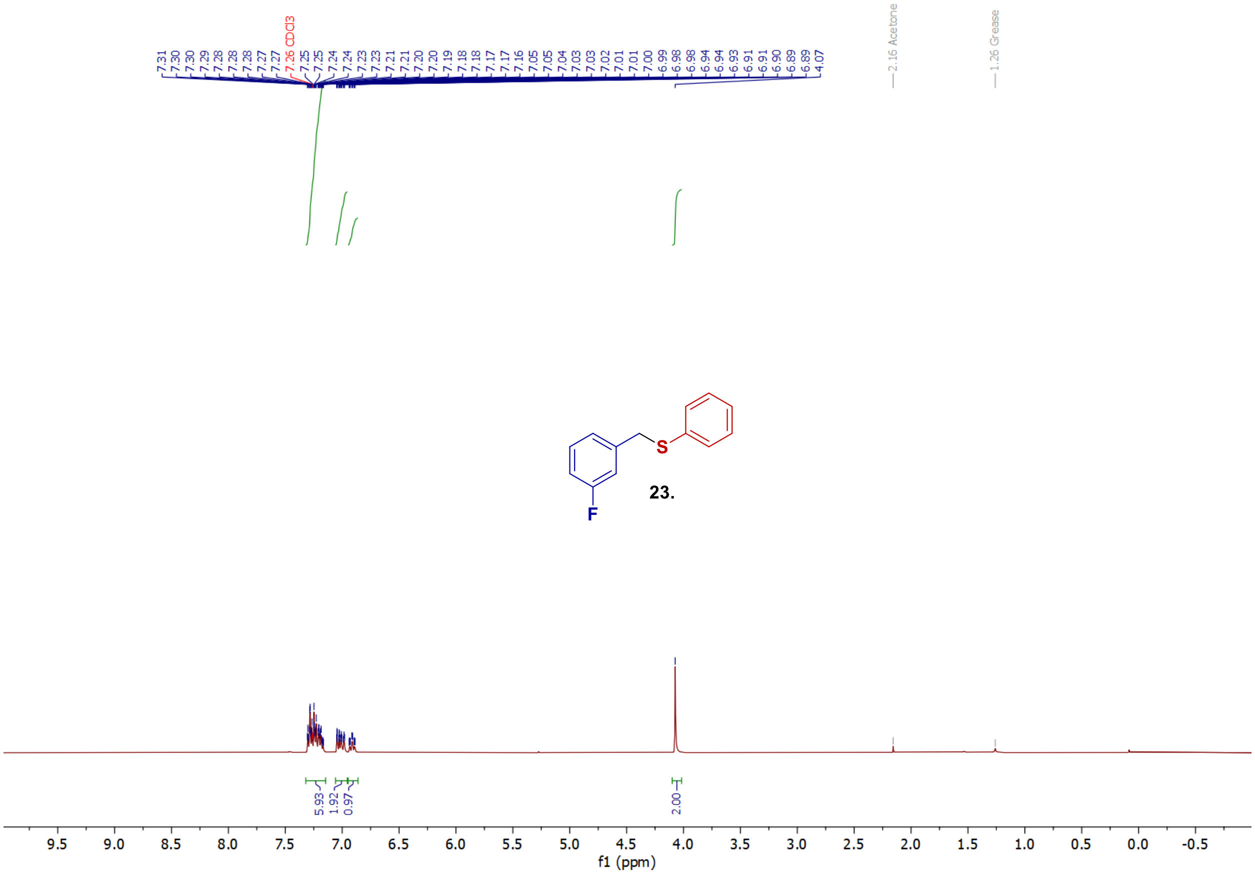

^{13}C NMR (101 MHz, $CDCl_3$)

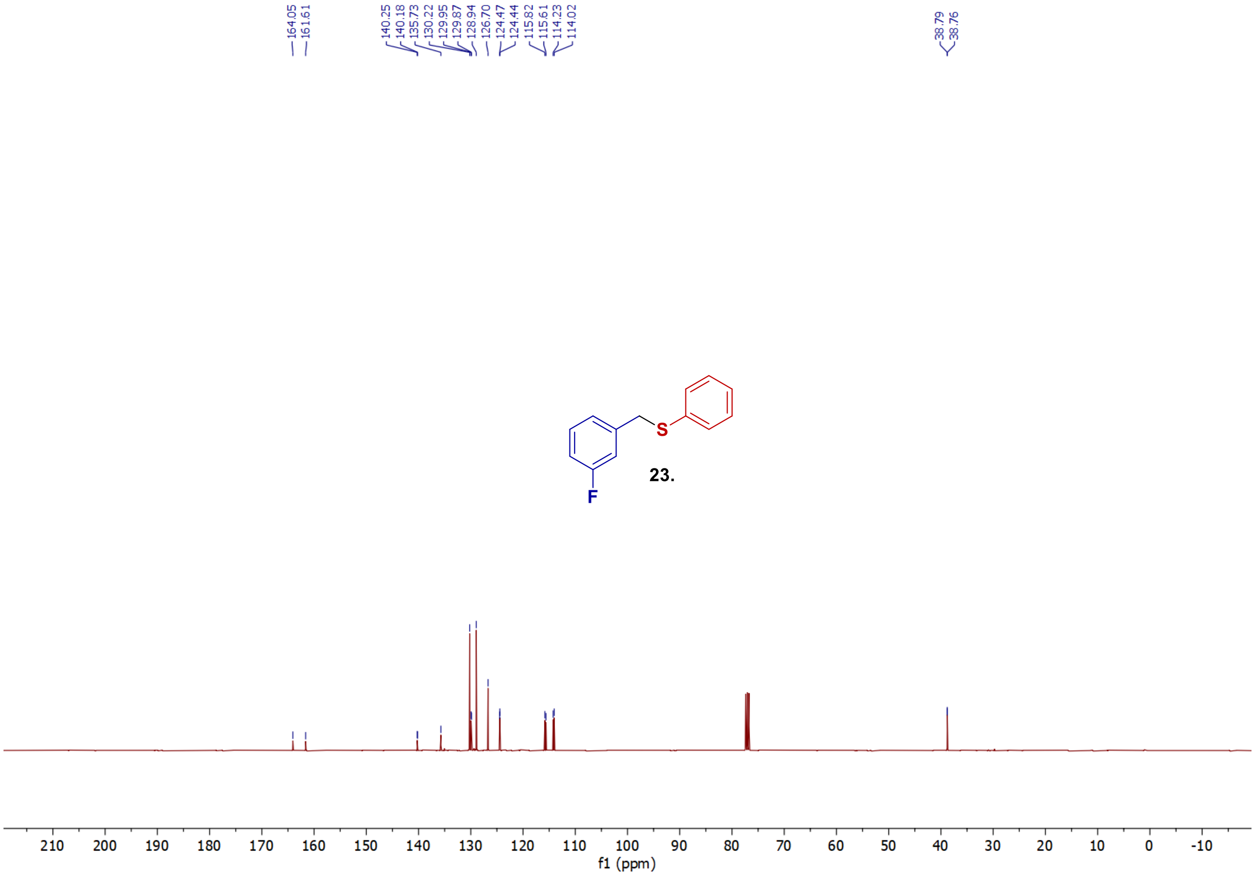

^{19}F NMR (377 MHz, $CDCl_3$)

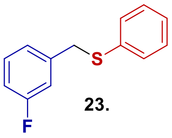

23.

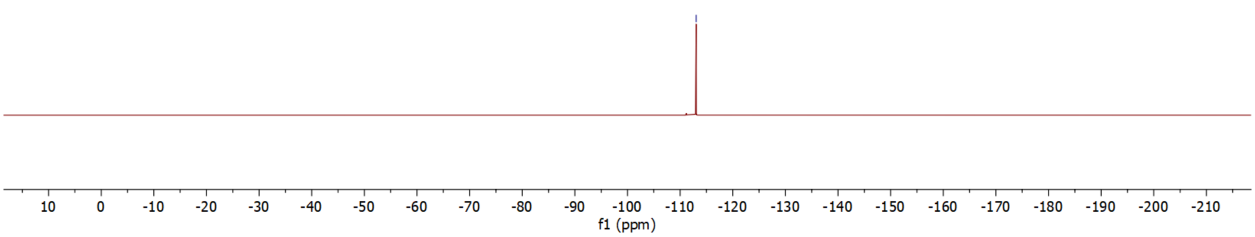

^{1}H NMR (400 MHz, $CDCl_3$)

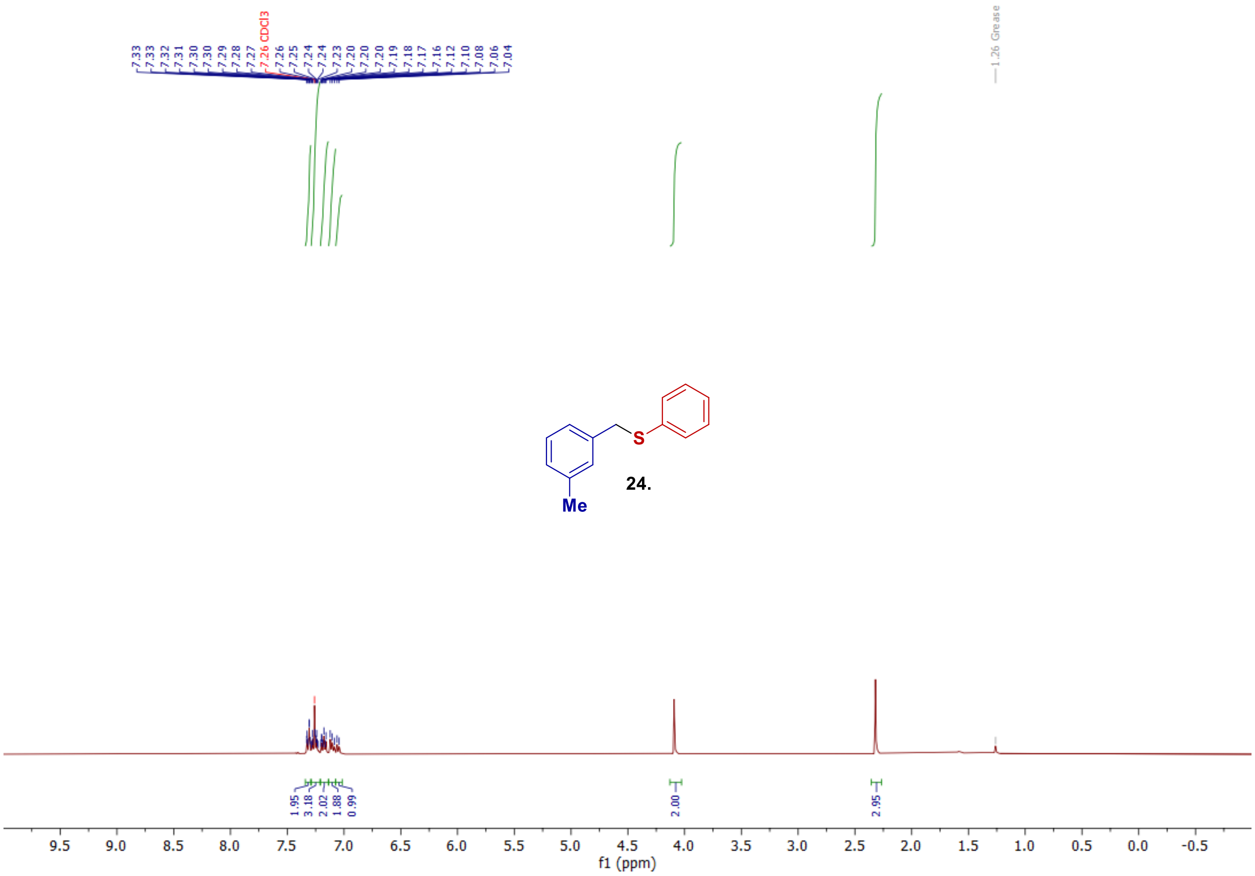

^{13}C NMR (101 MHz, $CDCl_3$)

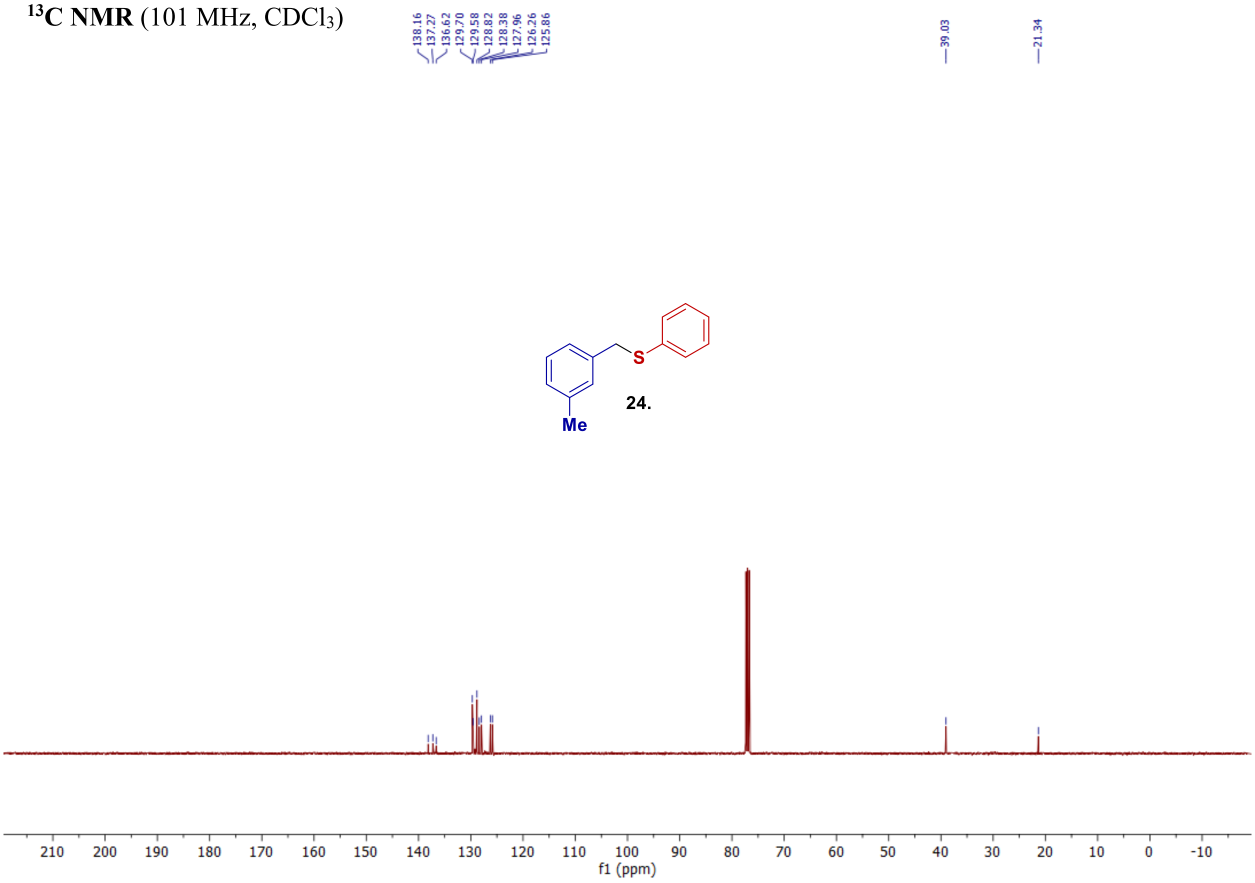

^{1}H NMR (400 MHz, $CDCl_3$)

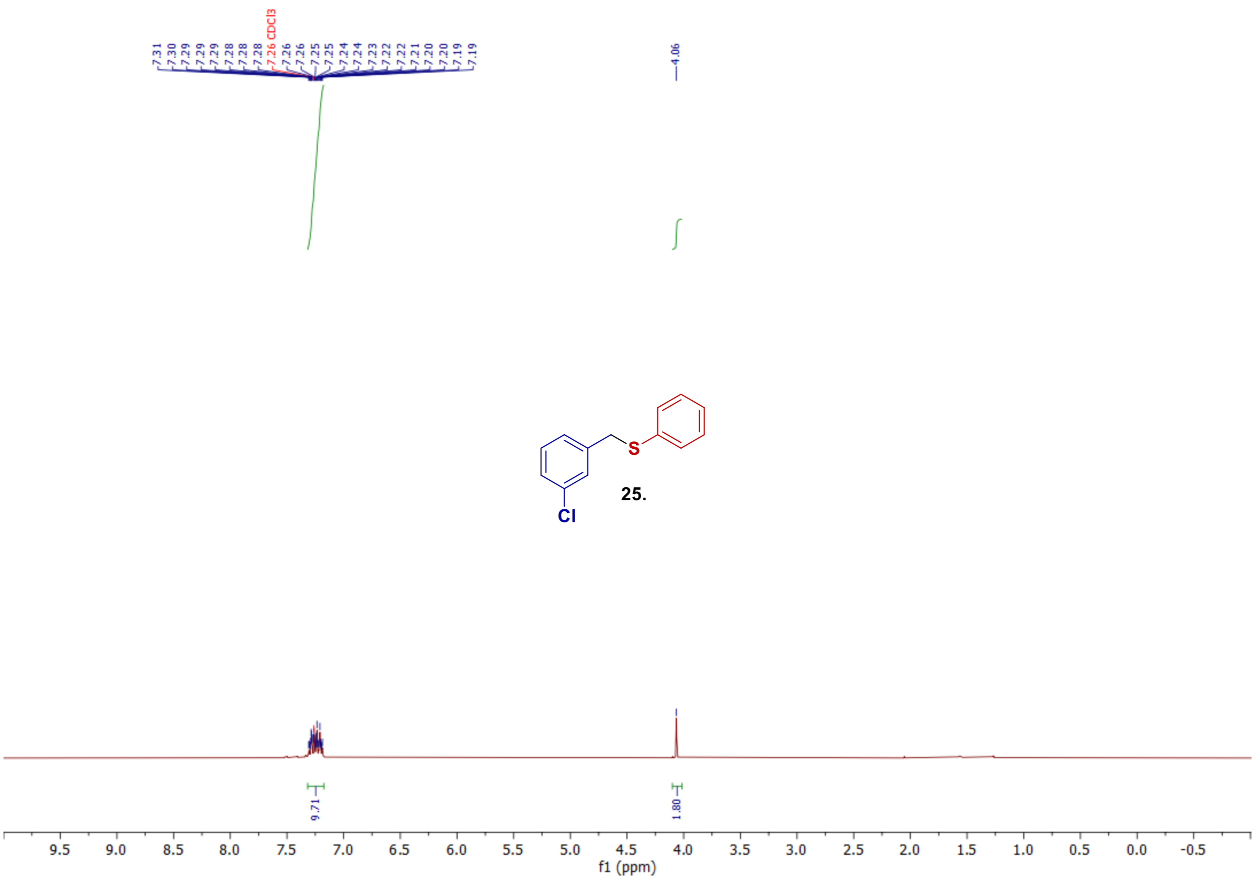

^{13}C NMR (101 MHz, $CDCl_3$)

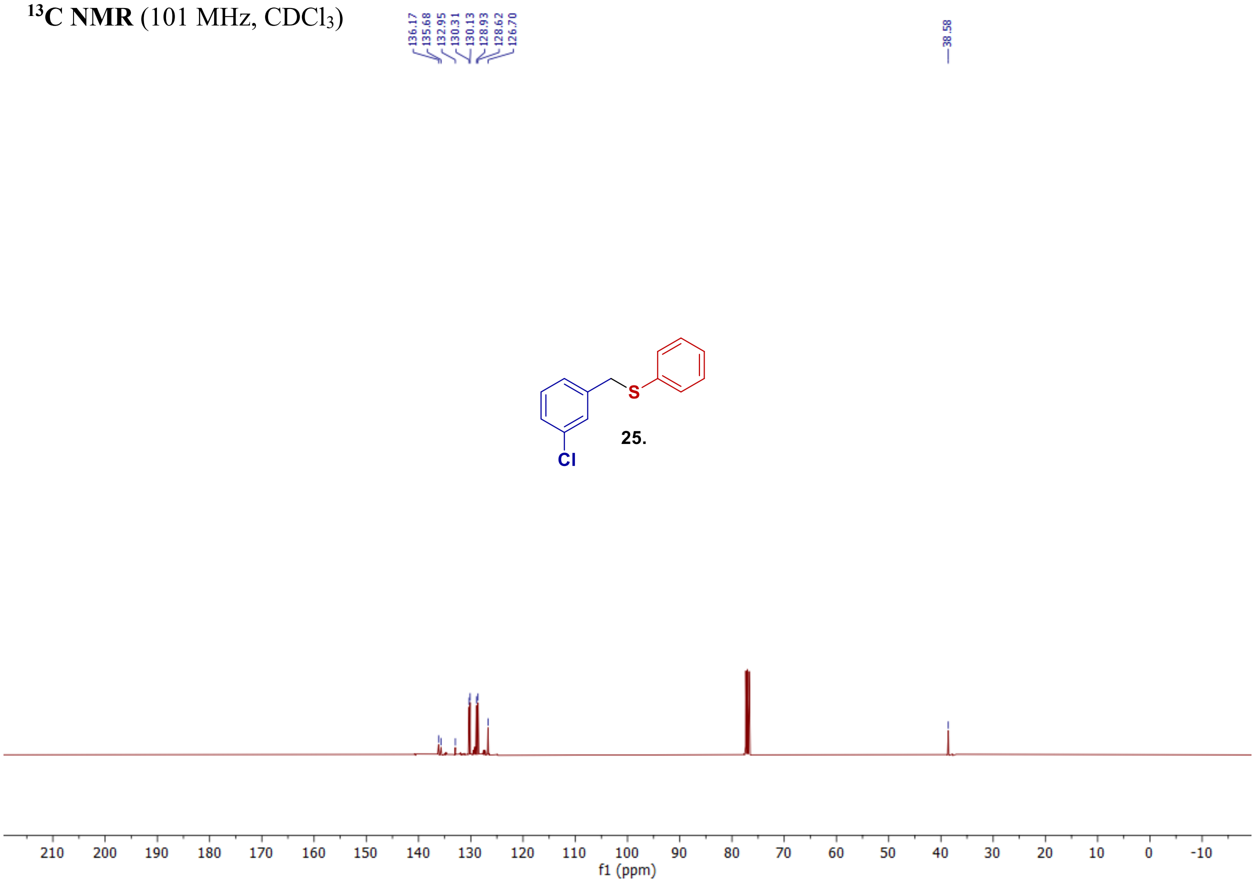

^{1}H NMR (400 MHz, $CDCl_3$)

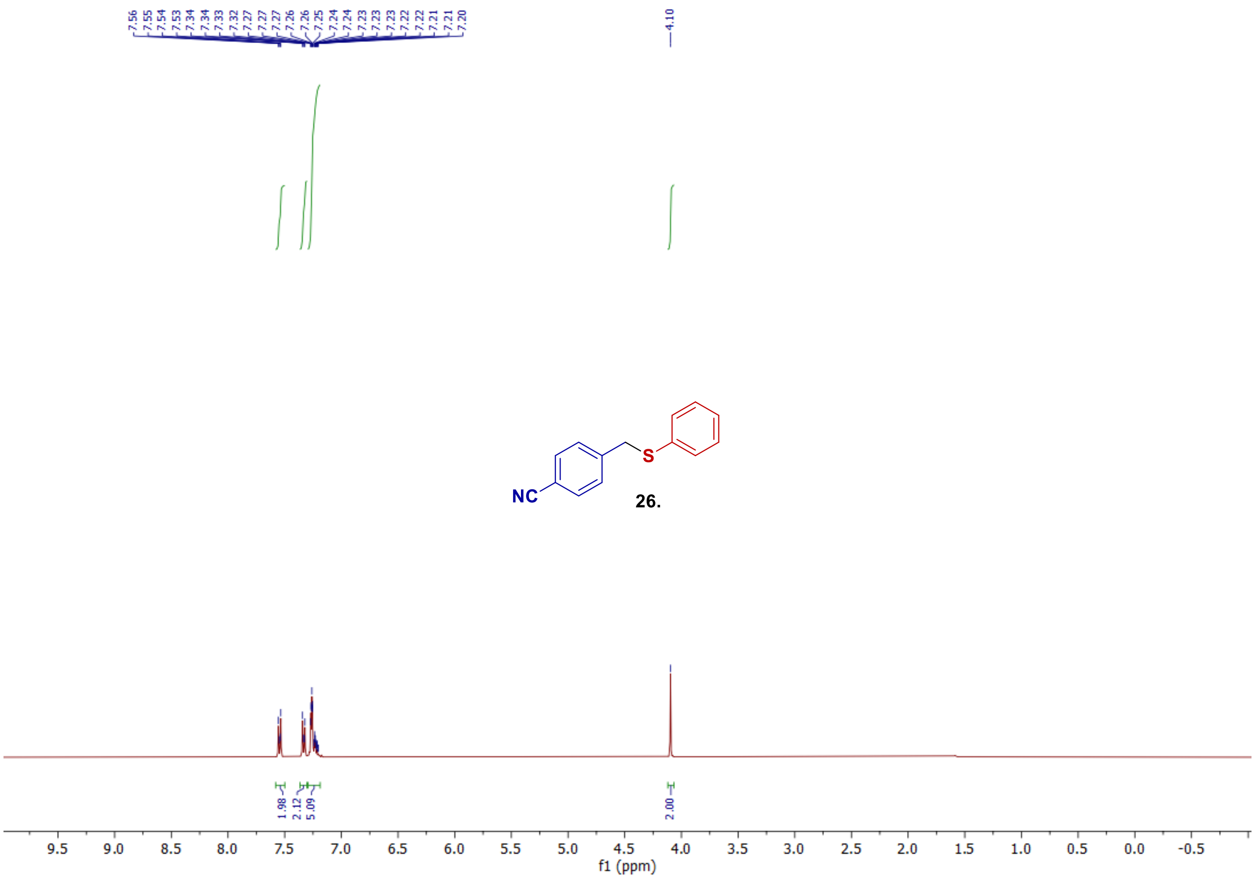

^{13}C NMR (101 MHz, $CDCl_3$)

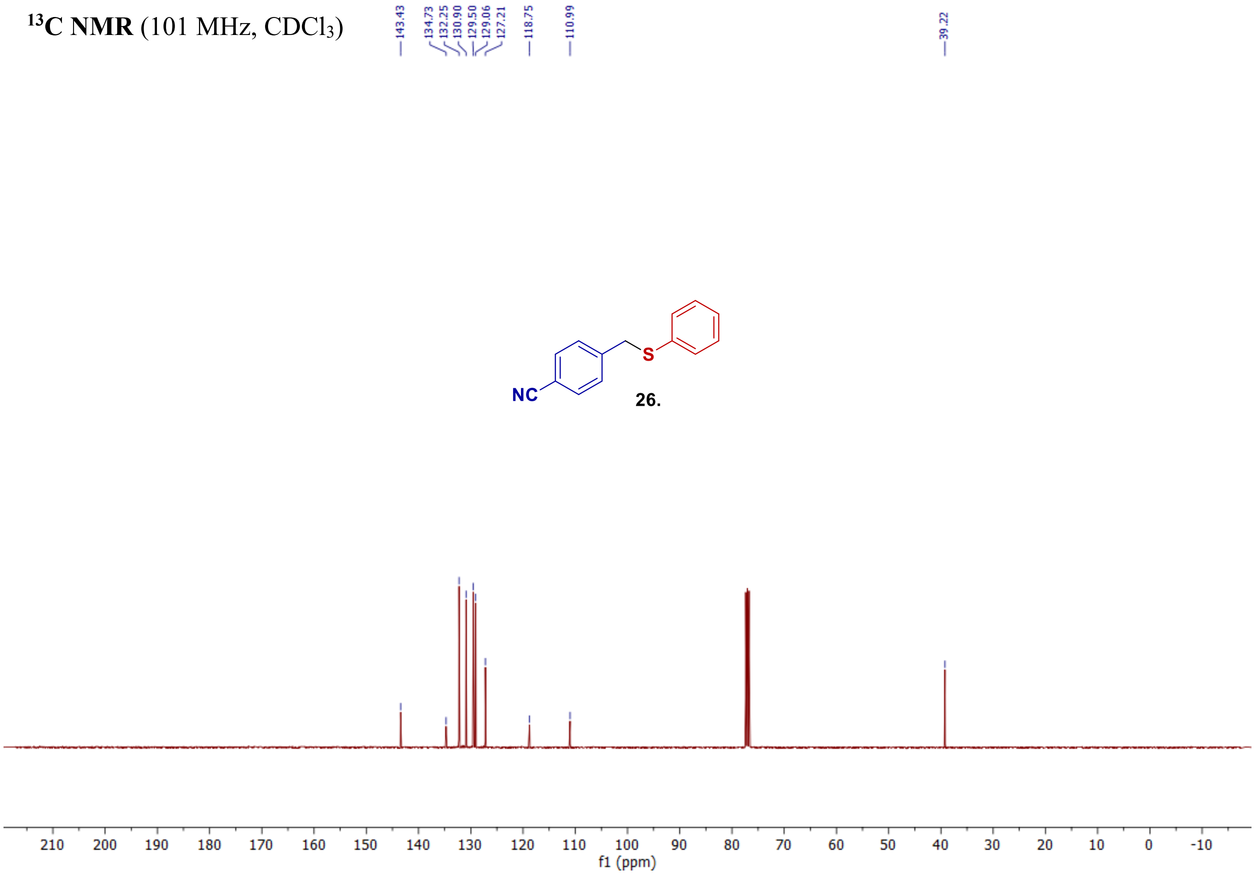

^{1}**H NMR** (400 MHz, $CDCl_3$)

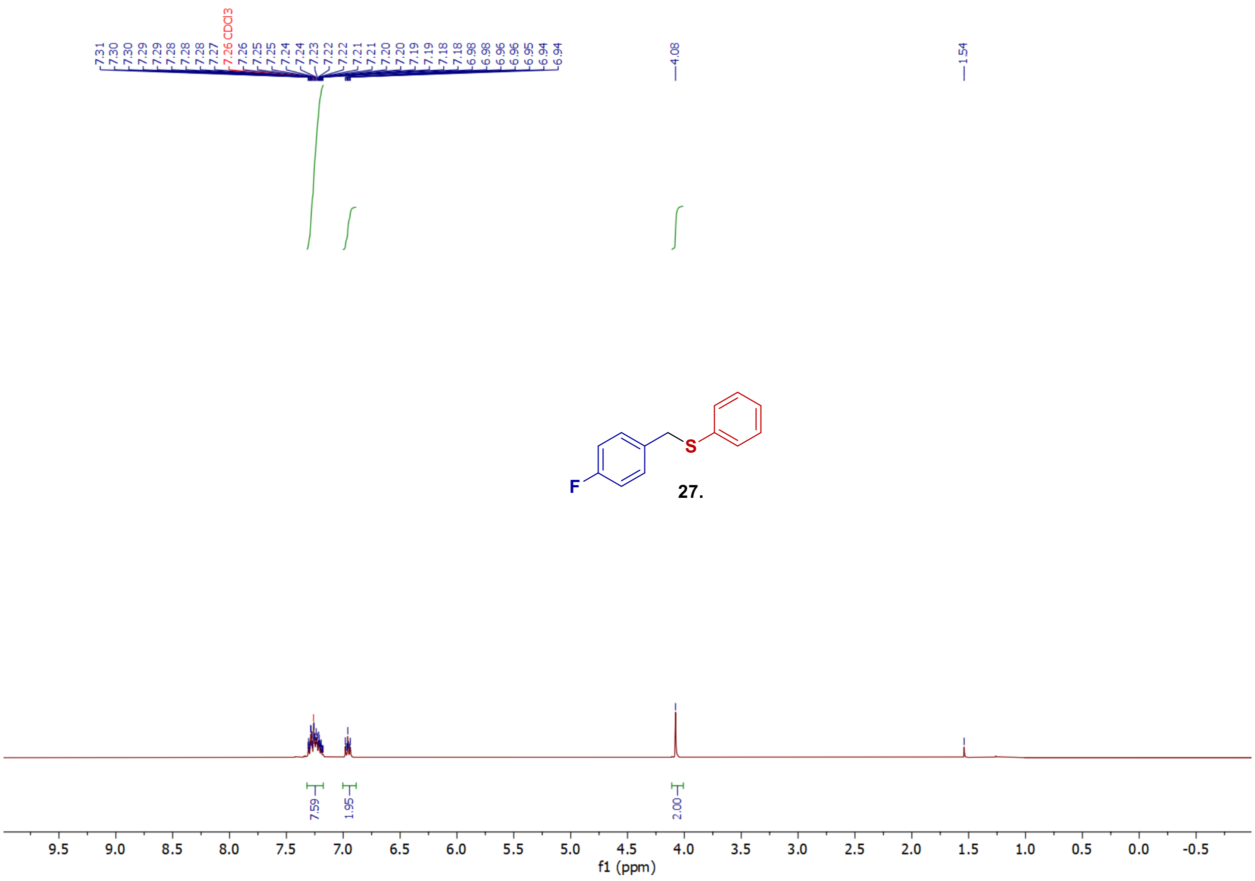

^{13}**C NMR** (101 MHz, $CDCl_3$)

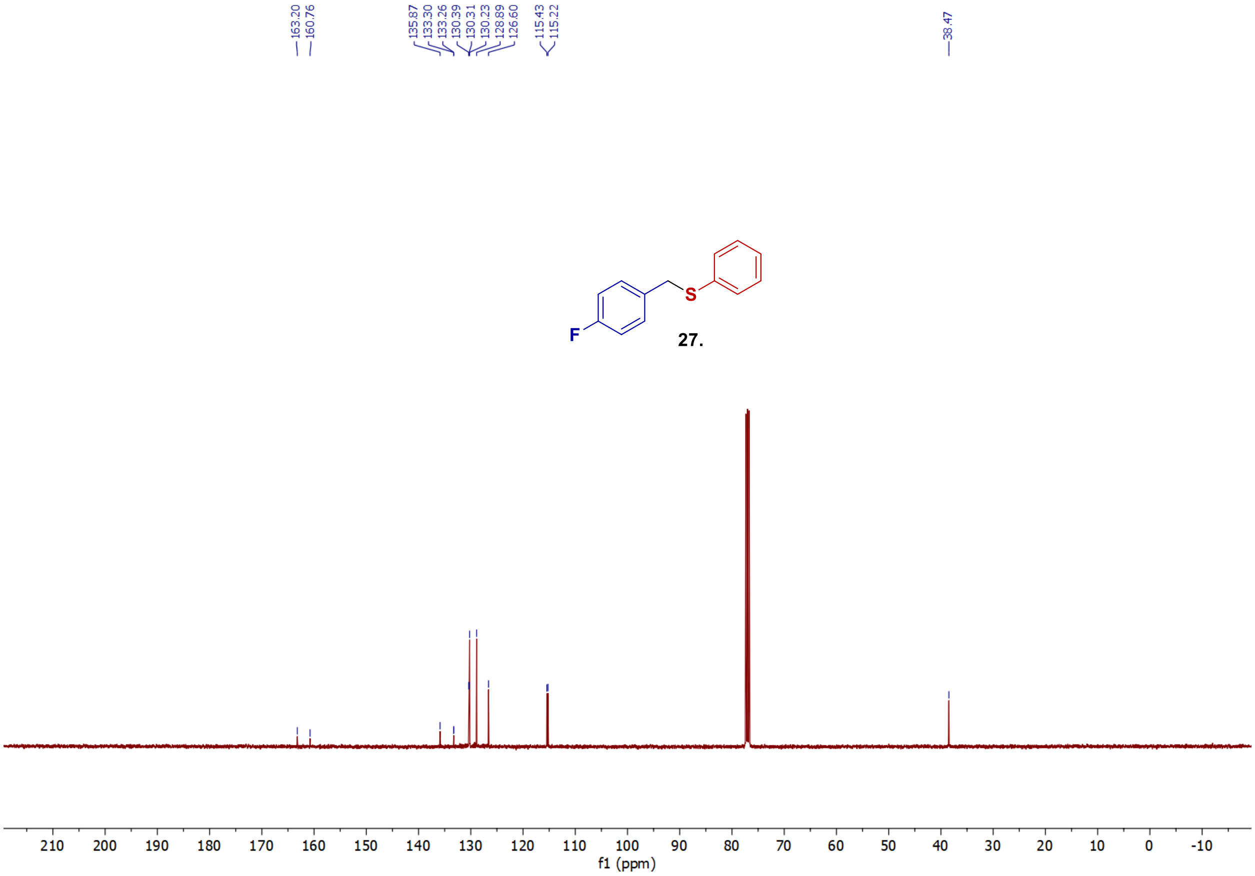

^{19}F NMR (377 MHz, $CDCl_3$)

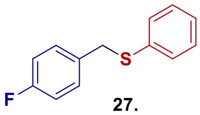

27.

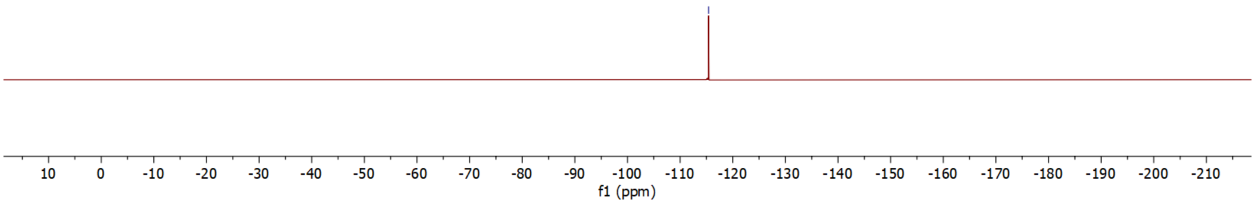

^{1}H NMR (400 MHz, $CDCl_3$)

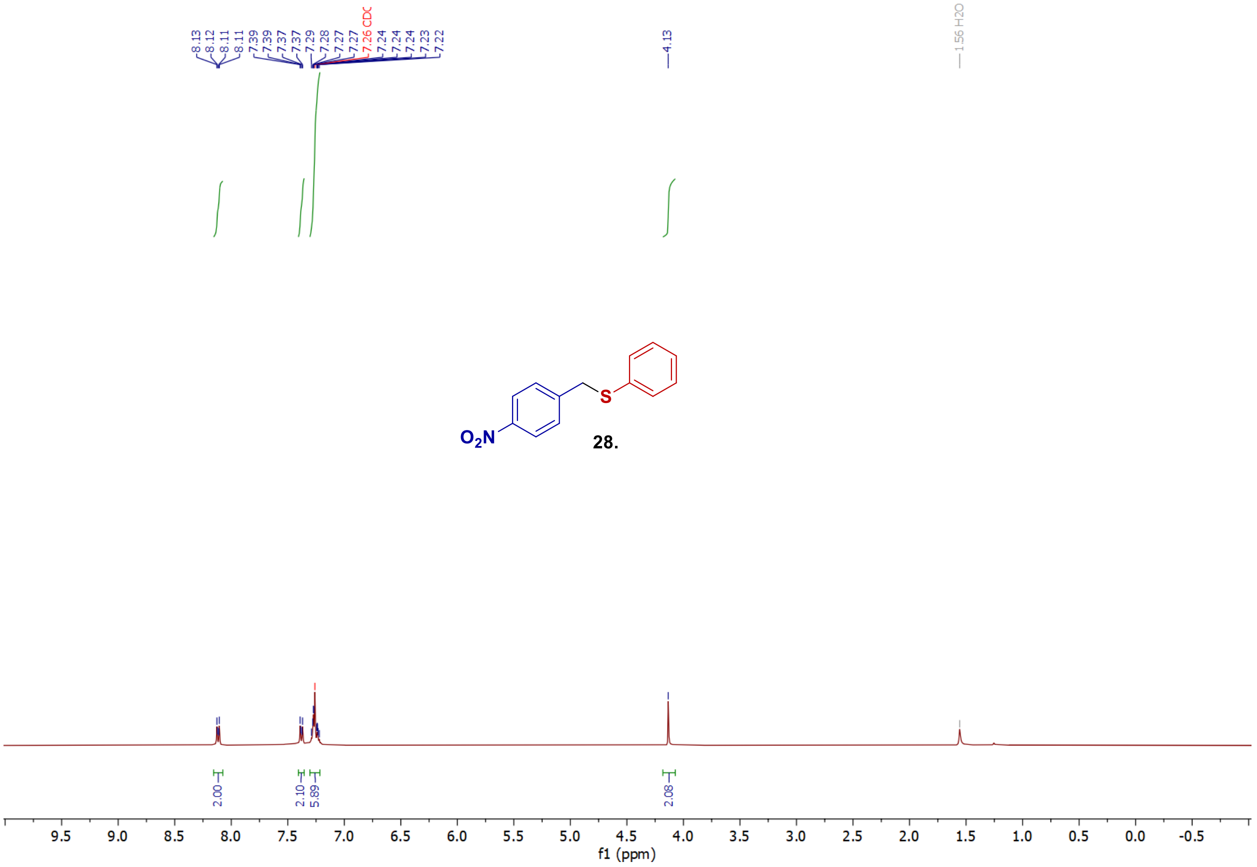

28.

^{13}C NMR (101 MHz, $CDCl_3$)

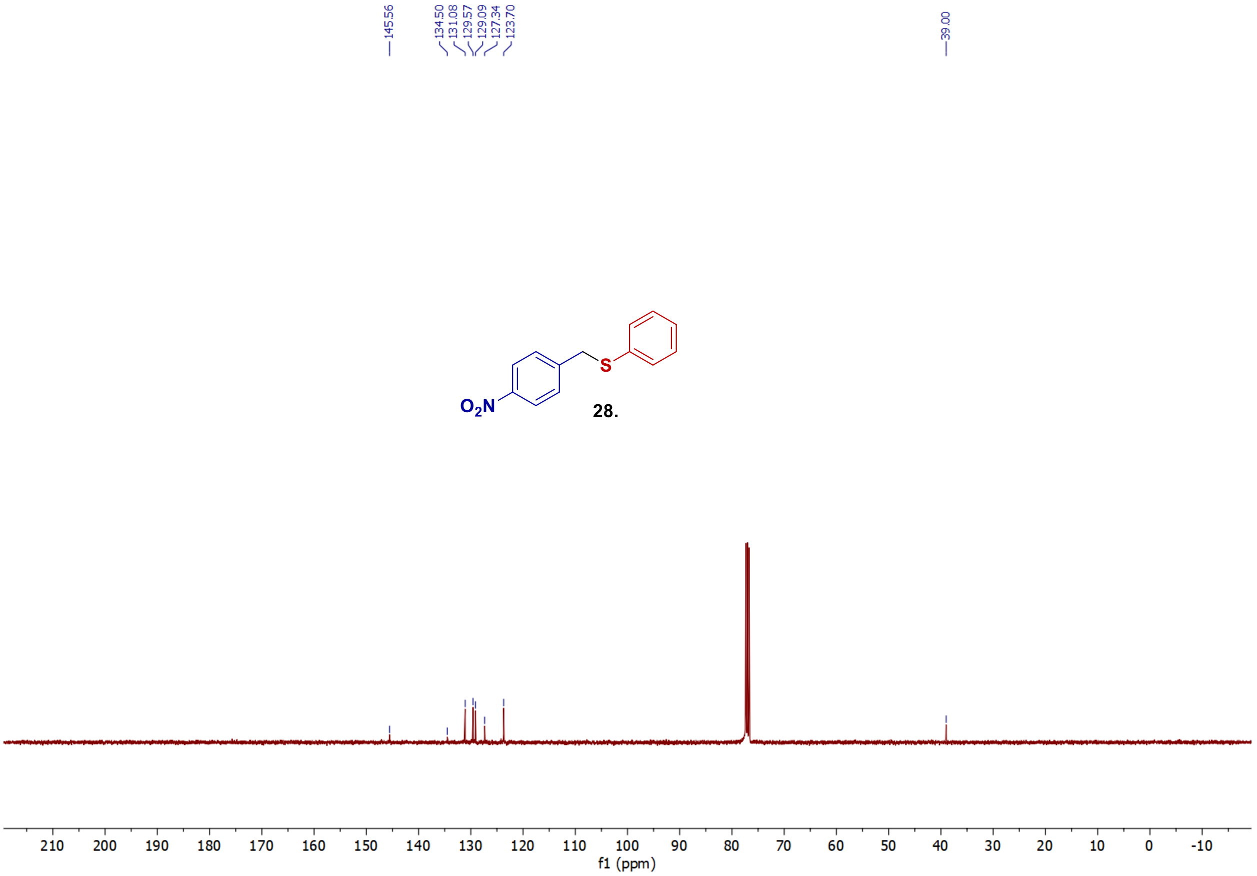

28.

^{1}H NMR (400 MHz, $CDCl_3$)

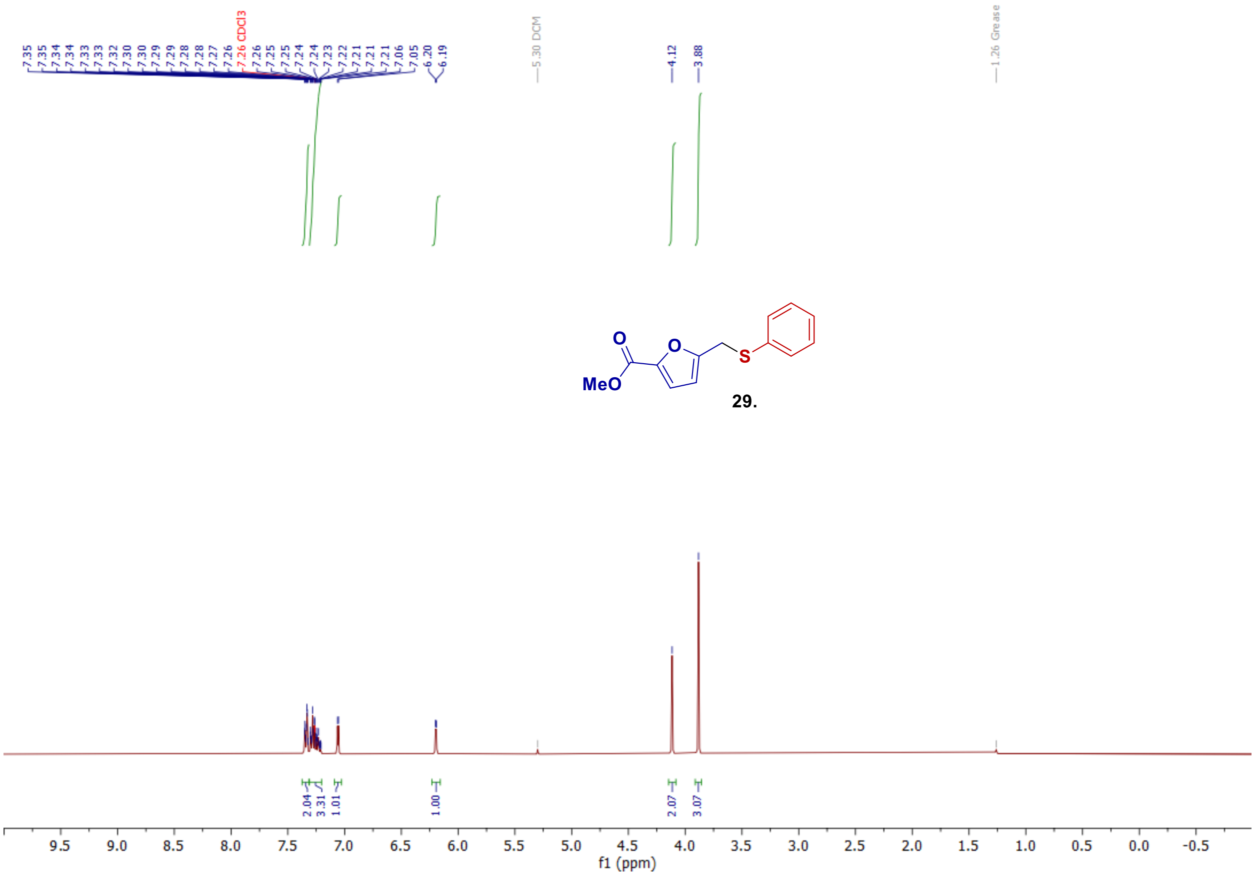

^{13}C NMR (101 MHz, $CDCl_3$)

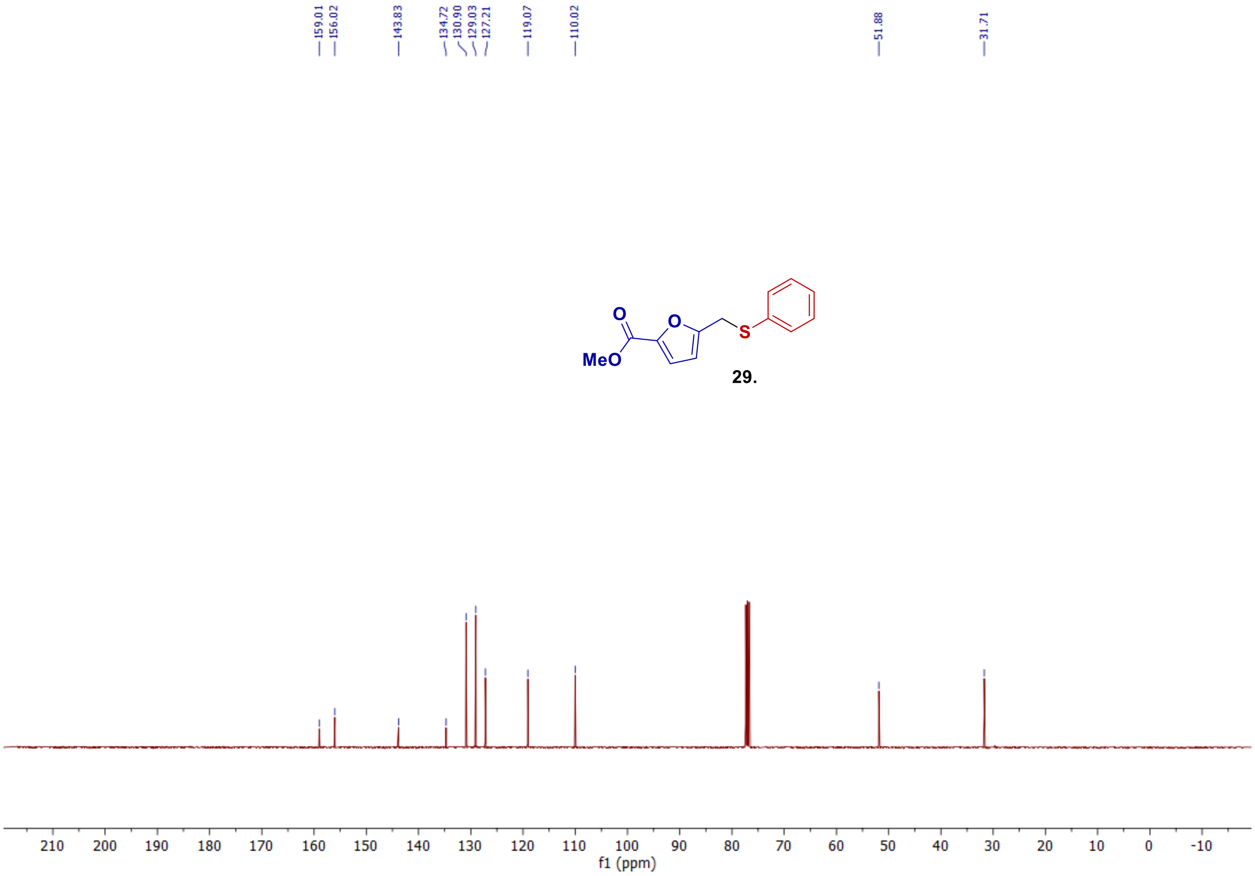

^{1}H NMR (400 MHz, $CDCl_3$)

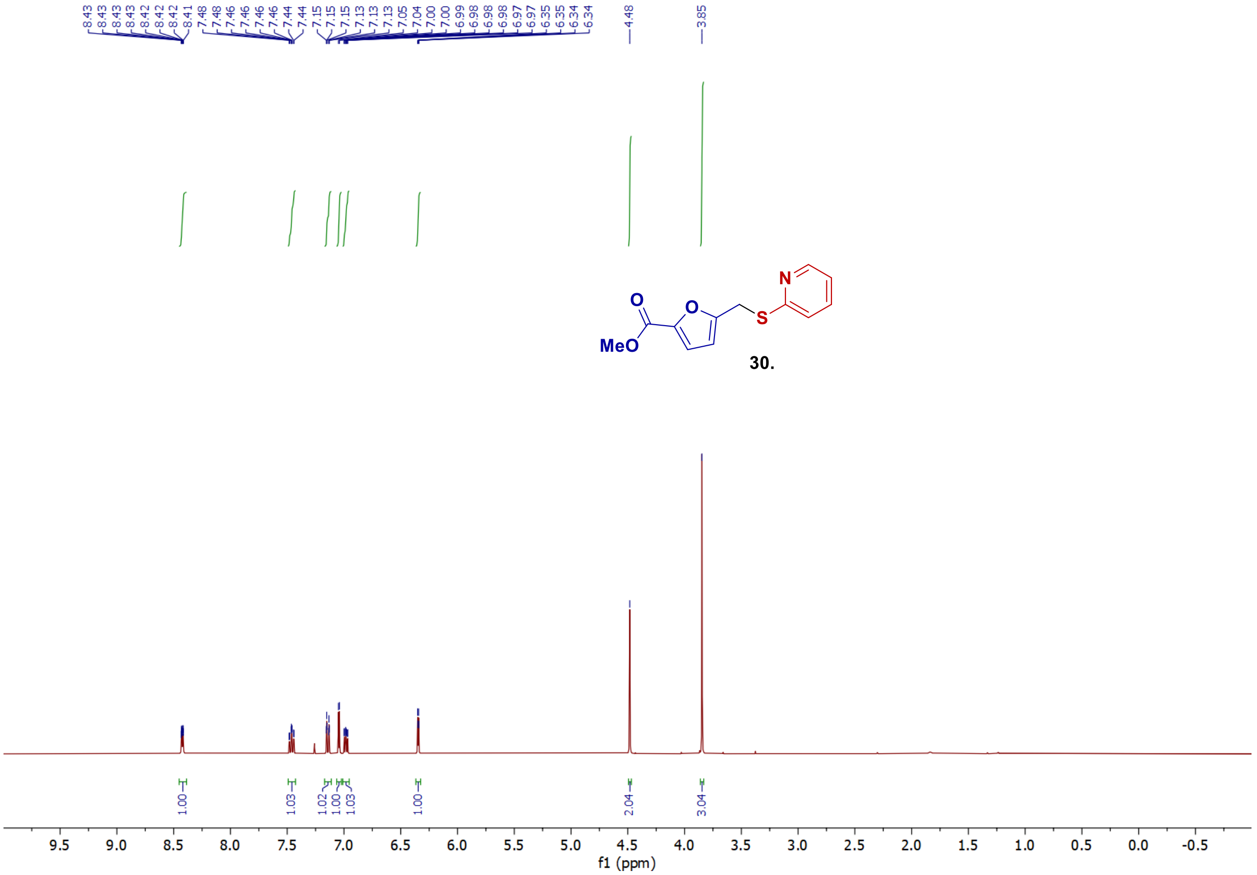

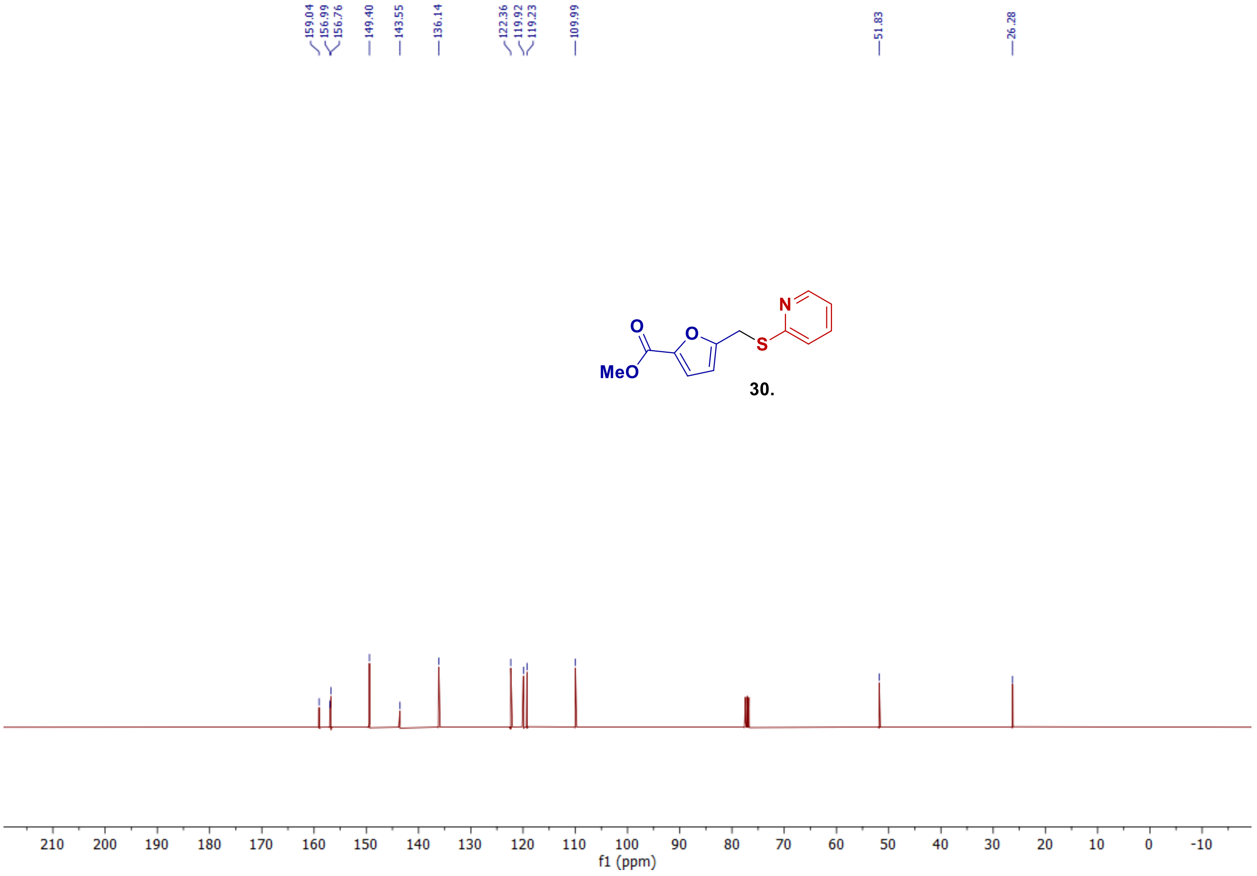

^{1}H NMR (400 MHz, $CDCl_3$)

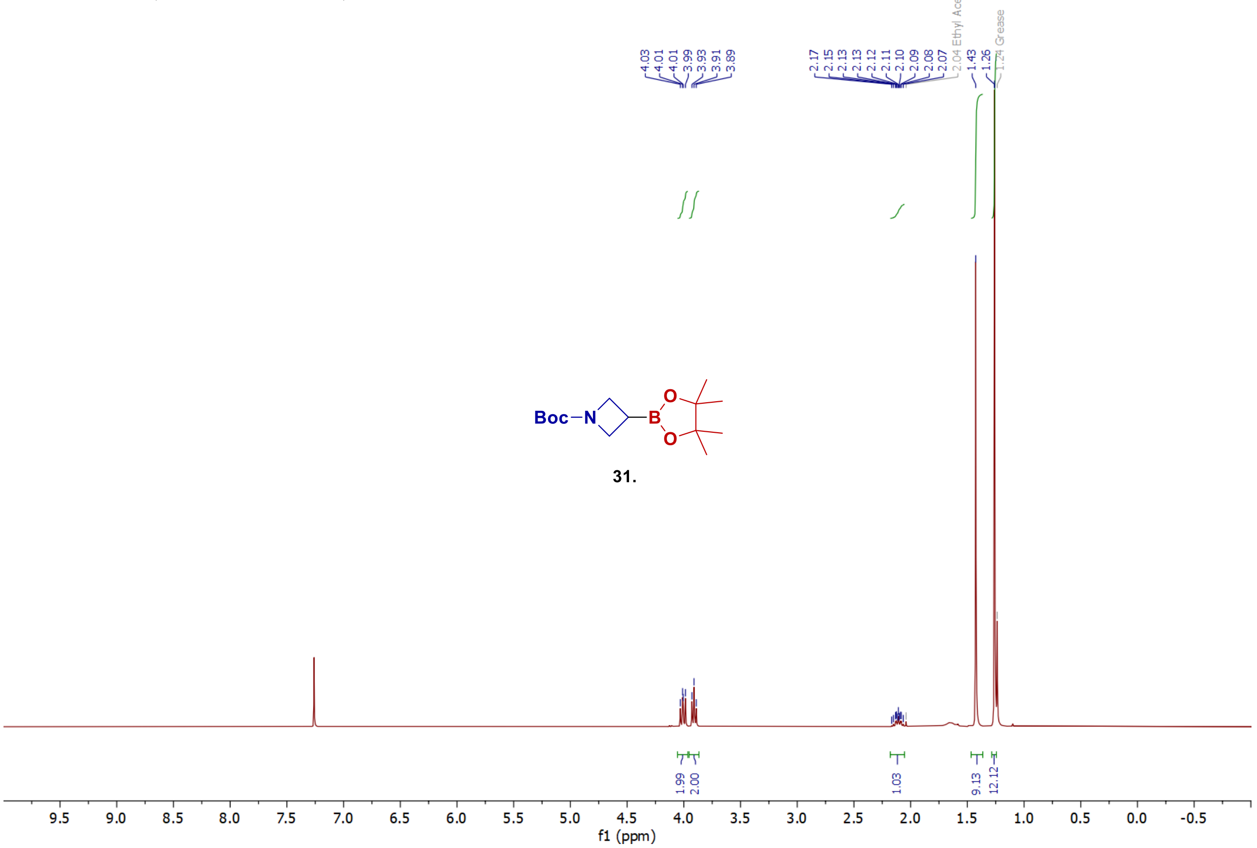

^{13}C NMR (101 MHz, $CDCl_3$)

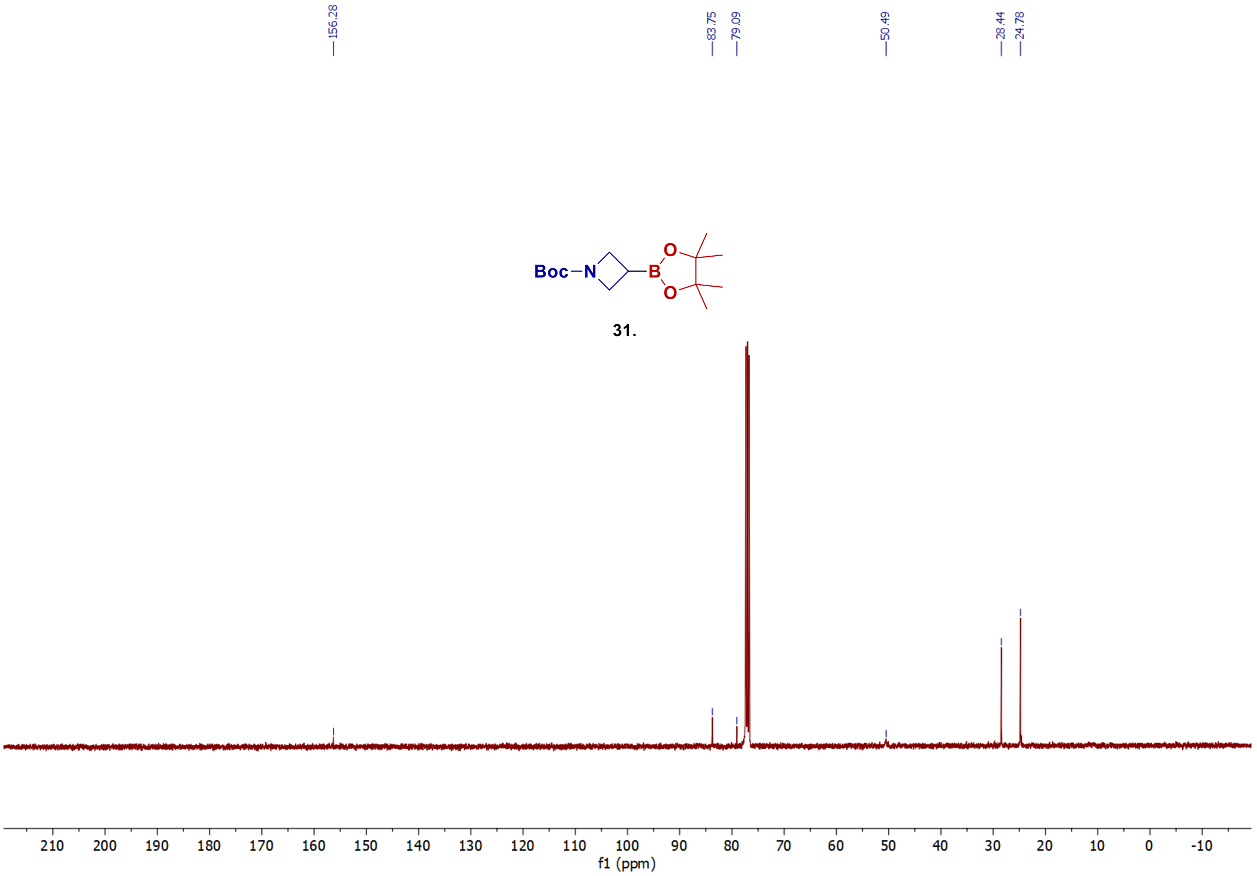

^{1}H NMR (400 MHz, $CDCl_3$)

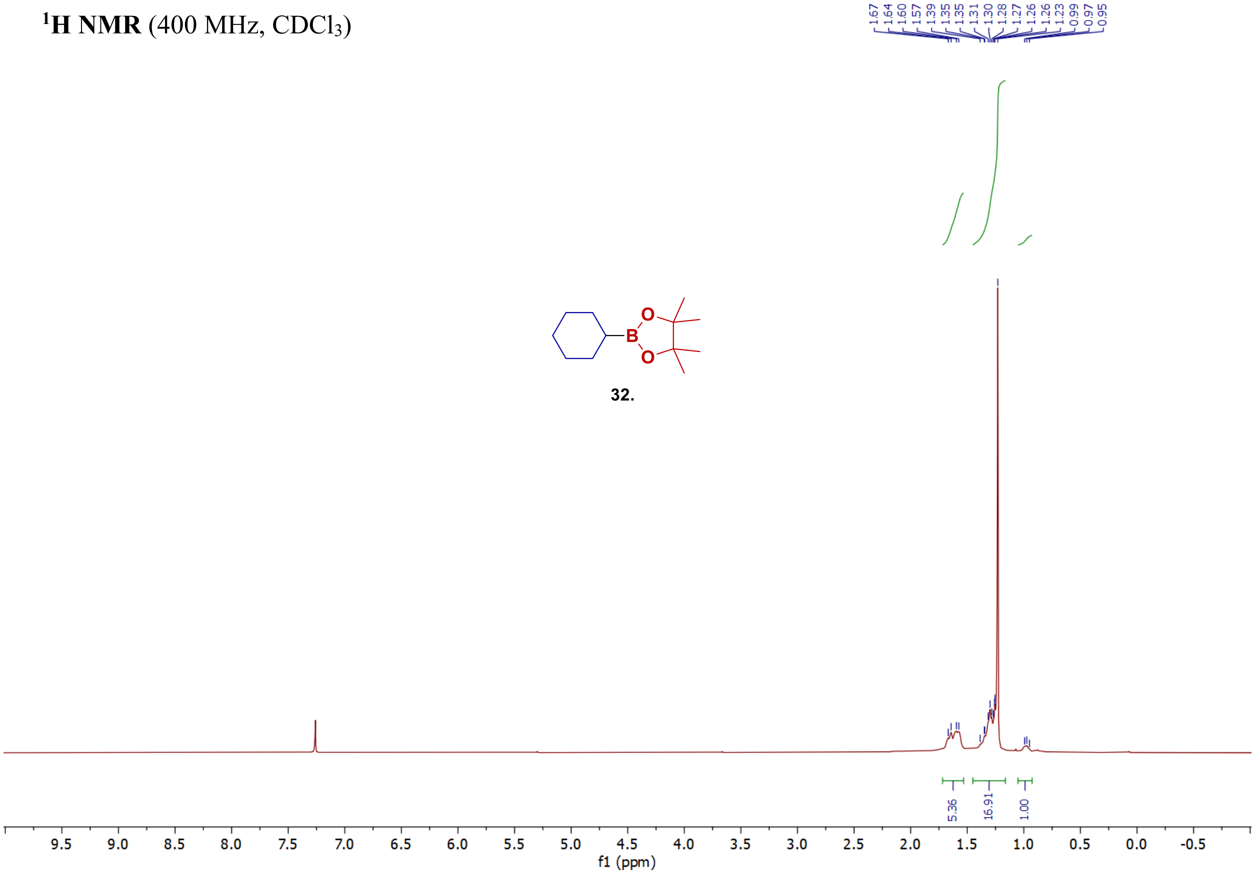

32.

^{13}C NMR (101 MHz, $CDCl_3$)

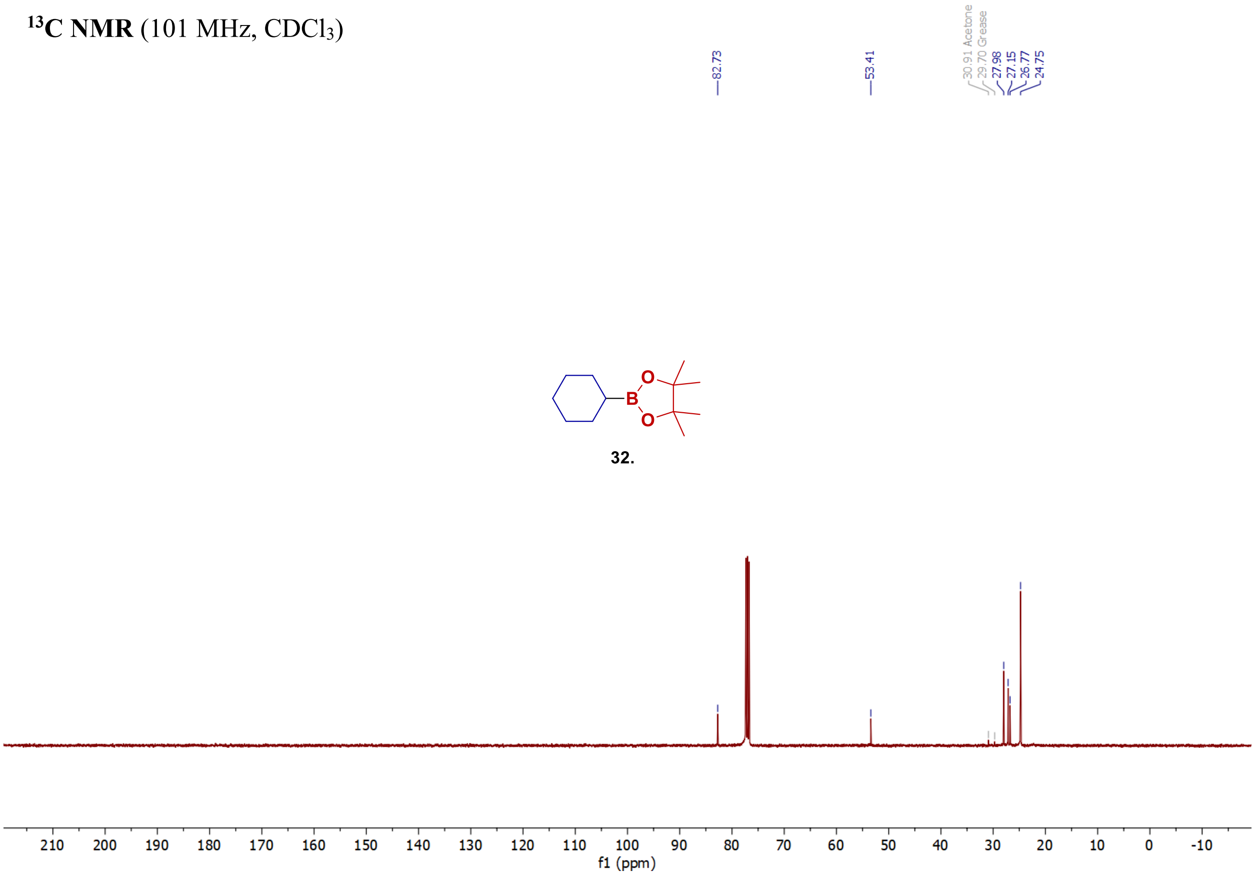

32.

^{1}**H NMR** (400 MHz, $CDCl_3$)

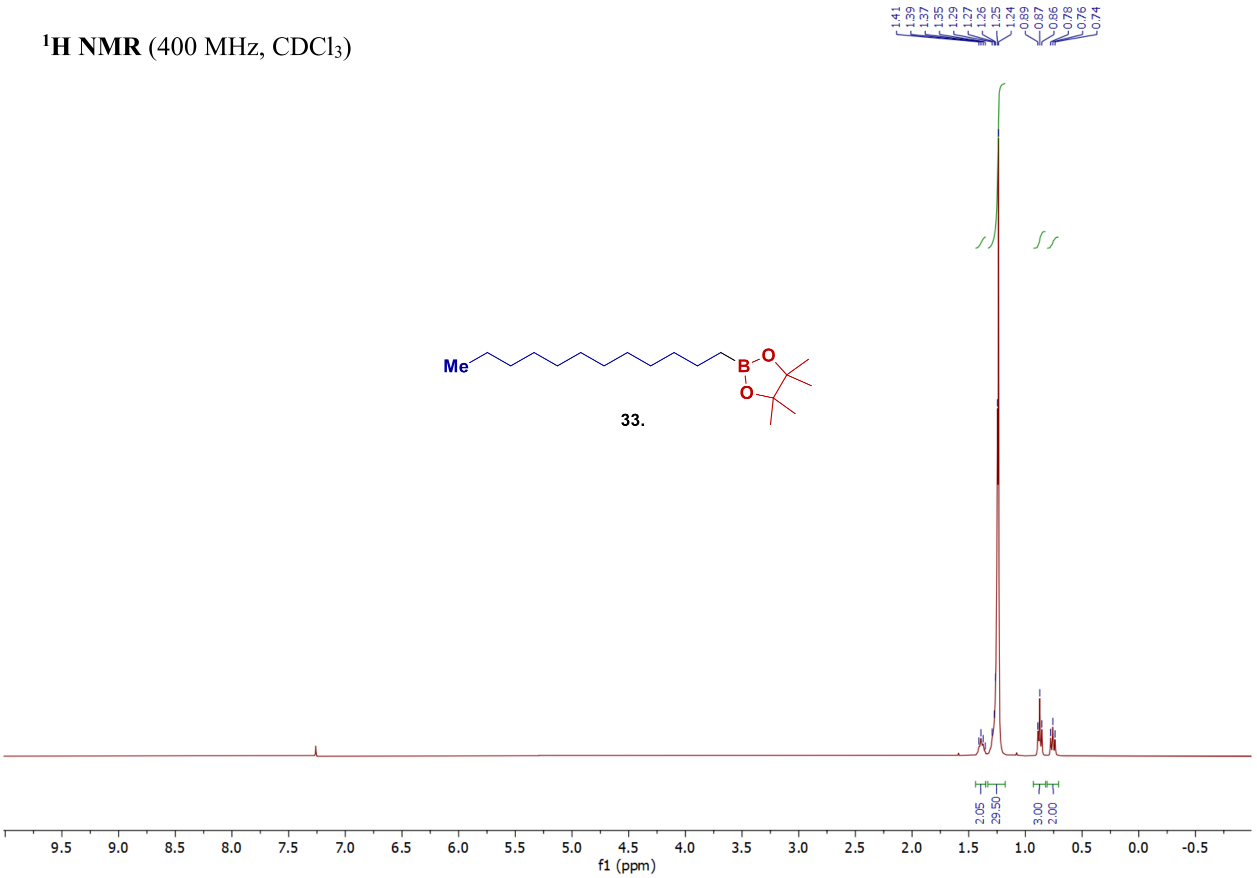

^{13}**C NMR** (101 MHz, $CDCl_3$)

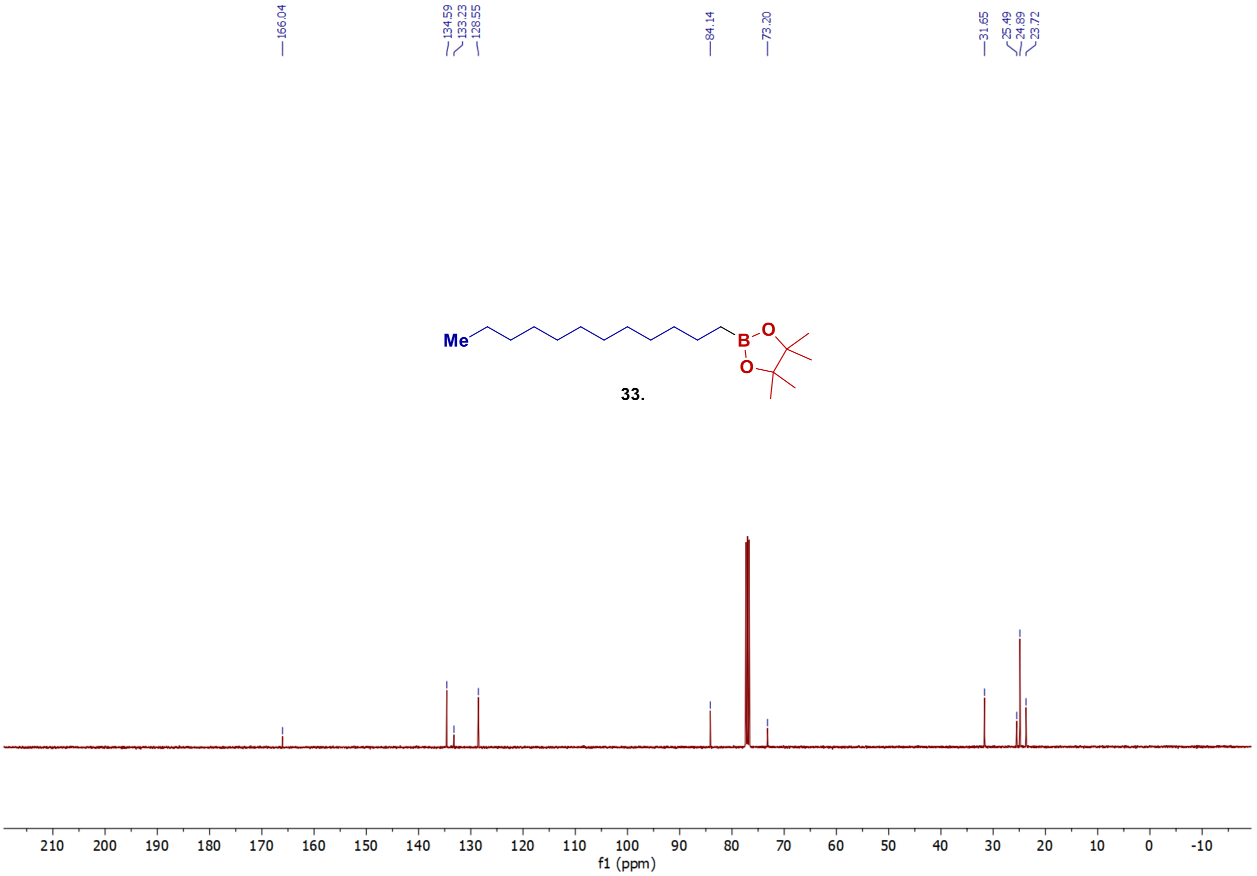

^{1}H NMR (400 MHz, $CDCl_3$)

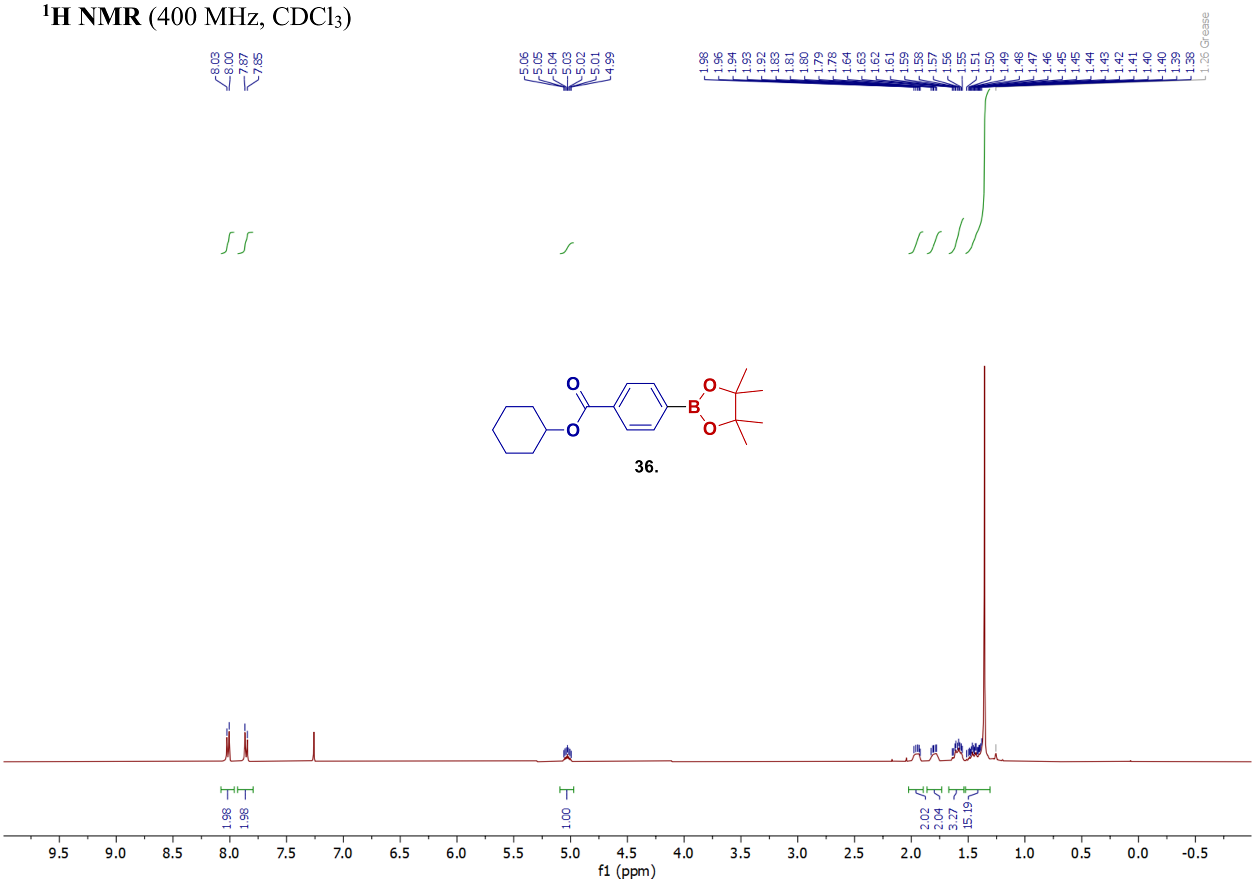

^{13}C NMR (101 MHz, $CDCl_3$)

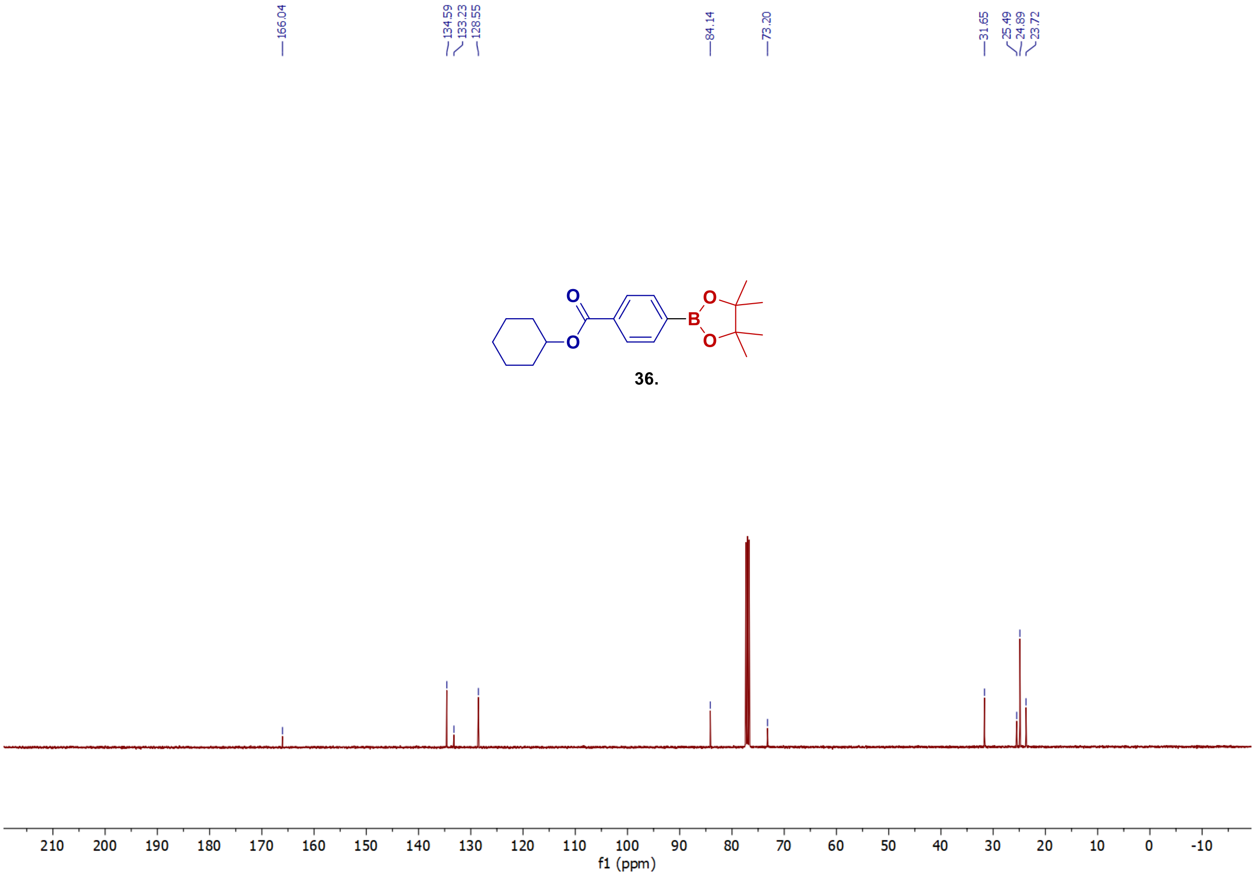

13. References.

1. (a) P. J. Garegg, B. Samuelsson, *J. Chem. Soc., Perkin Trans. 1* **1980**, 2866-2869. (b) H. Finkelstein, *Ber. Dtsch. Chem. Ges.* **1910**, *43*, 1528-1532.
2. I. Quirós, M. Martín, M. Gomez-Mendoza, M. J. Cabrera-Afonso, M. Liras, I. Fernández, L. Nóvoa, M. Tortosa, *Angew. Chem., Int. Ed.* **2024**, *136*, e202317683.
3. A. Dey, S. Chakraborty, A. Singh, F. A. Rahimi, S. Biswas, T. Mandal, T. K. Maji, *Angew. Chem., Int. Ed.* **2024**, *63*, e202403093.
4. T. C. Castral, A. P. Matos, J. L. Monteiro, F. M. Araujo, T. M. Bondancia, L. G. Batista-Pereira, J. B. Fernandes, P. C. Vieira, M. F. G. da Silva, A. G. Corrêa, *J. Agric. Food Chem* **2011**, *59*, 4822-4827.
5. S. S. Shah, M. Shee, Y. Venkatesh, A. K. Singh, S. Samanta, N. P. Singh, *Chem. Eur. J.* **2020**, *26*, 3703-3708.
6. R. Ramkumar, S. Chandrasekaran, *Synthesis* **2019**, *51*, 921–932
7. M. Mart, I. Karakaya, J. Jurczak, *ChemistrySelect* **2022**, *7*, e202202436.
8. Z. Li, K.-F. Wang, X. Zhao, H. Ti, X.-G. Liu, H. Wang, Nat. Commun. **2020**, *11*, 5036.
9. J.-T. Ge, Y.-Y. Li, J. Tian, R.-Z. Liao, H. Dong, *J. Org. Chem.* **2017**, *82*, 7008-7014.
10. X. Liu, J. Qin, W. Dai, Z. Zhu, P. Zhou, Y. Wang, J. Nie, Y. Yang, Z. Zhang, *ACS Catal.* **2022**, *12*, 13300-13311.
11. Y. Hu, B. Li, *Tetrahedron* **2017**, *73*, 7301-7307.
12. Y. Nishii, T. Hirai, S. Fernandez, P. Knochel, K. Mashima, *Eur. J. Org. Chem.* **2017**, *2017*, 5010-5014.
13. S. Gaware, R. Chatterjee, A. R. Kapdi, R. Dandela, *Org. Biomol. Chem.* **2023**, *21*, 5176-5180.
14. J. H. Kuhlmann, M. Uygur, O. García Mancheño, *Org. Lett.* **2022**, *24*, 1689-1694.
15. J. Semenya, Y. Yang, E. Picazo, *J. Am. Chem. Soc.* **2024**, *146*, 4903-4912.
16. Y. Gui, S.-K. Tian, *Org. Lett.* **2017**, *19*, 1554-1557.
17. C. Gong, J. Huang, L. Cai, Y. Yuan, T. Pu, M. Huang, S.-H. Wu, L. Wang, *J. Org. Chem.* **2024**, *89*, 9450-9461.
18. C.-H. Long, H. Cai, Y. Sun, Z.-N. Cai, H.-B. Qin, *Org. Chem. Front.* **2025**.
19. Y.-H. He, Z. Guan, *Org. Chem. Front.* **2024**, *11*, 6790-6796.

20. I. F. Yu, K. A. D'Angelo, Á. D. Hernandez-Mejías, N. Cheng, J. F. Hartwig, *J. Am. Chem. Soc.* **2024**, *146*, 7124-7129.
21. M. Wollenburg, D. Moock, F. Glorius, *Angew. Chem. Int. Ed.* **2019**, *58*, 6549-6553.
22. S. Weber, D. Zobernig, B. Stöger, L. F. Veiros, K. Kirchner, *Angew. Chem. Int. Ed.* **2021**, *60*, 24488-24492.
23. M. Yang, T. Chen, Z.-F. Xu, M. Yu, C.-Y. Li, *Org. Biomol. Chem.* **2024**, *22*, 7596-7600.
24. (a) M. A. Cismesia, T. P. Yoon, *Chem. Sci.* **2015**, *6*, 5426-5434. (b) T. van Leeuwen, L. Buzzetti, L. A. Perego, P. Melchiorre, *Angew. Chem. Int. Ed.* **2019**, *58*, 4953-4957. (c) V. Ladányi, P. Dvořák, J. Al Anshori, L. U. Vetráková, J. Wirz, D. Heger, *Photochem. Photobiol. Sci.* **2017**, *16*, 1757-1761.
25. F. Li, D. Wang, H. Chen, Z. He, L. Zhou and Q. Zeng, *Chem. Comm.* **2020**, *56*, 13029-13032.
